# The risk of radiation-associated second cancer in patients with cervical cancer following radiotherapy from 1975 to 2019

**DOI:** 10.1093/oncolo/oyaf334

**Published:** 2025-10-10

**Authors:** Shanshan Wang, Xiaojing Yang, Zhongrong Gao, Mengli Zhao, Zhen Li, Yuan Lu, Hua Jiang, Keqin Hua, Jie Fu

**Affiliations:** Department of Radiation Oncology, Shanghai Sixth People’s Hospital Affiliated to Shanghai Jiao Tong University School of Medicine, Shanghai 200233, China; Department of Radiation Oncology, Shanghai Sixth People’s Hospital Affiliated to Shanghai Jiao Tong University School of Medicine, Shanghai 200233, China; Department of Radiation Oncology, Shanghai Sixth People’s Hospital Affiliated to Shanghai Jiao Tong University School of Medicine, Shanghai 200233, China; Department of Radiation Oncology, Shanghai Sixth People’s Hospital Affiliated to Shanghai Jiao Tong University School of Medicine, Shanghai 200233, China; Department of Radiation Oncology, Shanghai Sixth People’s Hospital Affiliated to Shanghai Jiao Tong University School of Medicine, Shanghai 200233, China; Department of Gynecology, Obstetrics and Gynecology Hospital of Fudan University, Shanghai 200011, China; Department of Gynecology, Obstetrics and Gynecology Hospital of Fudan University, Shanghai 200011, China; Department of Gynecology, Obstetrics and Gynecology Hospital of Fudan University, Shanghai 200011, China; Department of Radiation Oncology, Shanghai Sixth People’s Hospital Affiliated to Shanghai Jiao Tong University School of Medicine, Shanghai 200233, China

**Keywords:** second cancers, radiotherapy, cervical cancer, SEER

## Abstract

**Background:**

Radiation-associated second primary malignancies (SPMs) are a significant risk factor affecting the quality of life in long-term cervical cancer survivors. However, the impact of brachytherapy-boost and advanced radiotherapy techniques on the risk of radiation-related SPMs remains unclear.

**Methods:**

We utilized data from the Surveillance, Epidemiology, and End Results (SEER) database (1975-2019) to assess the risk of radiation-associated SPMs among cervical cancer patients. Radiation-associated second solid and hematologic malignancies were defined as those diagnosed in survivors living for ≥5 and ≥2 years, respectively. The fine-gray sub-distribution hazard model was employed to compare the risk of SPMs across different groups.

**Results:**

External beam radiation therapy (EBRT) was associated with an increased risk of pelvic SPMs (sub-distribution hazard ratio [sHR] = 2.13; *P *< .001). However, no increased risk was observed for extra-pelvic or hematologic SPMs. For radiotherapy-treated patients, the 15-year cumulative incidence of overall pelvic SPMs significantly declined from 3.92% in 1975-1994 to 2.85% in 1995-2006 (sHR = 0.87; *P *= .036), further decreasing to 2.27% after 2001 compared to those treated in 1975-2001 (sHR = 0.59; *P *= .030). Brachytherapy alone increased the risk of pelvic SPMs (sHR = 3.04; *P *< .001), but the combination of brachytherapy with EBRT did not further elevate the risk of pelvic SPMs (sHR = 1.35; *P* = .092).

**Conclusions:**

The risk of radiation-associated pelvic SPMs has diminished over the past 40 years, and the combination of brachytherapy with EBRT did not further increase the risk of SPMs among cervical cancer patients.

Implications for practice Radiotherapy elevates the risk of second primary malignancies (SPMs) in survivors of cervical cancer. However, it remains unclear whether advancements in external beam radiation therapy (EBRT) techniques have influenced the risk of SPMs in cervical cancer patients. Our study suggested that improvements in EBRT reduced the risk of second pelvic cancers in cervical cancer survivors, and the addition of brachytherapy did not further increase the risk of SPMs. This study underscored the necessity for ongoing advancements in radiotherapy to mitigate long-term risks of second cancer.

## Introduction

Over 50% of newly diagnosed cervical cancer patients received radiation therapy as a primary treatment.^1^^,^^2^ The 5-year survival rates were over 90% for early-stage and 70% for locally advanced cases.^3^^,^^4^ As survival rates for cervical cancer significantly improved, radiation-related second primary malignancies (SPMs) emerged as a growing concern. Previous studies have suggested that cervical cancer patients who underwent external beam radiation therapy (EBRT) had an increased risk of SPMs compared to non**-**radiotherapy patients.^5-10^ Due to higher radiation exposure, pelvic organs were at greater risk for SPMs than non-pelvic organs, with nearby organs such as the bladder, rectum, and ovaries facing an elevated risk.^11^^,^^12^ EBRT advanced significantly over the past 4 decades.^13^ Three-dimensional conformal radiotherapy (3D-CRT) improved target coverage while reducing radiation exposure to nearby organs, becoming the standard of care by the late 1990s. The introduction of intensity-modulated radiation therapy (IMRT) further enhanced normal tissue sparing and clinical outcomes compared to 3D-CRT.^14^^,^^15^ The proportion of radiation oncologists using IMRT for genitourinary tumors increased from 32% in 2002 to 73% in 2004.^16^ However, the impact of advancements in radiotherapy techniques, particularly IMRT, on the development of SPMs remained controversial. IMRT was thought to increase the risk of SPMs due to its use of multiple beam angles, increased internal scatter, and linear accelerator head leakage, which resulted in greater low-dose radiation exposure to surrounding normal tissues.^17-19^ Nonetheless, recent large cohort studies suggested that IMRT treatment for prostate cancer was not associated with an increased risk of SPMs compared to 3D-CRT.^20^

Brachytherapy remains essential in the treatment of locally advanced cervical cancer and positive surgical margins.^2^ Research on the risk of SPMs caused by brachytherapy in gynecological cancers remains scarce. While some studies suggest no increased risk of SPMs with brachytherapy in endometrial cancer, others have shown higher SPM rates in uterine corpus cancer patients receiving radiotherapy compared to surgery alone.^21^^,^^22^ Given the limited data, further research into brachytherapy-related SPMs in cervical cancer is necessary.

Although prior studies have explored the risk of SPMs in cervical cancer patients treated with radiotherapy, the existing research on this topic remains limited. The impact of advancements in radiotherapy techniques on SPMs, particularly the role of brachytherapy, has been minimally investigated in cervical cancer. Therefore, this study was conducted to determine whether the introduction and widespread adoption of new technologies have reduced the risk of SPMs post-radiotherapy and to investigate whether the addition of brachytherapy increases the risk of SPMs in cervical cancer.

## Materials and methods

### Database and participants

Patients diagnosed with cervical cancer as their first primary cancer were identified from 8 registries of the Surveillance, Epidemiology, and End Results (SEER) database between January 1, 1975, and December 31, 2019. All primary cancer sites were coded according to the International Classification of Diseases for Oncology, Third Edition (ICD-O-3). The included patients were pathologically diagnosed with cervical cancer (ICD-O-3: C53.0, C53.1, C53.8, and C53.9). Staging was assigned using the extent of disease codes, stage, and tumor size variables according to the 2009 International Federation of Gynecologists and Obstetricians (FIGO) staging system for cervical cancer. Exclusion criteria included patients whose cervical cancer was not their first primary cancer, aged under 20 or over 90, with distant-stage disease, survival less than 2 years post-diagnosis, or missing data on radiotherapy, age, survival, and follow-up. Those with hematologic malignancies within 2 years or a second primary solid tumor within 5 years post-diagnosis were also excluded (Figure S1, see online supplementary material for a color version of this figure). This study used public data without personal identifiers and therefore required neither informed consent nor approval from an institutional review board.

### Definition and follow-up of SPMs

The primary outcome of this study was the development of SPMs, defined as any solid malignancy occurring over 5 years after cervical cancer treatment or any hematologic malignancy occurring over 2 years post-treatment.^23^ Follow-up for second hematologic malignancies began 2 years post–cervical cancer diagnosis, concluding with the occurrence of a second hematologic malignancy, all-cause death, or after 35 years, whichever occurred first. Similarly, follow-up for second solid malignancies commenced 5 years post-diagnosis and ended upon the occurrence of a second solid malignancy, all-cause death, or after 40 years, whichever occurred first.

### Statistical analysis

Data analysis was conducted between September 20, 2024, and December 20, 2024. All statistical analyses were performed using R software, version 4.4.0. Categorical data were analyzed using the χ^2^ test or Fisher’s exact test when frequencies were less than 5. Continuous variables were assessed using the Mann–Whitney test, accommodating both normal and non-normal distributions. The comparison of hazard ratios (HRs) between the EBRT group and the no/unknown EBRT group was performed using fine-gray sub-distribution hazard ratio (sHR) models. All-cause mortality was treated as a competing event when calculating sHRs and 95% CIs for the occurrence of SPMs. Age-adjusted incidence rates, along with the corresponding annual percent changes (APC) and average annual percent changes (AAPC), were calculated using SEER*Stat software (version 8.4.3) and Joinpoint models through the Joinpoint Regression Program (version 4.9.1.0; National Cancer Institute, Bethesda, Maryland). Cervical cancer incidence rates were standardized to the 2000 US population and expressed per 100 000 females. Propensity score matching (PSM) was employed to balance baseline characteristics across clinically relevant sub−groups of interest. To further assess dynamic risks and the incidence of SPMs related to radiotherapy, we calculated sHRs stratified by latency time since cervical cancer diagnosis, age at diagnosis, and year of diagnosis.

## Results

### Baseline characteristics and the utilization of radiotherapy in cervical cancer patients

A total of 27 565 cervical cancer patients were identified. The EBRT group consisted of 11 503 patients (41.7%), with a median age of 52 years and a median follow-up duration of 10.5 years (Table 1). The non-EBRT group included 16 062 patients (58.3%), with a median age of 40 years and a median follow-up duration of 19.3 years. Between 1975 and 2019, 9.6% of the EBRT group and 7.7% of the non-EBRT group developed second primary solid malignancies (Table 1).

**Table 1.** Comparison of characteristics of cervical cancer patients in the SEER 8 (1975-2021) database by EBRT use.

Characteristics	EBRT, no. (%)	No or unknown EBRT, no. (%)	*P*-value
**Total**	11 503 (41.7)	16 062 (58.3)	-
**Age at cervical cancer diagnosis, median (IQR), years**	52 (41-63)	40 (33-50)	<.001^a^
**Age at cervical cancer diagnosis, years**			<.001^b^
** 20-35**	1375 (12.0)	5530 (34.4)	
** 36-50**	4137 (36.0)	6687 (41.6)	
** 51-65**	3669 (31.9)	2678 (16.7)	
** 65+**	2322 (20.2)	1167 (7.3)	
**Years of cervical cancer diagnosis, median (IQR)**	1995 (1984-2008)	1996 (1987-2007)	<.001^a^
**Years of cervical cancer diagnosis**			<.001^b^
** 1975-1984**	3034 (26.4)	3232 (20.1)	
** 1985-1994**	2591 (22.5)	4068 (25.3)	
** 1995-2005**	2528 (22.0)	4324 (26.9)	
** 2006-2016**	3350 (29.1)	4438 (27.6)	
**Race**			<.001^b^
** Hispanic**	1507 (13.1)	1855 (11.5)	
** Non–Hispanic Black**	1255 (10.9)	1425 (8.9)	
** Non–Hispanic White**	7160 (62.2)	10 845 (67.5)	
** Others**	1581 (13.8)	1937 (12.1)	
**Marital status**			<.001^b^
** Married**	5539 (48.2)	8425 (52.5)	
** Divorced**	1586 (13.8)	1942 (12.1)	
** Single**	1856 (16.1)	3227 (20.1)	
** Widowed**	1758 (15.3)	943 (5.9)	
** Unknown**	764 (6.6)	1525 (9.5)	
**Residence**			<.001^b^
** Large metro**	3685 (32.0)	5582 (34.8)	
** City**	2241 (19.5)	3580 (22.3)	
** Urban**	566 (4.9)	729 (4.5)	
** Rural**	495 (4.3)	773 (4.8)	
** Unknown**	4516 (39.3)	5398 (33.6)	
**Income**			<.001^b^
** ≥$100 000**	1165 (10.1)	1968 (12.3)	
** $85 000-$100 000**	2245 (19.5)	3567 (22.2)	
** $70 000-$8500**	2072 (18.0)	3178 (19.8)	
**<$55 000-$70 000**	1643 (14.3)	2249 (14.0)	
** Unknown**	4378 (38.1)	5100 (31.8)	
**Follow-up time of patients with cervical cancer, years, median (IQR)**	10.5 (4.7-20.1)	19.3 (9.4-29.1)	<.001^a^
**Follow-up time of patients with cervical cancer, years**			<.001^b^
** 2-5**	3125 (27.2)	1886 (11.7)	
** 5-10**	2457 (21.4)	2413 (15.0)	
** 10-20**	3020 (26.3)	4004 (24.9)	
** ≥20**	2901 (25.2)	7759 (48.3)	
**No. of cases (any SPM)**	1254 (10.9)	1425 (8.9)	<.001^b^
**No. of cases (classified SPM)**			<.001^b^
** Solid cancer (confined to the pelvis)**	462 (4.1)	313 (1.9)	
** Solid cancer (out of the pelvis)**	637 (5.5)	936 (5.8)	
** Hematologic malignancies**	101 (0.9)	137 (0.9)	
** Malignancies (unknown type)**	54 (0.4)	39 (0.3)	
**Attained age at event (any SPM), median (IQR), years**	68 (60-77)	63 (54-72)	<.001^a^
**Latency between cervical cancer and event (any SPM), years, median (IQR)**	13.1 (8.4-20.1)	16.6 (10.1-24.3)	<.001^a^
**FIGO staging (Version 2009)**			<.001^b^
** I**	4117 (35.8)	12 110 (75.4)	
** II**	3171 (27.6)	443 (2.8)	
** III/IV**	1410 (12.3)	128 (0.8)	
** Unknown**	2805 (24.4)	3381 (21.0)	
**Lymph node metastases**			<.001^b^
** Yes**	1968 (17.1)	247 (1.5)	
** No**	5352 (46.5)	11 115 (69.2)	
** Unknown**	4183 (36.4)	4700 (29.3)	
**Distant metastases**			<.001^b^
** M0**	7421 (64.5)	11 178 (69.6)	
** Unknown**	4082 (35.5)	4884 (30.4)	
**Histological type**			<.001^b^
** Squamous cell carcinoma**	8779 (76.3)	10 508 (65.4)	
** Adenocarcinoma**	1302 (11.3)	2848 (17.7)	
** Other/unknown**	1422 (12.4)	2706 (16.8)	
**Grade**			<.001^b^
** I**	738 (6.4)	1903 (11.8)	
** II**	3371 (29.3)	3481 (21.7)	
** III/IV**	3587 (31.2)	2384 (14.9)	
** Other/unknown**	3807 (33.1)	8294 (51.6)	
**Surgery**			<.001^b^
** Yes**	5037 (43.8)	14 742 (91.8)	
** No/unknown**	6466 (56.3)	1320 (8.2)	
**Brachytherapy**			<.001^b^
** Yes**	7726 (67.2)	509 (3.2)	
** No**	3777 (32.8)	15 553 (96.8)	
**Chemotherapy**			
** Yes**	4582 (39.8)	540 (3.4)	<.001^b^
** No/unknown**	6921 (60.2)	15 522 (96.6)	

Abbreviations: EBRT, external beam radiotherapy; FIGO, Federation of Gynecologists and Obstetricians; IQR, interquartile range; SPM, secondary primary malignancy.

a *P*-values were calculated using the Mann–Whitney test for continuous variables and χ^2^ test.

b *P*-values were calculated using the Mann–Whitney test for categorical variables.

**Figure 1.**
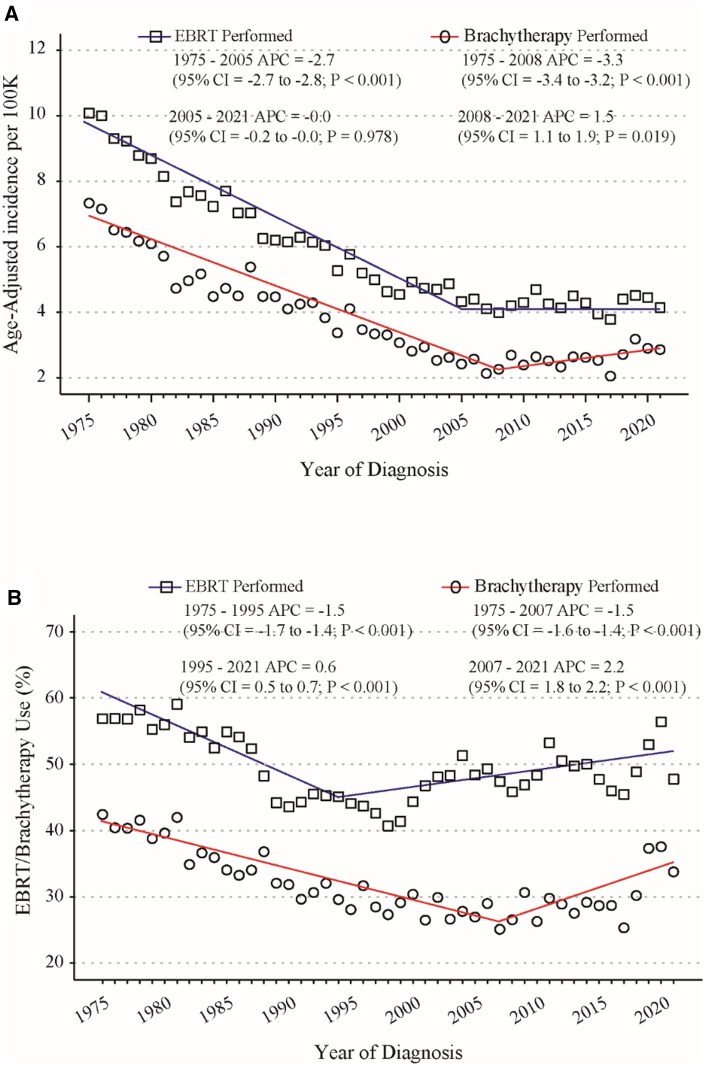
Trends in the use of external beam radiation therapy (EBRT) and brachytherapy for cervical cancer patients: analysis of the SEER 8 database (1975-2021). (A) Age-adjusted incidence trends over time for the use of EBRT and brachytherapy in cervical cancer patients in the SEER 8 database (1975-2021). (B) Trends in the percentage of EBRT and brachytherapy usage in cervical cancer patients in the SEER 8 database (1975-2021). Age-adjusted incidence rates for EBRT and brachytherapy were calculated using SEER*Stat software, with the rates standardized to the 2000 US population. Corresponding annual percent changes (APC) were determined using Joinpoint software.

We further analyzed the usage of EBRT and brachytherapy in the United States from 1975 to 2021 (Figure 1). The results indicated that the incidence rates of cervical cancer patients treated with EBRT remained stable, ranging from 4.3 per 100 000 females in 2005 to 4.1 per 100 000 females in 2021 (APC: 0.0, 95% CI: −0.2-0.0; *P *= .978; Figure 1A). In contrast, the incidence of patients receiving brachytherapy significantly increased from 2.3 per 100 000 females in 2008 to 2.8 per 100 000 females in 2021 (APC: 1.5, 95% CI: 1.1-1.9; *P *= .019; Figure 1A). A specific analysis of patients diagnosed with cervical cancer in the SEER 8 database revealed that the proportion of cervical cancer patients receiving EBRT increased significantly from 44.0% in 1995 to 48.0% in 2021 (APC: 0.6, 95% CI: 0.5-0.7; *P *< .001; Figure 1B). Similarly, the proportion of cervical cancer patients treated with brachytherapy rose significantly from 25.0% in 2007 to 34.0% in 2021 (APC: 2.2, 95% CI: 1.8-2.2; *P *< .001; Figure 1B).

### Impact of EBRT on the occurrence of SPMs in cervical cancer patients

Second primary solid tumors were classified based on their anatomical locations into pelvic and non-pelvic tumors (Table S1). Our analysis revealed a significant association between EBRT and an increased risk of overall second primary pelvic tumors, with a sHR of 2.13 (95% CI: 1.81-2.50, *P < *.001; Table 2). In contrast, no significant association was found between EBRT and the risk of overall non-pelvic tumors (sHR = 1.03, 95% CI: 0.92-1.15, *P = *.590) or overall hematologic malignancies (sHR = 0.93, 95% CI: 0.72-1.21, *P = *.600; Table 2). When examining specific types of pelvic tumors, EBRT was associated with a significantly increased risk of several malignancies, including bladder cancer (sHR = 2.40), rectal cancer (sHR = 3.11), colon cancer (sHR = 1.55), ovarian cancer (sHR = 3.84), corpus carcinoma (sHR = 9.97), and vulvar cancer (sHR = 4.70; Table 2). However, the risk of small bowel cancer, vaginal cancer, and anal cancer did not show a significant increase (Table 2). Among non-pelvic tumors, EBRT was significantly linked to an increased risk of lung cancer (sHR = 1.45, 95% CI: 1.20-1.75, *P < *.001; Table 2) while being associated with a decreased risk of breast cancer (sHR = 0.68, 95% CI: 0.56-0.82, *P < *.001; Table 2). No significant association was identified between EBRT and the risk of any specific hematologic malignancy.

**Table 2.** Comparisons of cumulative incidence of second primary malignancies (SPMs) between cervical cancer patients who received external beam radiation therapy (EBRT) and those who did not receive EBRT.

Second primary malignancy sites	EBRT vs. no/unknown-EBRT	Univariable competing risks regression model		Univariable competing risks regression model (after PSM)^b^	
	No. of patients with events	sHR (95% CI)	*P*-value	sHR (95% CI)	*P*-value
**All solid cancers (within pelvis)**	462/313	2.13 (1.81-2.50)	<.001	1.90 (1.64-2.21)	<.001
** Urinary bladder**	73/43	2.40 (1.58-3.66)	<.001	2.07 (1.42-3.04)	<.001
** Rectum**	61/32	3.11 (1.88-5.16)	<.001	2.85 (1.78-4.54)	<.001
** Colon, NOS**	125/107	1.55 (1.17-2.04)	.002	1.51 (1.16-1.96)	.002
** Small intestine**	10/5	3.37 (0.93-12.26)	.065	2.28 (0.78-6.7)	.130
** Ovary**	45/24	3.84 (2.03-7.26)	<.001	2.35 (1.41-3.93)	.001
** Corpus uteri**	49/9	9.97 (3.97-25.03)	<.001	6.35 (3.12-12.91)	<.001
** Vagina**	23/28	1.01 (0.57-1.80)	.970	1.04 (0.59-1.84)	.890
** Vulva**	28/14	4.70 (1.95-11.37)	.001	3.13 (1.53-6.42)	.002
** Anal canal**	6/26	0.41 (0.16-1.05)	.062	0.33 (0.13-0.82)	.017
** Total of others^a^**	42/25	3.05 (1.67-5.58)	<.001	2.19 (1.32-3.66)	.002
**All solid cancers (out of pelvis)**	637/936	1.03 (0.92-1.15)	.590	0.92 (0.83-1.02)	.130
** Breast**	182/413	0.68 (0.56-0.82)	<.001	0.6 (0.5-0.72)	<.001
** Lung and bronchus**	264/255	1.45 (1.20-1.75)	<.001	1.37 (1.14-1.63)	.001
** Kidney and renal pelvis**	19/40	0.71 (0.40-1.28)	.260	0.63 (0.36-1.1)	.100
** Pancreas**	25/29	1.33 (0.73-2.42)	.350	1.08 (0.63-1.86)	.770
** Thyroid**	15/35	0.80 (0.41-1.57)	.510	0.69 (0.36-1.31)	.260
** Stomach**	25/22	1.59 (0.85-2.98)	.150	1.45 (0.8-2.6)	.220
** Melanoma**	16/30	0.85 (0.44-1.66)	.640	0.82 (0.43-1.55)	.540
** Liver**	5/16	0.42 (0.15-1.19)	.100	0.38 (0.14-1.02)	.056
** Larynx**	5/12	0.56 (0.19-1.67)	.300	0.52 (0.18-1.48)	.220
** Brain**	6/10	0.68 (0.24-1.90)	.460	0.71 (0.26-1.94)	.510
** Esophagus**	8/8	1.62 (0.53-4.95)	.390	1.19 (0.43-3.26)	.730
** Total of others^a^**	67/66	1.55 (1.06-2.26)	.024	1.35 (0.95-1.92)	.094
**All hematologic malignancies**	101/137	0.87 (0.67-1.13)	.290	0.93 (0.72-1.21)	.600
** Lymphoma**	57/67	0.99 (0.69-1.42)	.940	1.08 (0.75-1.55)	.680
** Lymphocytic leukemia**	7/18	0.51 (0.21-1.27)	.150	0.49 (0.2-1.18)	.110
** Non-lymphocytic leukemia**	20/15	1.70 (0.83-3.46)	.140	1.77 (0.89-3.49)	.100
** Myeloma**	10/15	0.85 (0.37-1.97)	.710	0.83 (0.37-1.85)	.650
** Total of others^a^**	7/22	0.33 (0.14-0.76)	.010	0.39 (0.17-0.93)	.034

a In addition to the cancers listed in this table, other cancer types also include pelvic tumors, extra pelvic tumors, and hematologic malignancies with fewer than 15 total cases (see Table S1 for details).

b To minimize biases related to treatment assignment, patients in the no/unknown EBRT group were matched to their nearest neighbors in a 1:1 ratio without replacement based on propensity scores. This approach aimed to balance the baseline characteristics between the EBRT and no/unknown EBRT groups. Abbreviations: EBRT, external beam radiotherapy; sHR, Sub-distribution hazard ratio; CI, confidence interval; PSM, propensity score matching; NOS, not otherwise specified.

The impact of EBRT on SPMs was further evaluated by comparing the incidence rates of these tumors between the PSM groups (*n* = 11 503 per group; Table S2). Upon examining the PSM results, we observed a significant improvement in the balance of baseline characteristics between the 2 groups after matching (Figure S2, see online supplementary material for a color version of this figure). However, notable differences in baseline characteristics remained (Table S2). To further account for potential confounding variables influencing the development of SPMs, we conducted both univariable and multivariable fine-gray competing risk regression analyses (see Tables S3-S10). These models consistently identified age, year of diagnosis, and receipt of EBRT as independent risk factors for SPMs. Importantly, EBRT was significantly associated with an increased risk of pelvic in-field second malignancies in both univariable and multivariable fine-gray competing risk regression models. In contrast, no statistically significant association was observed for overall extra-pelvic solid tumors or hematologic malignancies (see Tables S3-S10). These site-specific results were further supported by multivariable competing risk models for individual second malignancy types (see Table S11), which aligned closely with the findings from the univariable analysis (Table 2). Moreover, analyses performed in the propensity score–matched cohort (EBRT group vs. no/unknown EBRT group) produced results that were highly consistent with those observed in the unmatched cohort, across both univariable and multivariable competing risk models (Table 2 and Tables S3-S11). This confirmed the association of EBRT with an increased risk of second primary pelvic tumors and lung cancer, along with a decreased risk of breast cancer (Table 2 and Table S11). Furthermore, sub−group analyses across different clinical characteristics demonstrated that the impact of EBRT on the risk of pelvic SPMs remained consistent across all sub−groups (Figure S3, see online supplementary material for a color version of this figure).

### Cumulative incidence of SPMs in cervical cancer patients who received EBRT

In patients who underwent EBRT, the cumulative incidence of second primary pelvic solid tumors was assessed over various time intervals post-diagnosis (Table 3). The cumulative incidence within 5-10 years after the initial diagnosis was found to be 1.81% (95% CI: 1.52%-2.15%). This increased to 3.56% (95% CI: 3.14%-4.05%) for the 5-15 years interval and reached 8.20% (95% CI: 7.46%-9.02%) within 5-30 years post-diagnosis (Table 3). Among the patients treated with EBRT, the highest cumulative incidence of second primary pelvic malignancies occurring within 5-15 years after the initial cancer diagnosis was observed for colon cancer, with an incidence of 0.90% (95% CI: 0.69%-1.17%). This was followed by uterine malignancies (0.51%, 95% CI: 0.36%-0.73%), bladder cancer (0.45%, 95% CI: 0.33%-0.67%), and rectal cancer (0.44%, 95% CI: 0.30%-0.64%; Table 3).

**Table 3.** Cumulative incidence of second primary malignancies (SPMs) in patients with cervical cancer following external beam radiation therapy (EBRT).

Second primary malignancy sites	Cumulative incidence (5-10 years, 95% CI)	Cumulative incidence (5-15 years, 95% CI)	Cumulative incidence (5-30 years, 95% CI)	Cumulative incidence (5-40 years, 95% CI)
**All solid cancers (within pelvis)**	1.81% (1.52%-2.15%)	3.56% (3.14%-4.05%)	8.20% (7.46%-9.02%)	9.98% (9.06%-1.10%)
**Urinary bladder**	0.17% (0.09%-0.30%)	0.45% (0.33%-0.67%)	1.41% (1.10%-1.81%)	1.93% (1.50%-2.50%)
**Rectum**	0.21% (0.12%-0.35%)	0.44% (0.30%-0.64%)	1.19% (0.90%-1.56%)	1.49% (1.13%-1.96%)
**Colon, NOS**	0.51% (0.37%-0.72%)	0.90% (0.69%-1.17%)	2.51% (2.09%-3.02%)	2.79% (2.31%-3.36%)
**Ovary**	0.14% (0.07%-0.27%)	0.30% (0.19%-0.48%)	0.90% (0.65%-1.23%)	1.21% (0.86%-1.70%)
**Corpus uteri**	0.23% (0.14%-0.38%)	0.51% (0.36%-0.73%)	0.89% (0.67%-1.21%)	1.04% (0.77%-1.40%)
**Vulva**	0.24% (0.15%-0.39%)	0.36% (0.24%-0.56%)	0.48% (0.33%-0.72%)	0.58% (0.37%-0.93%)
**Breast**	1.10% (0.87%-1.37%)	1.81% (1.51%-2.19%)	3.27% (2.81%-3.81%)	3.64% (3.12%-4.50%)
**Lung and bronchus**	1.53% (1.27%-1.85%)	2.79% (2.41%-3.22%)	4.61% (4.07%-5.22%)	5.07% (4.45%-5.78%)

Abbreviations: CI, confidence interval; NOS, not otherwise specified.

In terms of non-pelvic malignancies, lung cancer emerged as the most common second primary solid tumor, with a cumulative incidence of 2.79% (95% CI: 2.41%-3.22%) within 5-15 years following the initial cancer diagnosis. Breast cancer followed as the second most prevalent non-pelvic malignancy, with a cumulative incidence of 1.81% (95% CI: 1.51%-2.19%; Table 3) within 5-15 years post-diagnosis.

### Latency and age: impact on the risk of second primary tumors in cervical cancer patients post-EBRT therapy

In patients who developed second primary pelvic tumors following EBRT, it was observed that 50% of these tumors occurred within 15 years after the administration of radiation (Figure S4, see online supplementary material for a color version of this figure). The highest probability of occurrence was noted at approximately 9.4 years post-treatment, with a peak incidence rate of 5.1%. After this point, the likelihood of developing these tumors gradually declined (Figure S4, see online supplementary material for a color version of this figure).

The analysis of dynamic latency-sHRs revealed that the risk of overall pelvic tumors and colorectal cancers was highest within the first 15 years post-diagnosis, with sHRs of 5.10 and 5.23, respectively, when comparing patients who received EBRT to non-EBRT (Figure S5, see online supplementary material for a color version of this figure). Following this peak, the risk of these tumors showed a gradual decrease over time. In contrast, the sHRs for overall non-pelvic tumors tended to approach 1 as the follow-up period extended. Notably, the sHRs for bladder cancer and breast cancer exhibited a downward trend with increasing follow-up time, while the risk for lung cancer showed a slow but steady increase (Figure S5, see online supplementary material for a color version of this figure).

As patients aged at the time of diagnosis, the overall incidence rate of second primary pelvic tumors among those who received EBRT began to converge with that of patients who did not undergo EBRT (Figure S5, see online supplementary material for a color version of this figure). Specifically, the sHR for overall pelvic second tumors demonstrated a significant downward trend with increasing age at diagnosis. After the age of 65, the risk between the 2 groups no longer showed a significant difference, with an sHR of 1.28 (95% CI: 0.85-1.94, *P = *.229). For specific malignancies such as bladder cancer, rectal cancer, lung cancer, and breast cancer, the risk levels between the EBRT and non-EBRT groups also gradually approached each other as the age at diagnosis increased (Figure S5, see online supplementary material for a color version of this figure).

### Impact of year of diagnosis on the risk of second primary tumors in patients who received EBRT

**Figure 2.**
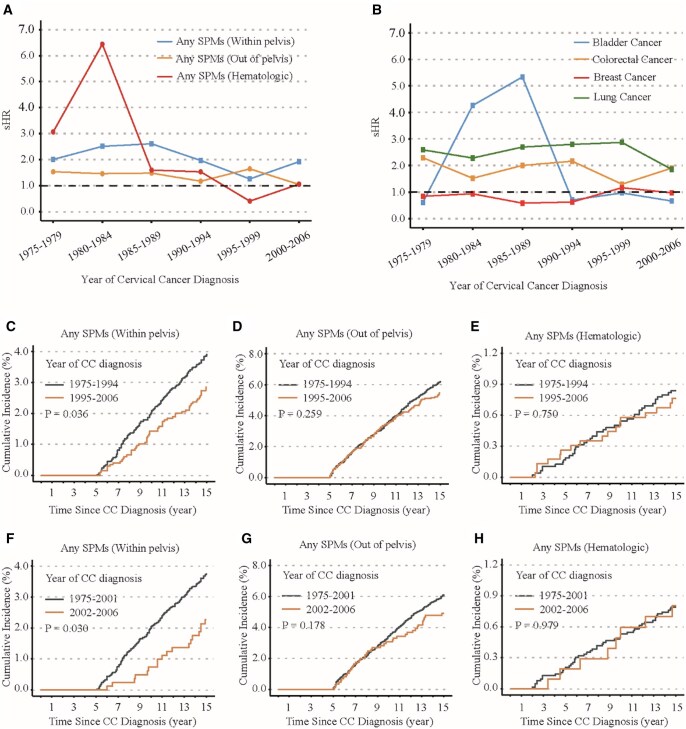
Trends in diagnosis year-related dynamic sub-distribution hazard ratios (sHRs) and cumulative incidence in cervical cancer patients. (A) Dynamic sHR (EBRT group vs. no or unknown EBRT group) for pelvic malignancies, extra-pelvic malignancies, and hematologic malignancies as depicted in the diagnosis year-sHR plot. (B) Dynamic sHR (EBRT group vs. no or unknown EBRT group) for bladder cancer, colorectal cancer, breast cancer, and lung cancer illustrated in the diagnosis year-sHR plot. Comparison of the unadjusted cumulative incidence of all second primary pelvic tumors (C), all extra-pelvic tumors (D), and all hematologic malignancies (E) within 15 years post-EBRT for patients diagnosed between 1975-1994 and 1995-2006. Comparison of the unadjusted cumulative incidence of all second primary pelvic tumors (F), all extra-pelvic tumors (G), and all hematologic malignancies (H) within 15 years post-EBRT for patients diagnosed between 1975-2001 and 2002-2006. EBRT, external beam radiation therapy; sHR, fine-gray competing-risk sub-distribution hazard ratio. Based on the SEER database submitted in November 2023, patients diagnosed after 2006 with a minimum follow-up time of less than 15 years will no longer be included in the comparison of 2/5- to 15-years cumulative incidence rates among patients diagnosed in different eras. Due to the differences in follow-up duration among patients diagnosed in different eras, for those with a follow-up time exceeding 15 years, the outcome status is uniformly defined as 0 (indicating that no events occurred).

To evaluate whether the risk of developing second primary tumors in patients receiving EBRT has changed over time, we conducted a statistical analysis of the cumulative incidence of second primary tumors in cervical cancer patients diagnosed between 1975 and 2006, specifically within the 2/5-15 years post-diagnosis period. Our findings revealed a notable trend: from 1990 onward, the risk ratios (15 years-sHRs) for overall second primary tumors in both EBRT and non-EBRT groups showed a declining trend (Figure 2A). Among patients treated with EBRT, the risk of developing hematologic malignancies also significantly decreased with advancements in the year of diagnosis. In contrast, the risk of developing overall non-pelvic tumors remained relatively stable over the same time period (Figure 2A). Notably, the risk of bladder cancer in cervical cancer patients who received EBRT significantly decreased after the 1990, showing no substantial difference compared to patients who did not receive EBRT (Figure 2B).

In our analysis of 8383 cervical cancer patients who received EBRT from 1975 to 2006, we found that the cumulative incidence of overall second pelvic tumors within 5-15 years post-diagnosis decreased significantly, from 3.92% (95% CI: 3.34%-4.59%) during 1975-1994 to 2.85% (95% CI: 2.19%-3.69%) during 1995-2006 (sHR = 0.72, 95% CI: 0.53-0.98, *P = *.036) (Figure 2C and Table S12). Similarly, the incidence of lung cancer dropped from 3.37% (95% CI: 2.83%-4.01%) in the earlier period to 2.23% (95% CI: 1.66%-3.01%) in the later period (sHR = 0.66, 95% CI: 0.47-0.94, *P = *.020) (Figure S6 [see online supplementary material for a color version of this figure] and Table S12). Furthermore, during the period of 2002-2006, the cumulative incidence rates for overall pelvic tumors and lung cancer further declined to 2.27% (95% CI: 1.44%-3.58%) and 1.62% (95% CI: 0.94%-2.77%), respectively (Figure 2F, Figure S6 [see online supplementary material for a color version of this figure], and Table S13). Conversely, among patients receiving EBRT, the incidence rates of overall non-pelvic solid tumors, hematologic malignancies, and breast cancer did not show significant changes between the 1990s and the early 2000s (Figure 2, Figures S6 and S7 [see online supplementary material for a color version of these figures], and Tables S12 and S13).

In summary, the risk of developing second primary pelvic tumors in patients who received EBRT has significantly decreased with advancements in the year of diagnosis. However, for patients who did not receive EBRT, the 5-15−year cumulative incidence rates of second solid and hematologic tumors did not exhibit any significant differences between those diagnosed after 1975 and those diagnosed after 2001 (Figures S8 and S9, see online supplementary material for a color version of these figures).

### The influence of brachytherapy addition on the incidence of SPMs in patients undergoing radical radiotherapy

Results from competing risk regression analyses indicated that cervical cancer patients who received brachytherapy alone demonstrated a significantly elevated risk of developing second primary pelvic tumors compared to those who did not undergo any form of radiation therapy (sHR = 3.04, 95% CI: 1.83-5.04, *P < *.001; Table S14). Specifically, the risk of bladder cancer and corpus carcinoma was markedly higher in patients treated exclusively with brachytherapy, with sHRs of 8.05 and 28.44, respectively (*P < *.001; Table S14). However, it was noteworthy that brachytherapy alone did not significantly increase the risk of any hematologic malignancies (sHR = 1.60, 95% CI: 0.66-3.91, *P = *.300; Table S14).

Furthermore, we examined whether the addition of brachytherapy to EBRT in cervical cancer patients further elevated the risk of developing second primary tumors. The results revealed that the incorporation of brachytherapy did not significantly increase the risk of any second primary pelvic tumors (sHR = 1.35, 95% CI: 0.95-1.93, *P = *.092; Table 4). Additionally, the risks of hematologic malignancies, breast cancer, and lung cancer were found to be comparable to those of cervical cancer patients who received only EBRT (Table 4).

**Table 4.** Cumulative incidence of second primary malignancies among patients receiving curative external beam radiation therapy (EBRT): comparison of brachytherapy use vs. no brachytherapy use.

Second primary malignancy sites	EBRT and brachytherapy vs. EBRT alone	Univariable competing risks regression model	
	No. of patients with events	sHR (95% CI)	*P*-value
**All solid cancers (within pelvis)**	255/35	1.35 (0.95-1.93)	.092
** Urinary bladder**	40/4	1.81 (0.65-5.07)	.260
** Ovary**	36/1	6.59 (0.91-47.71)	.062
** Rectum**	33/5	1.22 (0.48-3.11)	.680
** Colon, NOS**	63/11	1.08 (0.57-2.04)	.810
** Corpus uteri**	30/7	0.83 (0.37-1.86)	.640
** Vagina**	12/0	-	-
** Vulva**	13/2	1.36 (0.3-6.09)	.690
** Anus, anal canal and anorectum**	2/1	0.38 (0.04-3.8)	.410
** Total of others^a^**	22/4	1.13 (0.39-3.28)	.820
**All hematologic malignancies**	49/7	1.75 (0.79-3.84)	.170
** Lymphoma**	28/4	1.77 (0.62-5.02)	.280
** Lymphocytic leukemia**	4/1	0.95 (0.1-8.62)	.960
** Non-lymphocytic leukemia**	8/2	1.04 (0.22-4.8)	.960
** Myeloma**	4/0	-	-
** Total of others^a^**	5/0	-	-
**All solid cancers (out of pelvis)**	300/49	1.20 (0.89-1.63)	.230
** Breast**	77/11	1.38 (0.73-2.59)	.320
** Lung and bronchus**	129/21	1.24 (0.78-1.96)	.370

a In addition to the cancers listed in this table, other cancer types also include pelvic tumors, extra pelvic tumors, and hematologic malignancies with fewer than 15 total cases (see Table S1 for details).

Abbreviations: EBRT, external beam radiotherapy; sHR, Sub-distribution hazard ratio; CI, confidence interval; NOS, not otherwise specified.

## Discussion

As cancer survival rates improve, the incidence of second primary cancers has become a growing focus of clinical concern.^24^ The evaluation of second malignancy incidence post-radiotherapy has become increasingly important due to the rapid advancements in radiotherapy techniques for cervical cancer, which have significantly improved clinical outcomes over the past 40 years.^13-15^ IMRT, as an advanced radiotherapy technique, has been widely utilized in the treatment of cervical cancer.^14^^,^^16^ However, the debate over its association with the risk of secondary malignancies continues. While IMRT has been suspected to pose a higher carcinogenic risk compared to 3D-CRT, Ruben et al. found that the absolute risk of cancer induction is comparable between the two techniques, with minimal impact from low-dose diffusion on non-target tissues.^18^ Additionally, recent findings from a large population-based cohort study indicated that IMRT for prostate cancer was not associated with an increased risk of SPMs, whether solid tumors or hematologic cancers.^20^ As IMRT has gradually become the standard EBRT technique for cervical cancer, there remains a notable lack of large cohort studies investigating the risk of SPMs following EBRT. Our study found a significant decline in second pelvic tumors after radiotherapy with the introduction of advanced techniques. The 15-year incidence of pelvic SPMs was lower in patients treated after 1995 compared to those treated with conventional EBRT (1975-1994), and further decreased in those treated after 2001. Notably, our results indicated a notable decline in the risk of SPMs among patients diagnosed after the mid-1990s, especially in the 2000s and beyond. This trend likely reflects cumulative advances in radiotherapy planning, treatment delivery, and survivorship care. In particular, the increasing adoption of advanced techniques such as IMRT in the post–2000 era may have contributed to this decline.^16^^,^^25^ However, due to the lack of specific radiation modality data in the SEER database, the extent of IMRT’s impact cannot be directly assessed. Further studies incorporating detailed treatment information are needed to clarify the role of modern radiotherapy in reducing long-term SPMs risk.

The occurrence of SPMs following radiotherapy is influenced by various complex factors. This was first brought to attention by a multinational retrospective study conducted by Boice et al. in 1985, which reported a significantly increased risk of secondary cancers in organs adjacent to the cervix that were exposed to high doses of radiation.^26^ For non-pelvic malignancies, earlier research attributed a reduced breast cancer risk to radiation-induced ovarian damage, while the increased risk of lung cancer was hypothesized to result from misclassified metastases and confounding factors such as smoking.^26^^,^^27^ A more recent SEER study on cervical cancer reinforced this association, suggesting that much of the SPM risk is driven by the interaction between smoking and radiotherapy.^5^ Similarly, our study found a heightened risk of lung cancer in the EBRT group (sHR = 1.45; *P < *.001) and a decreased risk of breast cancer (sHR = 0.68; *P < *.001).

Chemotherapeutic agents are known to be important modifiers of SPMs risk. For cervical cancer, chemotherapy regimens are most commonly based on cisplatin. Since 1999, the National Cancer Institute has recommended cisplatin-based concurrent chemoradiation as standard treatment for cervical cancer. However, a recent meta-analysis reported no significant association between cisplatin use and increased risk of secondary cancers.^28^ In our study, the impact of chemotherapy on SPMs risk among cervical cancer survivors appeared minimal. Across multiple sub−group analyses, no significant association was observed between chemotherapy use and the risk of developing SPMs (Figure S10 [see online supplementary material for a color version of this figure] and Tables S3-S10 and S15-S21). Furthermore, when restricting the cohort to patients who did not receive chemotherapy, we still observed a significantly reduced risk of SPMs in patients diagnosed after 1999 compared to those diagnosed before, particularly during the 5-15 years survivorship window (Table S22). These findings suggested that cisplatin-based chemotherapy was unlikely to be a major confounding factor in the observed temporal decline in SPMs risk among cervical cancer survivors.

Studies on secondary malignancies associated with brachytherapy have yielded mixed results. Research on prostate cancer, for instance, found no significant difference in secondary malignancy risk between patients treated with EBRT alone and those receiving both EBRT and brachytherapy. However, some studies suggested that this combination might elevate the risk of bladder cancer in prostate cancer patients.^29^^,^^30^ Similarly, studies on endometrial and uterine cancers have produced inconsistent findings regarding the influence of brachytherapy on secondary malignancy risk.^21^^,^^22^ Prior studies on cervical cancer patients treated with high-dose-rate brachytherapy reported a notable increase in the risk of secondary malignancies.^9^ In contrast, our current study revealed that while brachytherapy alone significantly increased the risk of secondary pelvic malignancies (sHR = 3.04, *P < *.001), particularly bladder cancer (sHR = 8.05, *P < *.001), the addition of brachytherapy to EBRT did not significantly raise the risk of secondary pelvic or hematologic malignancies compared to EBRT alone in cervical cancer. This finding aligns with the understanding that at lower radiation doses (up to several Grays), the risk of secondary malignancies generally increases linearly with radiation exposure due to the survival and transformation of malignant cells.^31^ However, at higher doses, some models suggest that the risk plateaus or decreases, likely due to enhanced cell sterilization.^19^^,^^32^ Advancements in brachytherapy techniques have further shaped clinical outcomes and practice patterns. Previous research demonstrated that, compared to patients treated with 2D brachytherapy, 3D-image guided adaptive brachytherapy significantly improved survival rates and reduced gastrointestinal and genitourinary toxicities in cervical cancer patients.^15^

This study has several inherent limitations. The SEER database does not document specific radiotherapy techniques or differentiate between the extent of radiation fields. However, the rapid increase in IMRT utilization post-2002 has been well-documented. Additionally, the SEER database lacks information on smoking history, a factor known to contribute to the higher incidence of secondary tumors following EBRT.^5^^,^^12^ Previous research has demonstrated that even among non-smoking cervical cancer patients, the risk of secondary tumors in the EBRT group remains significantly higher than No-EBRT group.^9^ Importantly, our study found no differences in secondary tumor incidence between patients diagnosed before and after 2002 among No-EBRT group. Furthermore, given the large sample size of this study, we do not anticipate significant differences in smoking history across radiotherapy modalities or diagnosis years.

## Conclusion

Advancements in radiotherapy techniques contributed to a reduction in the risk of SPMs in cervical cancer patients. Importantly, the addition of brachytherapy did not significantly increase the risk of SPMs, and its combination with EBRT remained vital for enhancing survival outcomes in cervical cancer. Despite the potential risks of secondary malignancies, the benefits of modern radiotherapy techniques in managing cervical cancer significantly outweighed these risks, and our findings did not indicate a need to alter current clinical practice.

## Supplementary Material

oyaf334_Supplementary_Data

## Data Availability

The datasets used in this study are available in online repositories. The names of the repositories and the corresponding accession numbers can be found in the article or supplementary material.
