# Supplementary material for: The risk of radiation-associated second cancer in patients with cervical cancer following radiotherapy from 1975 to 2019
Source: Oncologist. 2025 Oct 10;30(11):oyaf334. doi: 10.1093/oncolo/oyaf334 (PMC12611298; doi:10.1093/oncolo/oyaf334)
Supplement: oyaf334_Supplementary_Data [file oyaf334_supplementary_data.zip › Supplementary Figure 3.docx]

**Supplementary Figure 3**


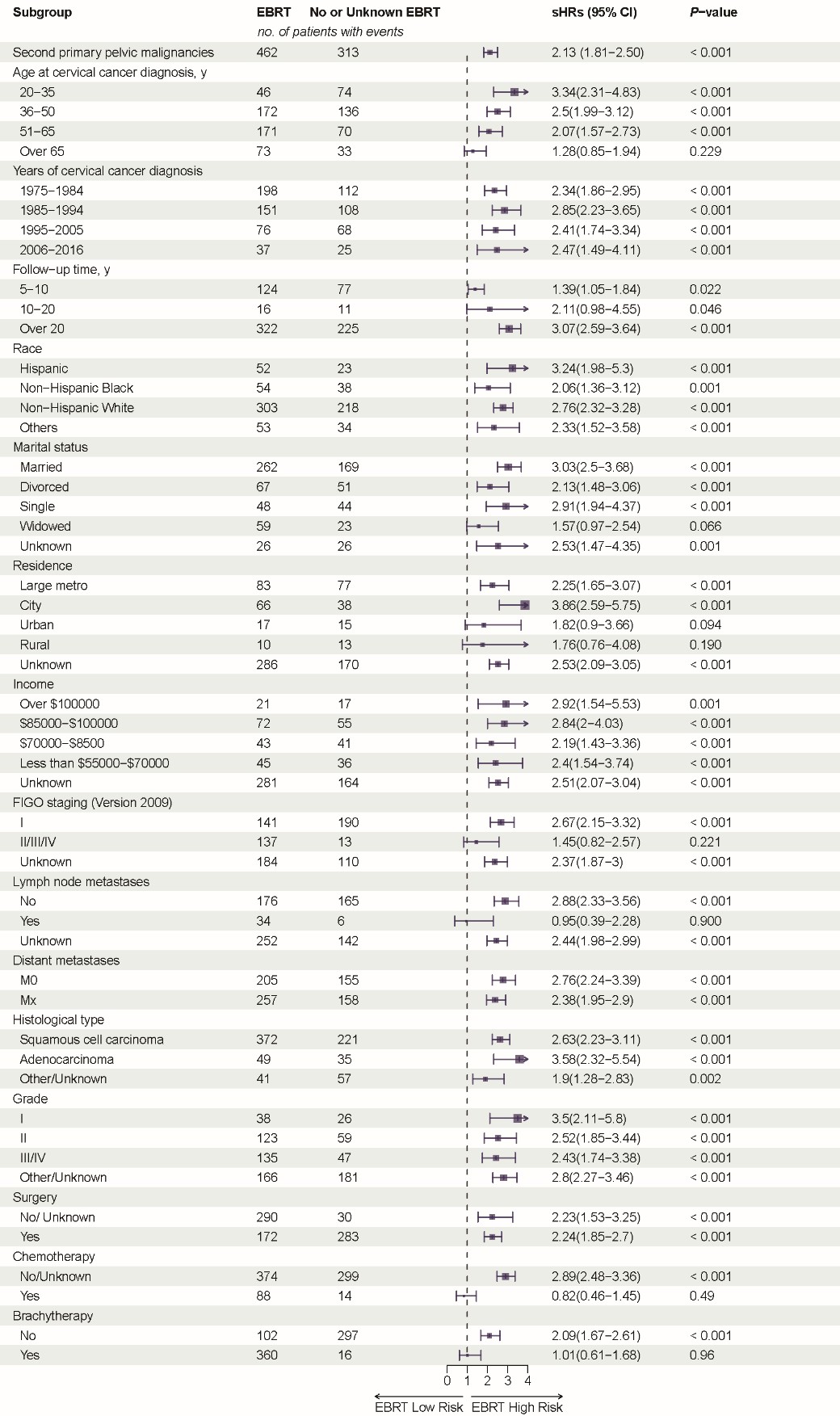


**Supplementary Figure 3.** Subgroup Analyses Using Fine and Gray’s Competing Risks Regression Model for the Risk of Developing Solid Pelvic Malignancies. This figure presents subgroup-specific subdistribution hazard ratios (sHRs) for the risk of secondary solid pelvic malignancies. The size of each box is proportional to the number of patients in the corresponding subgroup. Arrows at the ends of the confidence interval (CI) bars indicate that the lower or upper bounds extend beyond the displayed axis limits. Abbreviations: EBRT, external beam radiotherapy; sHRs, subdistribution hazard ratios.
