# Supplementary material for: The risk of radiation-associated second cancer in patients with cervical cancer following radiotherapy from 1975 to 2019
Source: Oncologist. 2025 Oct 10;30(11):oyaf334. doi: 10.1093/oncolo/oyaf334 (PMC12611298; doi:10.1093/oncolo/oyaf334)
Supplement: oyaf334_Supplementary_Data [file oyaf334_supplementary_data.zip › Supplementary Figure 4.docx]

**Supplementary Figure 4**


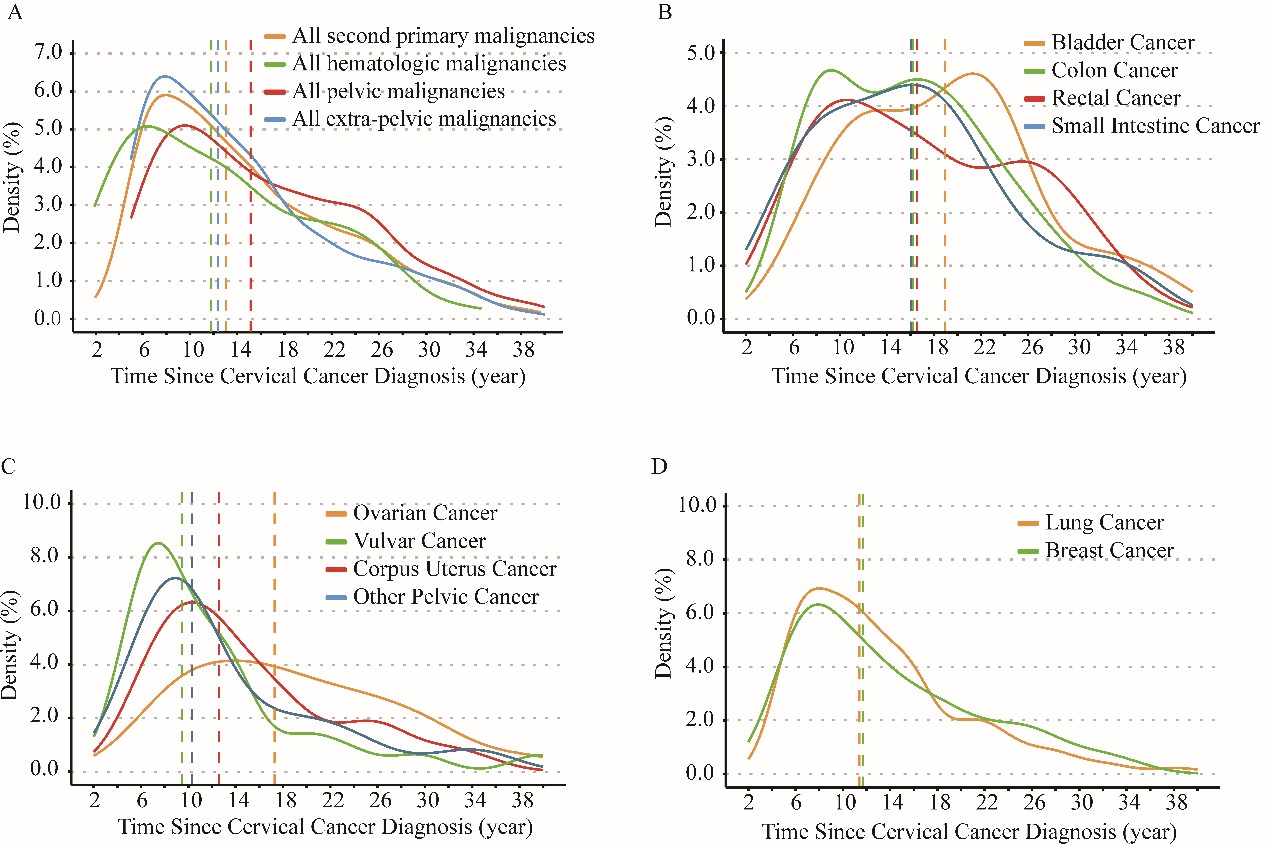


**Supplementary Figure 4.** Probability Density Distribution of Second Primary Malignancies Following EBRT. This figure presents the probability density distribution of second primary malignancies, with the x-axis representing the number of years post-radiotherapy and the y-axis indicating the percentage of cases relative to the total incidence of second primary malignancies. (A) Includes all second primary malignancies, all hematologic malignancies, all pelvic malignancies, and all extra-pelvic malignancies. (B) Focuses on bladder cancer, colon cancer, rectal cancer, and small intestine cancer. (C) Covers ovarian cancer, vulvar cancer, corpus uterus cancer, and other pelvic cancers. (D) Highlights lung cancer and breast cancer.

s
