# Supplementary material for: The risk of radiation-associated second cancer in patients with cervical cancer following radiotherapy from 1975 to 2019
Source: Oncologist. 2025 Oct 10;30(11):oyaf334. doi: 10.1093/oncolo/oyaf334 (PMC12611298; doi:10.1093/oncolo/oyaf334)
Supplement: oyaf334_Supplementary_Data [file oyaf334_supplementary_data.zip › Supplementary Figure 5.docx]

**Supplementary Figure 5**


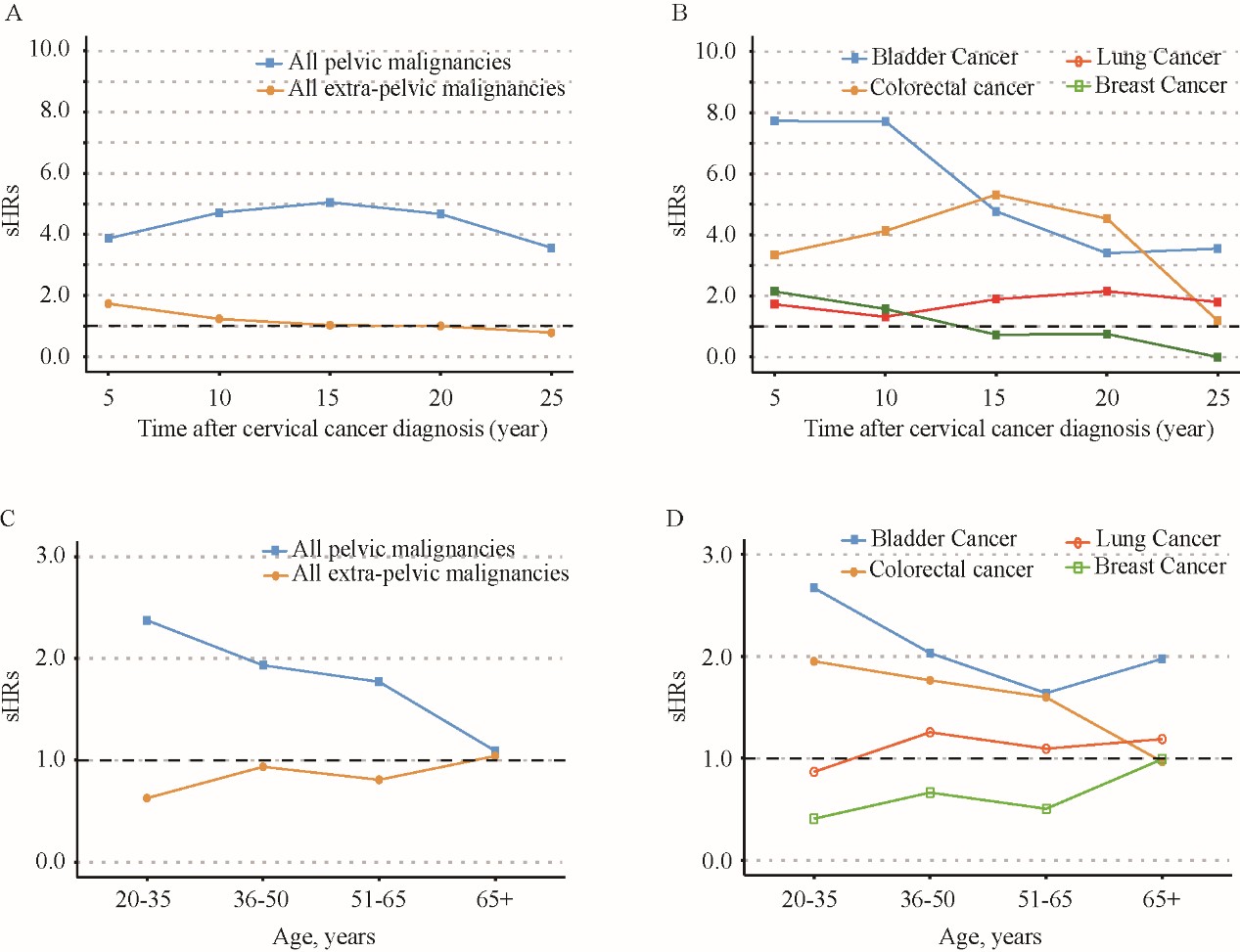


**Supplementary Figure 5.** Latency and Age-Related Dynamic Fine-Gray Competing-Risk Subdistribution Hazard Ratios (sHRs). (A) Dynamic sHRs for pelvic malignancies and extra-pelvic malignancies shown in the latency- sHRs plot. (B) Dynamic sHRs for bladder cancer, colorectal cancer, breast cancer, and lung cancer illustrated in the latency- sHRs plot. (C) Dynamic sHRs for pelvic malignancies and extra-pelvic malignancies shown in the age- sHRs plot. (D) Dynamic sHRs for bladder cancer, colorectal cancer, breast cancer, and lung cancer illustrated in the age- sHRs plot.
