# Supplementary material for: The risk of radiation-associated second cancer in patients with cervical cancer following radiotherapy from 1975 to 2019
Source: Oncologist. 2025 Oct 10;30(11):oyaf334. doi: 10.1093/oncolo/oyaf334 (PMC12611298; doi:10.1093/oncolo/oyaf334)
Supplement: oyaf334_Supplementary_Data [file oyaf334_supplementary_data.zip › Supplementary Figure 7.docx]

**Supplementary Figure 7**


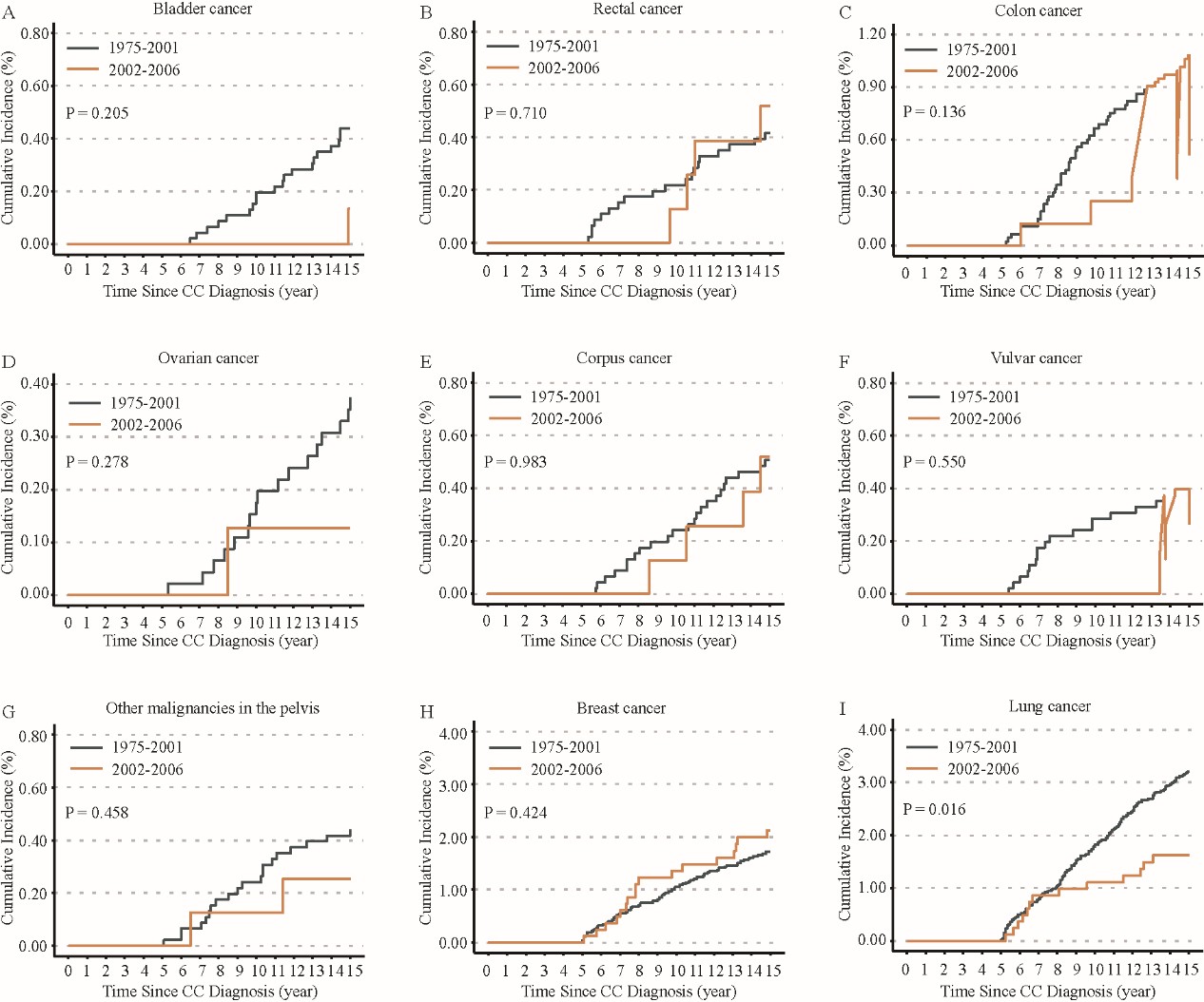


**Supplementary Figure 7.** Comparative Cumulative Incidence of Secondary Primary Malignancies (SPMs) 5–15 Years Post-EBRT in Cervical Cancer Patients Diagnosed Before and After 2001. This figure compares the cumulative incidence of SPMs within 15 years following external beam radiotherapy (EBRT) among cervical cancer patients diagnosed in two distinct eras: 1975–2001 and 2002–2006. (A) Bladder cancer. (B) Rectal cancer. (C) Colon cancer. (D) Ovarian cancer. (E) Uterine corpus cancer. (F) Vulvar cancer. (G) Other pelvic malignancies. (H) Breast cancer. (I) Lung cancer. Abbreviations: EBRT, external beam radiotherapy; SPMs, secondary primary malignancies.
