# Supplementary material for: The risk of radiation-associated second cancer in patients with cervical cancer following radiotherapy from 1975 to 2019
Source: Oncologist. 2025 Oct 10;30(11):oyaf334. doi: 10.1093/oncolo/oyaf334 (PMC12611298; doi:10.1093/oncolo/oyaf334)
Supplement: oyaf334_Supplementary_Data [file oyaf334_supplementary_data.zip › Supplementary Figure 9.docx]

**Supplementary Figure 9**


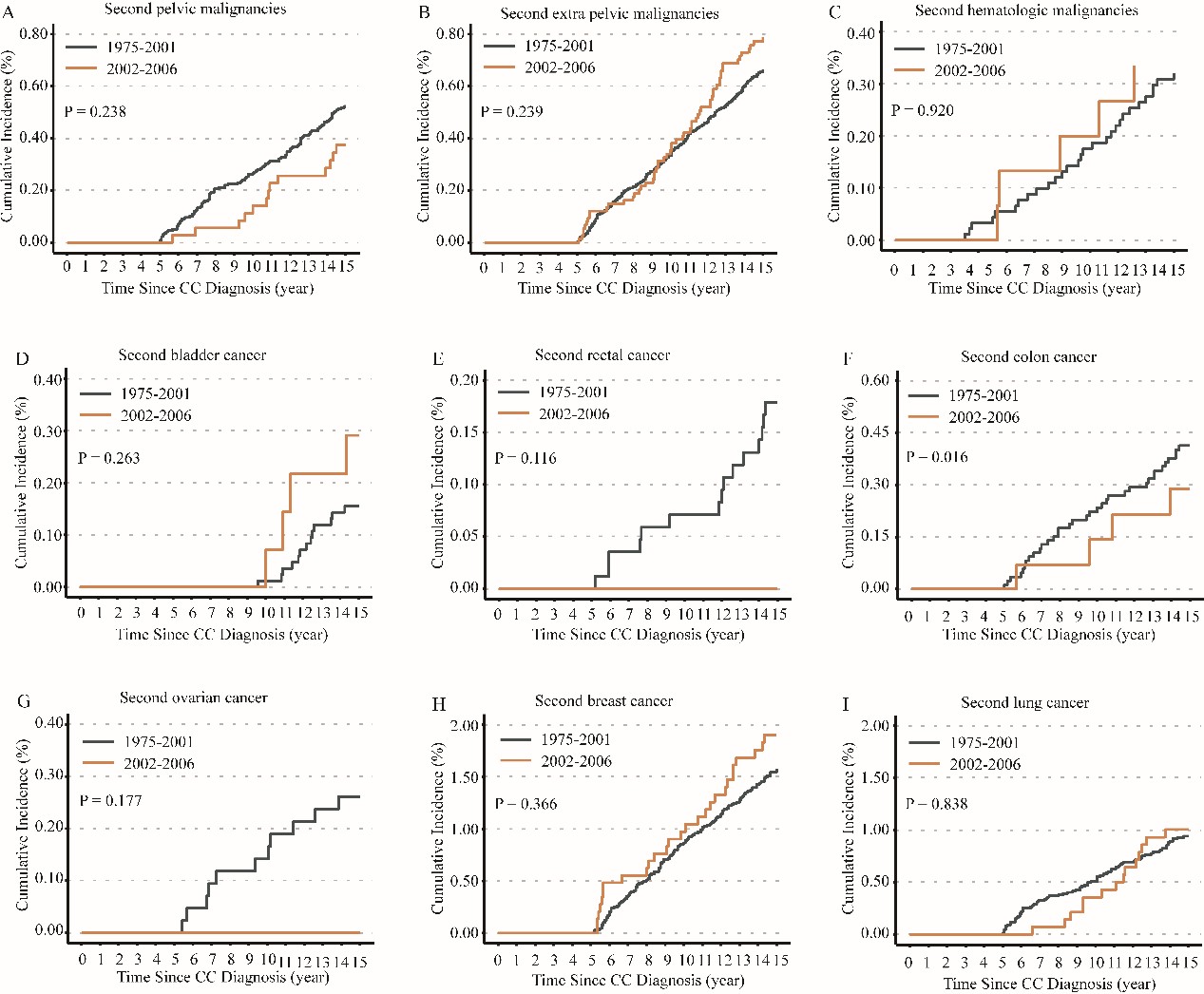


**Supplementary Figure 9.** Comparative 5-15 Years Cumulative Incidence of Secondary Primary Malignancies (SPMs) in Cervical Cancer Patients Not Treated with EBRT Diagnosed Before and After 2001. This figure presents the 15-year cumulative incidence of SPMs in cervical cancer patients who did not receive external beam radiotherapy (EBRT), comparing two diagnostic cohorts: 1975–2001 and 2002–2006. (A) Bladder cancer. (B) Rectal cancer. (C) Colon cancer. (D) Ovarian cancer. (E) Uterine corpus cancer. (F) Vulvar cancer. (G) Other pelvic malignancies. (H) Breast cancer. (I) Lung cancer. Abbreviations: EBRT, external beam radiotherapy; SPMs, secondary primary malignancies.
