# Supplementary material for: The risk of radiation-associated second cancer in patients with cervical cancer following radiotherapy from 1975 to 2019
Source: Oncologist. 2025 Oct 10;30(11):oyaf334. doi: 10.1093/oncolo/oyaf334 (PMC12611298; doi:10.1093/oncolo/oyaf334)
Supplement: oyaf334_Supplementary_Data [file oyaf334_supplementary_data.zip › Supplementary Figure 10.docx]

**Supplementary Figure 10**


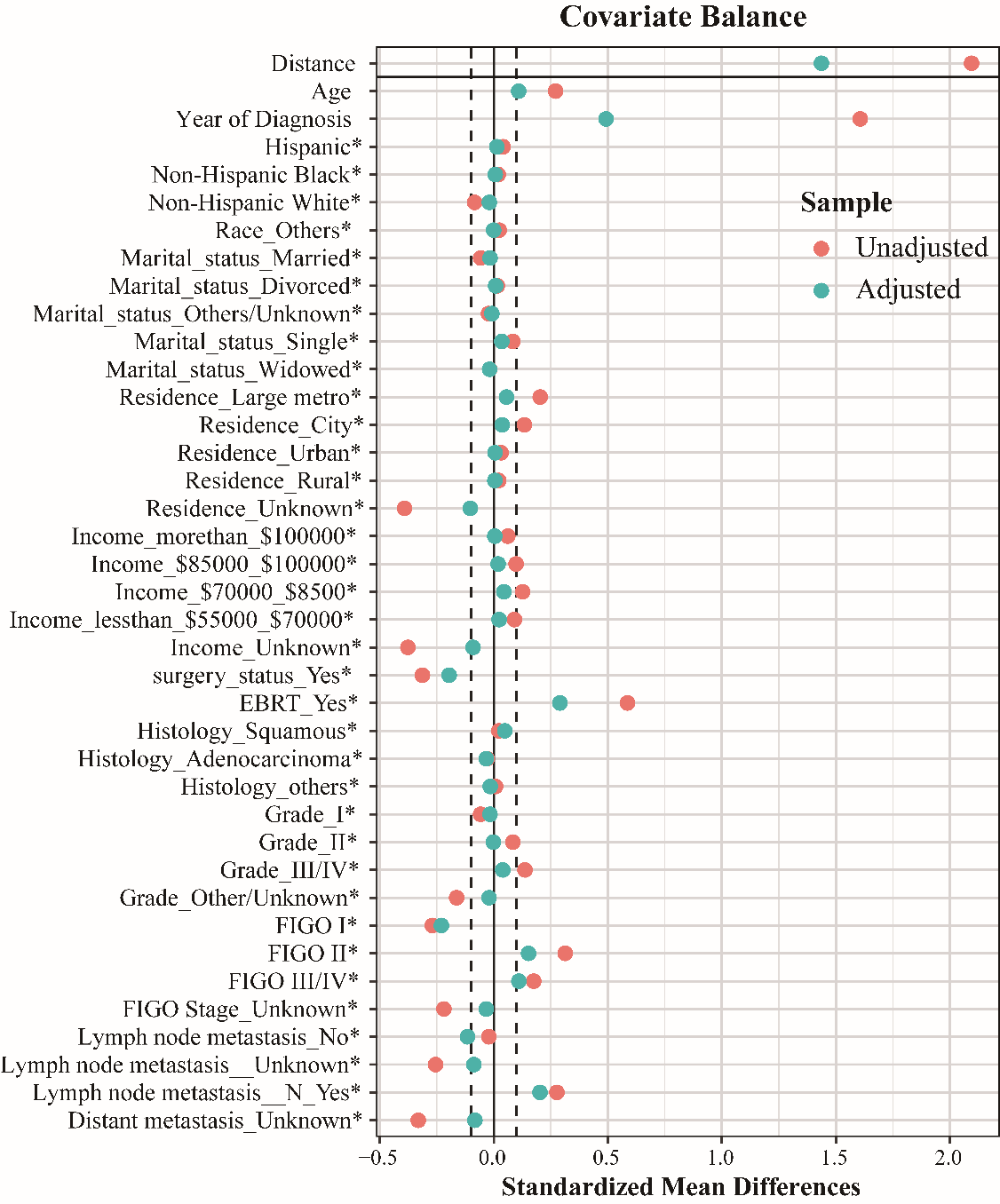


**Supplementary Figure 10.** Effectiveness of Propensity Score Matching (PSM) Evaluated Using a Love Plot for Chemotherapy and Non-Chemotherapy Groups. Red dots represent the standardized mean differences (SMDs) before matching, while green dots indicate the SMDs after matching, demonstrating improved covariate balance between the two groups.
