# Supplementary material for: The risk of radiation-associated second cancer in patients with cervical cancer following radiotherapy from 1975 to 2019
Source: Oncologist. 2025 Oct 10;30(11):oyaf334. doi: 10.1093/oncolo/oyaf334 (PMC12611298; doi:10.1093/oncolo/oyaf334)
Supplement: oyaf334_Supplementary_Data [file oyaf334_supplementary_data.zip › Supplementary Tables.docx]

Supplementary Tables

**The Risk of Radiation-Associated Second Cancer in Patients with Cervical Cancer Following Radiotherapy from 1975-2019**

[Supplementary Table 1. Definitions of Cancer Sites in the SEER 8-Based Analysis 3](file:///D:\BaiduSyncdisk\文献_paper\课题\在研课题\宫颈癌放疗相关第二原发肿瘤\投稿期刊\oncologist\返修文件用\返修文件夹\新建文件夹\Revised%20Supplementary%20Materials%20-%20副本%20-%20副本.docx#_Toc203922134)

[Supplementary Table 2. Baseline Characteristics After Propensity Score Matching Between EBRT and No/Unknown EBRT Groups (t-test or χ² test) 6](file:///D:\BaiduSyncdisk\文献_paper\课题\在研课题\宫颈癌放疗相关第二原发肿瘤\投稿期刊\oncologist\返修文件用\返修文件夹\新建文件夹\Revised%20Supplementary%20Materials%20-%20副本%20-%20副本.docx#_Toc203922135)

[Supplementary Table 3. Univariable And Multivariable Competing Risk Regression Analysis of The Risk of Developing Any Second Primary Malignancies (SPMs) In Cervical Cancer Patients 8](file:///D:\BaiduSyncdisk\文献_paper\课题\在研课题\宫颈癌放疗相关第二原发肿瘤\投稿期刊\oncologist\返修文件用\返修文件夹\新建文件夹\Revised%20Supplementary%20Materials%20-%20副本%20-%20副本.docx#_Toc203922136)

[Supplementary Table 4. Univariable and Multivariable Competing Risk Regression Analysis of the Risk of Developing Any Pelvic Second Primary Malignancies (SPMs) in Cervical Cancer Patients 9](file:///D:\BaiduSyncdisk\文献_paper\课题\在研课题\宫颈癌放疗相关第二原发肿瘤\投稿期刊\oncologist\返修文件用\返修文件夹\新建文件夹\Revised%20Supplementary%20Materials%20-%20副本%20-%20副本.docx#_Toc203922137)

[Supplementary Table 5. Univariable and Multivariable Competing Risk Regression Analysis of the Risk of Developing Any Extrapelvic Second Primary Malignancies (SPMs) in Cervical Cancer Patients 10](file:///D:\BaiduSyncdisk\文献_paper\课题\在研课题\宫颈癌放疗相关第二原发肿瘤\投稿期刊\oncologist\返修文件用\返修文件夹\新建文件夹\Revised%20Supplementary%20Materials%20-%20副本%20-%20副本.docx#_Toc203922138)

[Supplementary Table 6. Univariable and Multivariable Competing Risk Regression Analysis of the Risk of Developing Any Hematologic Second Primary Malignancies (SPMs) in Cervical Cancer Patients 11](file:///D:\BaiduSyncdisk\文献_paper\课题\在研课题\宫颈癌放疗相关第二原发肿瘤\投稿期刊\oncologist\返修文件用\返修文件夹\新建文件夹\Revised%20Supplementary%20Materials%20-%20副本%20-%20副本.docx#_Toc203922139)

[Supplementary Table 7. Univariable and Multivariable Competing Risk Regression Analyses of the Risk of Developing Any Second Primary Malignancies (SPMs) Among Cervical Cancer Patients After Propensity Score Matching (PSM)^(a)^ Between Radiotherapy and Non-Radiotherapy Groups 12](file:///D:\BaiduSyncdisk\文献_paper\课题\在研课题\宫颈癌放疗相关第二原发肿瘤\投稿期刊\oncologist\返修文件用\返修文件夹\新建文件夹\Revised%20Supplementary%20Materials%20-%20副本%20-%20副本.docx#_Toc203922140)

[Supplementary Table 8. Univariable and Multivariable Competing Risk Regression Analyses of the Risk of Developing Any Pelvic Second Primary Malignancies (SPMs) Among Cervical Cancer Patients After Propensity Score Matching (PSM)^(a)^ Between Radiotherapy and Non-Radiotherapy Groups 14](file:///D:\BaiduSyncdisk\文献_paper\课题\在研课题\宫颈癌放疗相关第二原发肿瘤\投稿期刊\oncologist\返修文件用\返修文件夹\新建文件夹\Revised%20Supplementary%20Materials%20-%20副本%20-%20副本.docx#_Toc203922141)

[Supplementary Table 9. Univariable and Multivariable Competing Risk Regression Analyses of the Risk of Developing Any Extrapelvic Second Primary Malignancies (SPMs) Among Cervical Cancer Patients After Propensity Score Matching (PSM)^(a)^ Between Radiotherapy and Non-Radiotherapy Groups 16](file:///D:\BaiduSyncdisk\文献_paper\课题\在研课题\宫颈癌放疗相关第二原发肿瘤\投稿期刊\oncologist\返修文件用\返修文件夹\新建文件夹\Revised%20Supplementary%20Materials%20-%20副本%20-%20副本.docx#_Toc203922142)

[Supplementary Table 10. Univariable and Multivariable Competing Risk Regression Analyses of the Risk of Developing Any Hematologic Second Primary Malignancies (SPMs) Among Cervical Cancer Patients After Propensity Score Matching (PSM)^(a)^ Between Radiotherapy and Non-Radiotherapy Groups 18](file:///D:\BaiduSyncdisk\文献_paper\课题\在研课题\宫颈癌放疗相关第二原发肿瘤\投稿期刊\oncologist\返修文件用\返修文件夹\新建文件夹\Revised%20Supplementary%20Materials%20-%20副本%20-%20副本.docx#_Toc203922143)

[Supplementary Table 11. Multivariable Competing Risk Analysis of the Cumulative Incidence of Second Primary Malignancies Among Cervical Cancer Patients According to Receipt of EBRT 20](file:///D:\BaiduSyncdisk\文献_paper\课题\在研课题\宫颈癌放疗相关第二原发肿瘤\投稿期刊\oncologist\返修文件用\返修文件夹\新建文件夹\Revised%20Supplementary%20Materials%20-%20副本%20-%20副本.docx#_Toc203922144)

[Supplementary Table 12. Comparison of 5 - to 15- Year Cumulative Incidence of Second Primary Malignancy (with Death as a Competing Event) in Cervical Cancer Patients after EBRT: A Comparison between Patients Diagnosed in 1975 -1994 and 1995 -2006 21](file:///D:\BaiduSyncdisk\文献_paper\课题\在研课题\宫颈癌放疗相关第二原发肿瘤\投稿期刊\oncologist\返修文件用\返修文件夹\新建文件夹\Revised%20Supplementary%20Materials%20-%20副本%20-%20副本.docx#_Toc203922145)

[Supplementary Table 13. Comparison of 5 - to 15- Year Cumulative Incidence of Second Primary Malignancy (with Death as a Competing Event) in Cervical Cancer Patients after EBRT: A Comparison between Patients Diagnosed in 1975 -2001 and 2002-2006 22](file:///D:\BaiduSyncdisk\文献_paper\课题\在研课题\宫颈癌放疗相关第二原发肿瘤\投稿期刊\oncologist\返修文件用\返修文件夹\新建文件夹\Revised%20Supplementary%20Materials%20-%20副本%20-%20副本.docx#_Toc203922146)

[Supplementary Table 14. The Influence of Brachytherapy on the Incidence of SPMs in Patients with Cervical Cancer 23](file:///D:\BaiduSyncdisk\文献_paper\课题\在研课题\宫颈癌放疗相关第二原发肿瘤\投稿期刊\oncologist\返修文件用\返修文件夹\新建文件夹\Revised%20Supplementary%20Materials%20-%20副本%20-%20副本.docx#_Toc203922147)

[Supplementary Table 15. Baseline Characteristics After Propensity Score Matching Between Chemotherapy and No/Unknown Chemotherapy Groups (t-test or χ² test) 24](file:///D:\BaiduSyncdisk\文献_paper\课题\在研课题\宫颈癌放疗相关第二原发肿瘤\投稿期刊\oncologist\返修文件用\返修文件夹\新建文件夹\Revised%20Supplementary%20Materials%20-%20副本%20-%20副本.docx#_Toc203922148)

[Supplementary Table 16. Univariable and Multivariable Competing Risk Regression Analyses of the Risk of Developing Any Second Primary Malignancies (SPMs) Among Cervical Cancer Patients After Propensity Score Matching (PSM)^(a)^ Between Chemotherapy and Non-Chemotherapy Groups 26](file:///D:\BaiduSyncdisk\文献_paper\课题\在研课题\宫颈癌放疗相关第二原发肿瘤\投稿期刊\oncologist\返修文件用\返修文件夹\新建文件夹\Revised%20Supplementary%20Materials%20-%20副本%20-%20副本.docx#_Toc203922149)

[Supplementary Table 17. Univariable and Multivariable Competing Risk Regression Analyses of the Risk of Developing Any Pelvic Second Primary Malignancies (SPMs) Among Cervical Cancer Patients After Propensity Score Matching (PSM)^(a)^ Between Chemotherapy and Non-Chemotherapy Groups 28](file:///D:\BaiduSyncdisk\文献_paper\课题\在研课题\宫颈癌放疗相关第二原发肿瘤\投稿期刊\oncologist\返修文件用\返修文件夹\新建文件夹\Revised%20Supplementary%20Materials%20-%20副本%20-%20副本.docx#_Toc203922150)

[Supplementary Table 18. Univariable and Multivariable Competing Risk Regression Analyses of the Risk of Developing Any Extrapelvic Second Primary Malignancies (SPMs) Among Cervical Cancer Patients After Propensity Score Matching (PSM)^(a)^ Between Chemotherapy and Non-Chemotherapy Groups 30](file:///D:\BaiduSyncdisk\文献_paper\课题\在研课题\宫颈癌放疗相关第二原发肿瘤\投稿期刊\oncologist\返修文件用\返修文件夹\新建文件夹\Revised%20Supplementary%20Materials%20-%20副本%20-%20副本.docx#_Toc203922151)

[Supplementary Table 19. Univariable and Multivariable Competing Risk Regression Analyses of the Risk of Developing Any Hematologic Second Primary Malignancies (SPMs) Among Cervical Cancer Patients After Propensity Score Matching (PSM)^(a)^ Between Chemotherapy and Non-Chemotherapy Groups 32](file:///D:\BaiduSyncdisk\文献_paper\课题\在研课题\宫颈癌放疗相关第二原发肿瘤\投稿期刊\oncologist\返修文件用\返修文件夹\新建文件夹\Revised%20Supplementary%20Materials%20-%20副本%20-%20副本.docx#_Toc203922152)

[Supplementary Table 20. Univariable Competing Risk Analysis of the Cumulative Incidence of Second Primary Malignancies Among Cervical Cancer Patients According to Receipt of Chemotherapy 34](file:///D:\BaiduSyncdisk\文献_paper\课题\在研课题\宫颈癌放疗相关第二原发肿瘤\投稿期刊\oncologist\返修文件用\返修文件夹\新建文件夹\Revised%20Supplementary%20Materials%20-%20副本%20-%20副本.docx#_Toc203922153)

[Supplementary Table 21. Multivariable Competing Risk Analysis of the Cumulative Incidence of Second Primary Malignancies Among Cervical Cancer Patients According to Receipt of Chemotherapy 35](file:///D:\BaiduSyncdisk\文献_paper\课题\在研课题\宫颈癌放疗相关第二原发肿瘤\投稿期刊\oncologist\返修文件用\返修文件夹\新建文件夹\Revised%20Supplementary%20Materials%20-%20副本%20-%20副本.docx#_Toc203922155)

[Supplementary Table 22. Comparison of 5- to 15-Year Cumulative Incidence of Second Primary Malignancies (Accounting for Death as a Competing Risk) Among Cervical Cancer Patients Not Treated with Chemotherapy: A Comparison between Patients Diagnosed in 1975 -1999 and 2000 -2006 36](file:///D:\BaiduSyncdisk\文献_paper\课题\在研课题\宫颈癌放疗相关第二原发肿瘤\投稿期刊\oncologist\返修文件用\返修文件夹\新建文件夹\Revised%20Supplementary%20Materials%20-%20副本%20-%20副本.docx#_Toc203922157)

Supplementary Table 1. Definitions of Cancer Sites in the SEER 8-Based Analysis

| **Hierarchy 1** | **Hierarchy 2** | **ICD-O-3 Site** | **ICD-O-3 Histology (Type)** |
| --- | --- | --- | --- |
| **All pelvic tumors** |  |  |  |
| **Urinary Bladder** | **-** | **C67.0-C67.9** | **8000, 8010, 8050, 8070, 8071, 8074, 8120, 8122, 8130, 8144,**  **8480** |
| **Rectum** | **-** | **C20.9** | **8000, 8010, 8070, 8140, 8210, 8240, 8261, 8263, 8480, 8481,**  **8490, 8890** |
| **Colon, NOS** | **-** | **C18.0, C18.2-C18.4, C18.6-C18.9, C19.9** | **8000, 8010, 8021, 8140, 8201, 8210, 8240, 8246, 8261, 8263,**  **8480, 8481, 8490, 8510, 8830** |
| **Small Intestine** | **-** | **C17.0-C17.2, C17.9** | **8010, 8140, 8240, 8249, 8805, 8890** |
| **Ovary** | **-** | **C56.9** | **8010, 8050, 8070, 8120, 8140, 8310, 8380, 8441, 8460, 8461,**  **8470, 8480, 8620, 8951, 9000** |
| **Corpus Uteri** | **-** | **C54.1, C54.2, C54.9** | **8010, 8032, 8083, 8140, 8255, 8260, 8310, 8323, 8380, 8441,**  **8460, 8560, 8800, 8950, 8980** |
| **Vagina** | **-** | **C52.9** | **8000, 8010, 8041, 8070, 8071, 8072, 8076, 8083, 8140, 8310,**  **8720, 8951, 9180** |
| **Vulva** | **-** | **C51.0-C51.2, C51.8-C51.9** | **8010, 8051, 8070, 8071, 8083, 8090, 8140, 8200, 8560, 8832** |
| **Anal Canal** | **-** | **C21.0-C21.2, C21.8** | **8010, 8020, 8051, 8070, 8071, 8072, 8083, 8124, 8140, 8246,**  **8720** |
| **Total of others** |  |  |  |
|  | **Fallopian tube** | **C57.0** | **8010, 8070, 8441, 8461** |
|  | **Female genital tract, NOS** | **C57.9** | **8010, 8140, 8441** |
|  | **Pelvic bones, sacrum, coccyx and associated joints** | **C41.4** | **8800, 8801, 8802, 9180, 9220** |
|  | **Pelvis, NOS** | **C76.3** | **8000, 8380** |
|  | **Uterus, NOS** | **C55.9** | **8010, 8140, 8460, 8800, 8935, 8950, 8980** |
|  | **Appendix** | **C18.1** | **8140, 8243, 8245, 9695** |
|  | **Conn, subcutaneous, other soft tis: lower limb, hip** | **C49.2** | **8805** |
|  | **Conn, subcutaneous, other soft tis: pelvis** | **C49.5** | **8070, 8800, 8801, 8810, 9370** |
|  | **Periph nerves & autonomic nerv sys: lower limb, hip** | **C47.2** | **9540** |
|  | **Periph nerves & autonomic nerv sys: pelvis** | **C47.5** | **8801** |
|  | **Skin of lower limb and hip** | **C44.7** | **8247, 8390, 8720, 8721, 8743, 8744, 9140, 9700** |
| **All extra pelvic tumors** |  |  |  |
| **Breast** | **-** | **C50.0-C50.6, C50.8-C50.9** | **8000, 8010, 8046, 8140, 8200, 8201, 8211, 8260, 8401, 8480,**  **8490, 8500, 8501, 8503, 8507, 8512, 8520, 8522, 8523, 8530,**  **8540, 8541, 8543, 8575** |
| **Lung and Bronchus** | **-** | **C34.0-C34.3, C34.8-C34.9** | **8000, 8010, 8012, 8013, 8041, 8042, 8043, 8044, 8046, 8070,**  **8071, 8072, 8073, 8083, 8123, 8140, 8230, 8240, 8246, 8249,**  **8250, 8253, 8260, 8480, 8550, 8551, 8560, 8800, 8980** |
| **Kidney and Renal Pelvis** | **-** | **C64.9, C65.9** | **8010, 8120, 8130, 8260, 8310, 8312, 8317** |
| **Pancreas** | **-** | **C25.0-C25.2,**  **C25.7-C25.9** | **8000, 8010, 8021, 8046, 8070, 8140, 8470, 8480, 8500, 8560** |
| **Thyroid** | **-** | **C73.9** | **8010, 8021, 8050, 8260, 8330, 8335, 8339, 8340, 8341, 8343,** |

|  |  |  | **8344** |
| --- | --- | --- | --- |
| **Stomach** | **-** | **C16.0, C16.2-C16.6,**  **C16.8-C16.9** | **8000, 8010, 8140, 8142, 8144, 8145, 8240, 8246, 8249, 8255,**  **8263, 8481, 8490, 8560, 8574** |
| **Melanoma** | **-** | **C44.2-C44.6** | **8720, 8721, 8742, 8743, 8772** |
| **Liver** | **-** | **C22.0** | **8140, 8160, 8170** |
| **Larynx** | **-** | **C32.0-C32.1, C32.9** | **8070, 8071** |
| **Brain** | **-** | **C71.1-C71.4, C71.6,**  **C71.8** | **9380, 9382, 9400, 9421, 9440** |
| **Esophagus** | **-** | **C15.3-C15.5, C15.8-C15.9** | **8070, 8071, 8072, 8140** |
| **Total of others** |  |  |  |
|  | **Accessory sinus, NOS** | **C31.9** | **8020** |
|  | **Adrenal gland, NOS** | **C74.9** | **8000** |
|  | **Ampulla of Vater** | **C24.1** | **8140, 8210** |
|  | **Anterior 2/3 of tongue, NOS** | **C02.3** | **8070, 8072** |
|  | **Base of tongue, NOS** | **C01.9** | **8070, 8072, 9690** |
|  | **Biliary tract, NOS** | **C24.9** | **8140** |
|  | **Cheek mucosa** | **C06.0** | **8070** |
|  | **Choroid** | **C69.3** | **8720, 9591** |
|  | **Conn, subcutaneous and other soft tissues, NOS** | **C49.9** | **9120** |
|  | **Conn, subcutaneous, other soft tis: abdomen** | **C49.4** | **8800, 8830, 8890, 9052** |
|  | **Conn, subcutaneous, other soft tis: upr limb, shoulder** | **C49.1** | **8800, 8840, 8854, 9560** |
|  | **External lower lip** | **C00.1** | **8070** |
|  | **External upper lip** | **C00.0** | **8070, 8071** |
|  | **Extrahepatic bile duct** | **C24.0** | **8160** |
|  | **Floor of mouth, NOS** | **C04.9** | **8070** |
|  | **Gallbladder** | **C23.9** | **8010, 8140, 8260, 8480, 8490** |
|  | **Gastrointestinal tract, NOS** | **C26.9** | **8140, 8246, 8481, 9699** |
|  | **Hypopharynx, NOS** | **C13.9** | **8070** |
|  | **Intrahepatic bile duct** | **C22.1** | **8140, 8160** |
|  | **Lateral floor of mouth** | **C04.1** | **8070** |
|  | **Lower gum** | **C03.1** | **8070, 8071** |
|  | **Mouth, NOS** | **C06.9** | **8070** |
|  | **Nasal cavity** | **C30.0** | **8010, 8070, 8071, 9590** |
|  | **Nasopharynx, NOS** | **C11.9** | **8070** |
|  | **Oropharynx, NOS** | **C10.9** | **8010, 8070, 8071** |
|  | **Overlap conn, subcutaneous, and other soft tissues** | **C49.8** | **9120** |
|  | **Overlapping lesion of floor of mouth** | **C04.8** | **8071** |
|  | **Overlapping lesion of lip, oral cavity & pharynx** | **C14.8** | **8070, 9680** |
|  | **Parotid gland** | **C07.9** | **8070, 9698** |
|  | **Peritoneum, NOS** | **C48.2** | **8010, 8310, 8441, 8461, 8900, 9052** |
|  | **Pleura, NOS** | **C38.4** | **9051** |
|  | **Retromolar area** | **C06.2** | **8070, 8430** |
|  | **Retroperitoneum** | **C48.0** | **8010, 8830, 8851, 9240, 9665** |

|  | **Skin of trunk** | **C44.5** | **8247, 8410, 8720, 8743, 9120, 9700** |
| --- | --- | --- | --- |
|  | **Soft palate, NOS** | **C05.1** | **8070** |
|  | **Specified parts of peritoneum** | **C48.1** | **8140, 8441, 9680** |
|  | **Spinal cord** | **C72.0** | **9391** |
|  | **Splenic flexure of colon** | **C18.5** | **8140** |
|  | **Sublingual gland** | **C08.1** | **8200** |
|  | **Thymus** | **C37.9** | **8586** |
|  | **Tonsil, NOS** | **C09.9** | **8070, 8072, 9695** |
|  | **Tonsillar fossa** | **C09.0** | **8070** |
|  | **Upper gum** | **C03.0** | **8070** |
|  | **Ureter** | **C66.9** | **8120** |
|  | **Urethra** | **C68.0** | **8130, 8140** |
|  | **Urinary system, NOS** | **C68.9** | **8001, 8120** |
|  | **Vallecula** | **C10.0** | **8070** |
|  | **Ventral surface of tongue, NOS** | **C02.2** | **8070** |
| **All hematologic malignancies** |  |  |  |
| **Lymphoma** |  |  |  |
|  | **Hodgkin-Extra nodal** | **C48.0** | **9665** |
|  | **Hodgkin-Nodal** | **C77.0, C77.8-C77.9** | **9650, 9652, 9653, 9663** |
|  | **Non-Hodgkin-Extra nodal** | **C01.9, C07.9, C08.0, C14.8, C16.0- C16.3,**  **C16.9, C17.0, C17.2, C17.9, C18.0, C18.1, C22.0, C26.9, C30.0, C34.1,**  **C34.9, C38.0, C38.3, C41.2, C42.1, C44.3, C44.5, C44.7, C44.9, C48.1,**  **C50.3, C50.8, C64.9, C69.0, C69.3, C69.6, C71.1, C71.8, C71.9** | **9590, 9591, 9671, 9673, 9679, 9680, 9684, 9690, 9691, 9695,**  **9698, 9699, 9700, 9823** |
|  | **Non-Hodgkin-Nodal** | **C09.9, C42.2, C77.0, C77.2- C77.4,**  **C77.8- C77.9** | **9590, 9591, 9670, 9671, 9673, 9675, 9680, 9684, 9689, 9690,**  **9695, 9698, 9699, 9705, 9714, 9823** |
| **Lymphocytic leukemia** |  |  |  |
|  | **Acute Lymphocytic Leukemia** | **C42.1** | **9811, 9835, 9836** |
|  | **Chronic Lymphocytic Leukemia** | **C42.1** | **9823** |
|  | **Other Lymphocytic Leukemia** | **C42.1** | **9820, 9940** |
| **Nonlymphocytic leukemia** |  |  |  |
|  | **Acute Monocytic Leukemia** | **C42.1** | **9891** |
|  | **Acute Myeloid Leukemia** | **C42.1** | **9861, 9867, 9871, 9895, 9897, 9920** |
|  | **Chronic Myeloid Leukemia** | **C42.1** | **9863, 9875, 9945** |
|  | **Other Acute Leukemia** | **C42.1** | **9801** |
|  | **Other Myeloid/Monocytic Leukemia** | **C42.1** | **9860** |
| **Myeloma** | **-** | **C42.1** | **9732** |
| **Total of others** |  |  |  |
|  | **Aleukemic, Sub leukemic and NOS** | **C42.1** | **9831** |
|  | **Miscellaneous** | **C42.0-C42.1, C76.0, C76.3. C80.9** | **8000, 8010, 8012, 8013, 8022, 8041, 8046, 8070, 8071, 8140,**  **8246, 8380, 8481, 8560, 9043, 9761, 9950, 9961, 9962, 9980,**  **9983, 9985, 9986, 9989** |

Supplementary Table 2. Baseline Characteristics After Propensity Score Matching Between EBRT and No/Unknown EBRT Groups (t-test or χ² test)

| **Characteristics** | **EBRT,**  **No. (%)** | **No or Unknown**  **EBRT, No. (%)** | ***P*-value** | **SMD** |
| --- | --- | --- | --- | --- |
| Total | 11503 | 11503 | - | - |
| Age at CC diagnosis, mean (SD), y | 52.53 (14.4) | 45.24 (13.9) | <0.001 | 0.517 |
| Years of CC diagnosis, mean | 1996 | 1993 | <0.001 | 0.227 |
| Race |  |  | <0.001 | 0.103 |
| Hispanic | 1507 (13.1) | 1312 (11.4) |  |  |
| Non-Hispanic Black | 1255 (10.9) | 1076 (9.4) |  |  |
| Non-Hispanic White | 7160 (62.2) | 7727 (67.2) |  |  |
| Others | 1581 (13.7) | 1388 (12.1) |  |  |
| Marital status |  |  | <0.001 | 0.242 |
| Married | 5539 (48.2) | 6410 (55.7) |  |  |
| Divorced | 1586 (13.8) | 1597 (13.9) |  |  |
| Single | 1856 (16.1) | 1774 (15.4) |  |  |
| Widowed | 1758 (15.3) | 914 (7.9) |  |  |
| Unknown | 764 (6.6) | 808 (7.0) |  |  |
| Residence |  |  | <0.001 | 0.123 |
| Large metro | 3685 (32.0) | 3489 (30.3) |  |  |
| City | 2241 (19.5) | 1911 (16.6) |  |  |
| Urban | 566 (4.9) | 476 (4.1) |  |  |
| Rural | 495 (4.3) | 450 (3.9) |  |  |
| Unknown | 4516 (39.3) | 5177 (45.0) |  |  |
| Income |  |  | <0.001 | 0.109 |
| > $100000 | 1165 (10.1) | 1108 (9.6) |  |  |
| $85000-$100000 | 2245 (19.5) | 2211 (19.2) |  |  |
| $70000-$8500 | 2072 (18.0) | 1850 (16.1) |  |  |
| < $55000-$70000 | 1643 (14.3) | 1396 (12.1) |  |  |
| Unknown | 4378 (38.1) | 4938 (42.9) |  |  |
| FIGO staging (Version 2009) |  |  | <0.001 | 0.959 |
| I | 4117 (35.8) | 7833 (68.1) |  |  |
| II | 3171 (27.6) | 442 (3.8) |  |  |
| III/ IV | 1410 (12.3) | 128 (1.1) |  |  |
| Unknown | 2805 (24.4) | 3100 (26.9) |  |  |
| Lymph node metastases |  |  | <0.001 | 0.546 |
| Yes | 1968 (17.1) | 247 (2.1) |  |  |
| No | 5352 (46.5) | 7140 (62.1) |  |  |
| Unknown | 4183 (36.4) | 4116 (35.8) |  |  |
| Distant metastases |  |  | <0.001 | 0.084 |
| M0 | 7421 (64.5) | 6954 (60.5) |  |  |
| Unknown | 4082 (35.5) | 4549 (39.5) |  |  |
| Histological type |  |  | <0.001 | 0.19 |

| Squamous cell carcinoma | 8779 (76.3) | 7809 (67.9) |  |
| --- | --- | --- | --- |
| Adenocarcinoma | 1302 (11.3) | 1850 (16.1) |  |
| Other/Unknown | 1422 (12.4) | 1844 (16.0) |  |
| Grade |  | <0.001 | 0.339 |
| I | 738 (6.4) | 1217 (10.6) |  |
| II | 3371 (29.3) | 2957 (25.7) |  |
| III/IV | 3587 (31.2) | 2238 (19.5) |  |
| Other/Unknown | 3807 (33.1) | 5091 (44.3) |  |
| Surgery |  | <0.001 | 1.075 |
| Yes | 5037 (43.8) | 10189 (88.6) |  |
| No/ Unknown | 6466 (56.2) | 1314 (11.4) |  |
| Chemotherapy |  | <0.001 | 0.932 |
| Yes | 4582 (39.8) | 540 (4.7) |  |
| No/Unknown | 6921 (60.2) | 10963 (95.3) |  |

Abbreviations: EBRT, external beam radiotherapy; SMD, standardized mean difference.

Supplementary Table 3. Univariable And Multivariable Competing Risk Regression Analysis of The Risk of Developing Any Second Primary Malignancies (SPMs) In Cervical Cancer Patients

|  | **Univariable Competing Risk Regression** | | **Multivariable Competing Risk Regression** | |
| --- | --- | --- | --- | --- |
| **Characteristics** | **sHR (95% CI)** | ***P* value** | **sHR (95% CI)** | ***P* value** |
| **Age at CC diagnosis, y** | 1.01 (1.01-1.01) | < 0.001 | 1.01 (1.01-1.02) | < 0.001 |
| **Years of CC diagnosis** | 0.98 (0.98-0.99) | < 0.001 | 0.98 (0.97-0.99) | < 0.001 |
| **Race** |  |  |  |  |
| Hispanic | Reference |  | Reference |  |
| Non-Hispanic Black | 1.23 (1.03-1.46) | 0.019 | 1.22 (1.02-1.45) | 0.027 |
| Non-Hispanic White | 1.18 (1.03-1.35) | 0.016 | 1.12 (0.98-1.28) | 0.11 |
| Others | 0.72 (0.5-1.04) | 0.082 | 0.72 (0.5-1.05) | 0.087 |
| **Marital status** |  |  |  |  |
| Married | Reference |  | Reference |  |
| Divorced | 0.98 (0.88-1.1) | 0.77 | 0.97 (0.87-1.09) | 0.63 |
| Single | 0.73 (0.65-0.83) | 0.16 | 0.86 (0.76-0.97) | 0.015 |
| Widowed | 0.9 (0.79-1.03) | 0.13 | 0.66 (0.57-0.76) | < 0.001 |
| Unknown | 0.93 (0.8-1.07) | 0.3 | 0.95 (0.82-1.1) | 0.51 |
| **Residence** |  |  | 1.06 (0.94-1.2) | 0.330 |
| Large metro | Reference |  | Reference |  |
| City | 1.03 (0.9-1.17) | 0.67 | 1.09 (0.94-1.25) | 0.25 |
| Urban | 1.03 (0.9-1.17) | 0.38 | 1.16 (0.9-1.5) | 0.25 |
| Rural | 1.17 (0.95-1.44) | 0.14 | 1.26 (0.95-1.67) | 0.11 |
| Unknown | 1.31 (1.19-1.44) | < 0.001 | 0.7 (0.5-0.99) | 0.047 |
| **Income** |  |  |  |  |
| > $100000 | Reference |  | Reference |  |
| $85000-$100000 | 1.22 (1.02-1.46) | 0.003 | 1.26 (1.05-1.51) | 0.014 |
| $70000-$8500 | 1.16 (0.97-1.4) | 0.110 | 1.14 (0.95-1.38) | 0.17 |
| < $55000-$70000 | 1.18 (0.97-1.43) | 0.093 | 1.07 (0.83-1.36) | 0.61 |
| Unknown | 1.51 (1.28-1.77) | < 0.001 | 1.72 (1.16-2.56) | 0.008 |
| **FIGO staging** |  |  |  |  |
| I | Reference |  | Reference |  |
| II | 1.16 (1.03-1.31) | 0.013 | 1 (0.87-1.15) | 0.96 |
| III/ IV | 0.88 (0.72-1.07) | 0.2 | 0.75 (0.6-0.93) | 0.009 |
| Unknown | 1.19 (1.1-1.3) | < 0.001 | 0.91 (0.77-1.07) | 0.24 |
| **Lymph node metastases** |  |  |  |  |
| No | Reference |  | Reference |  |
| Yes | 0.94 (0.79-1.12) | 0.5 | 0.9 (0.75-1.08) | 0.25 |
| Unknown | 1.13 (1.05-1.22) | 0.002 | 0.95 (0.84-1.08) | 0.46 |
| **Distant metastases** |  |  |  |  |
| M0 | Reference |  | Reference |  |
| Unknown | 1.24 (1.15-1.34) | < 0.001 | 1 (0.84-1.17) | 0.96 |
| **Histological type** |  |  |  |  |
| Squamous cell carcinoma | Reference |  | Reference |  |
| Adenocarcinoma | 0.91 (0.81-1.02) | 0.11 | 0.96 (0.84-1.08) | 0.48 |
| Other/Unknown | 0.82 (0.74-0.92) | < 0.001 | 0.84 (0.76-0.94) | 0.003 |
| **Grade** |  |  |  |  |
| I | Reference |  | Reference |  |
| II | 1.03 (0.89-1.2) | 0.68 | 0.99 (0.85-1.16) | 0.92 |
| III/IV | 1.13 (0.97-1.31) | 0.12 | 1.06 (0.9-1.24) | 0.49 |
| Other/Unknown | 1.04 (0.9-1.2) | 0.59 | 1.02 (0.88-1.19) | 0.78 |
| **Surgery** |  |  |  |  |
| No/ Unknown | Reference |  | Reference |  |
| Yes | 0.89 (0.82-0.96) | 0.004 | 1.12 (1.01-1.25) | 0.04 |
| **Chemotherapy** |  |  |  |  |
| No/Unknown | Reference |  | Reference |  |
| Yes | 0.92 (0.82-1.04) | 0.170 | 1.1 (0.94-1.29) | 0.23 |
| **External beam radiotherapy** |  |  |  |  |
| No/Unknown | Reference |  | Reference |  |
| Yes | 1.28 (1.18-1.38) | < 0.001 | 1.2 (1.08-1.33) | < 0.001 |

Supplementary Table 4. Univariable and Multivariable Competing Risk Regression Analysis of the Risk of Developing Any Pelvic Second Primary Malignancies (SPMs) in Cervical Cancer Patients

|  | **Univariable Competing Risk Regression** | | **Multivariable Competing Risk Regression** | |
| --- | --- | --- | --- | --- |
| **Characteristics** | **sHR (95% CI)** | ***P* value** | **sHR (95% CI)** | ***P* value** |
| **Age at CC diagnosis-y** | 1.02 (1.02-1.03) | < 0.001 | 1.01 (1.01-1.02) | < 0.001 |
| **Years of CC diagnosis** | 0.98 (0.97-0.99) | < 0.001 | 0.99 (0.97-1.01) | 0.21 |
| **Race** |  |  |  |  |
| Hispanic | Reference |  | Reference |  |
| Non-Hispanic Black | 1.35 (1-1.84) | 0.051 | 1.38 (1.01-1.89) | 0.044 |
| Non-Hispanic White | 1.05 (0.83-1.34) | 0.68 | 1.09 (0.85-1.39) | 0.5 |
| Others | 0.78 (0.41-1.46) | 0.43 | 0.83 (0.44-1.55) | 0.55 |
| **Marital status** |  |  |  |  |
| Married | Reference |  | Reference |  |
| Divorced | 1.09 (0.89-1.34) | 0.41 | 1.03 (0.84-1.27) | 0.77 |
| Single | 0.73 (0.58-0.91) | 0.06 | 0.87 (0.69-1.09) | 0.23 |
| Widowed | 1.09 (0.86-1.38) | 0.48 | 0.62 (0.48-0.81) | 0.001 |
| Unknown | 0.82 (0.62-1.1) | 0.19 | 0.79 (0.59-1.06) | 0.12 |
| **Residence** |  |  |  |  |
| Large metro | Reference |  | Reference |  |
| City | 1.06 (0.83-1.36) | 0.62 | 1.1 (0.82-1.46) | 0.53 |
| Urban | 1.35 (0.93-1.98) | 0.12 | 1.18 (0.72-1.93) | 0.51 |
| Rural | 1.02 (0.66-1.58) | 0.93 | 0.92 (0.51-1.65) | 0.77 |
| Unknown | 1.47 (1.23-1.77) | < 0.001 | 0.84 (0.45-1.58) | 0.6 |
| **Income** |  |  |  |  |
| > $100000 | Reference |  | Reference |  |
| $85000-$100000 | 1.56 (1.08-2.24) | 0.017 | 1.55 (1.07-2.24) | 0.021 |
| $70000-$8500 | 1.27 (0.87-1.87) | 0.220 | 1.18 (0.8-1.76) | 0.4 |
| < $55000-$70000 | 1.65 (1.12-2.43) | 0.011 | 1.52 (0.93-2.49) | 0.094 |
| Unknown | 2.02 (1.45-2.81) | < 0.001 | 1.62 (0.76-3.45) | 0.21 |
| **FIGO staging** |  |  |  |  |
| I | Reference |  | Reference |  |
| II | 2.14 (1.73-2.64) | < 0.001 | 1.13 (0.88-1.45) | 0.33 |
| III/ IV | 1.69 (1.19-2.39) | 0.003 | 0.8 (0.54-1.19) | 0.27 |
| Unknown | 1.71 (1.46-2) | < 0.001 | 0.97 (0.72-1.32) | 0.86 |
| **Lymph node metastases** |  |  |  |  |
| No | Reference |  | Reference |  |
| Yes | 1.33 (0.96-1.84) | 0.009 | 0.92 (0.65-1.31) | 0.65 |
| Unknown | 1.53 (1.33-1.78) | < 0.001 | 1.14 (0.91-1.44) | 0.26 |
| **Distant metastases** |  |  |  |  |
| M0 | Reference |  | Reference |  |
| Unknown | 1.45 (1.26-1.67) | < 0.001 | 1.1 (0.8-1.52) | 0.55 |
| **Histological type** |  |  |  |  |
| Squamous cell carcinoma | Reference |  | Reference |  |
| Adenocarcinoma | 0.81 (0.64-1.01) | 0.063 | 0.99 (0.78-1.26) | 0.92 |
| Other/Unknown | 0.73 (0.59-0.91) | 0.004 | 0.84 (0.67-1.04) | 0.11 |
| **Grade** |  |  |  |  |
| I | Reference |  | Reference |  |
| II | 1.18 (0.89-1.57) | 0.26 | 1 (0.74-1.34) | 0.98 |
| III/IV | 1.3 (0.98-1.73) | 0.07 | 1.02 (0.76-1.37) | 0.89 |
| Other/Unknown | 1.04 (0.79-1.35) | 0.79 | 1.03 (0.78-1.36) | 0.83 |
| **Surgery** |  |  |  |  |
| No/ Unknown | Reference |  | Reference |  |
| Yes | 0.45 (0.39-0.52) | < 0.001 | 0.82 (0.68-1) | 0.051 |
| **Chemotherapy** |  |  |  |  |
| No/Unknown | Reference |  | Reference |  |
| Yes | 1.41 (1.14-1.74) | 0.001 | 1.13 (0.83-1.52) | 0.44 |
| **External beam radiotherapy** |  |  |  |  |
| No/Unknown | Reference |  | Reference |  |
| Yes | 2.13 (1.81-2.50) | < 0.001 | 2.01 (1.64-2.46) | < 0.001 |

Supplementary Table 5. Univariable and Multivariable Competing Risk Regression Analysis of the Risk of Developing Any Extrapelvic Second Primary Malignancies (SPMs) in Cervical Cancer Patients

|  | **Univariable Competing Risk Regression** | | **Multivariable Competing Risk Regression** | |
| --- | --- | --- | --- | --- |
| **Characteristics** | **sHR (95% CI)** | ***P* value** | **sHR (95% CI)** | ***P* value** |
| **Age at CC diagnosis, y** | 1.02 (1.01-1.02) | < 0.001 | 1.02 (1.01, 1.02) | < 0.001 |
| **Years of CC diagnosis** | 0.99 (0.99-1.0) | < 0.001 | 0.99 (0.98, 1) | 0.079 |
| **Race** |  |  |  |  |
| Hispanic | Reference |  | Reference |  |
| Non-Hispanic Black | 1.4 (1.11, 1.77) | 0.005 | 1.36 (1.08, 1.72) | 0.01 |
| Non-Hispanic White | 1.3 (1.08, 1.57) | 0.005 | 1.24 (1.03, 1.49) | 0.024 |
| Others | 0.68 (0.4, 1.15) | 0.150 | 0.69 (0.41, 1.16) | 0.16 |
| **Marital status** |  |  |  |  |
| Married | Reference |  | Reference |  |
| Divorced | 0.97 (0.84, 1.13) | 0.71 | 0.95 (0.82, 1.1) | 0.5 |
| Single | 0.76 (0.65, 0.88) | < 0.001 | 0.89 (0.76, 1.05) | 0.16 |
| Widowed | 1.01 (0.85, 1.2) | 0.88 | 0.69 (0.57, 0.83) | < 0.001 |
| Unknown | 0.98 (0.81, 1.18) | 0.80 | 1 (0.83, 1.21) | 1 |
| **Residence** |  |  |  |  |
| Large metro | Reference |  | Reference |  |
| City | 1.09 (0.92, 1.29) | 0.31 | 1.18 (0.99, 1.41) | 0.071 |
| Urban | 1.08 (0.81, 1.44) | 0.59 | 1.28 (0.92, 1.77) | 0.14 |
| Rural | 1.23 (0.94, 1.61) | 0.14 | 1.54 (1.07, 2.2) | 0.019 |
| Unknown | 1.28 (1.13, 1.45) | < 0.001 | 0.84 (0.55, 1.3) | 0.44 |
| **Income** |  |  |  |  |
| > $100000 | Reference |  | Reference |  |
| $85000-$100000 | 1.2 (0.95, 1.51) | 0.13 | 1.2 (0.95, 1.52) | 0.13 |
| $70000-$8500 | 1.26 (0.99, 1.59) | 0.059 | 1.21 (0.95, 1.54) | 0.13 |
| < $55000-$70000 | 1.1 (0.85, 1.42) | 0.46 | 0.91 (0.66, 1.26) | 0.58 |
| Unknown | 1.43 (1.16, 1.76) | 0.001 | 1.6 (0.97, 2.65) | 0.067 |
| **FIGO staging** |  |  |  |  |
| I | Reference |  | Reference |  |
| II | 1.22 (1.03, 1.44) | 0.002 | 1.04 (0.86, 1.26) | 0.66 |
| III/ IV | 1.07 (0.81, 1.4) | 0.63 | 0.89 (0.66, 1.2) | 0.43 |
| Unknown | 1.16 (1.04, 1.29) | 0.001 | 0.94 (0.76, 1.17) | 0.58 |
| **Lymph node metastases** |  |  |  |  |
| No | Reference |  | Reference |  |
| Yes | 1.06 (0.84, 1.35) | 0.61 | 1 (0.77, 1.29) | 0.99 |
| Unknown | 1.1 (0.99, 1.22) | 0.064 | 0.98 (0.83, 1.16) | 0.78 |
| **Distant metastases** |  |  |  |  |
| M0 | Reference |  | Reference |  |
| Unknown | 1.19 (1.08, 1.32) | 0.001 | 1.04 (0.84, 1.28) | 0.75 |
| **Histological type** |  |  |  |  |
| Squamous cell carcinoma | Reference |  | Reference |  |
| Adenocarcinoma | 0.93 (0.8, 1.08) | 0.33 | 0.99 (0.84, 1.17) | 0.89 |
| Other/Unknown | 0.84 (0.73, 0.97) | 0.019 | 0.86 (0.74, 0.99) | 0.038 |
| **Grade** |  |  |  |  |
| I | Reference |  | Reference |  |
| II | 1.04 (0.85, 1.27) | 0.70 | 1 (0.82, 1.23) | 0.99 |
| III/IV | 1.21 (0.99, 1.47) | 0.061 | 1.15 (0.93, 1.41) | 0.2 |
| Other/Unknown | 1.05 (0.87, 1.26) | 0.60 | 1.06 (0.87, 1.28) | 0.58 |
| **Surgery** |  |  |  |  |
| No/ Unknown | Reference |  | Reference |  |
| Yes | 0.87 (0.78, 0.98) | 0.018 | 1.13 (0.97, 1.31) | 0.12 |
| **Chemotherapy** |  |  |  |  |
| No/Unknown | Reference |  | Reference |  |
| Yes | 1.04 (0.88, 1.22) | 0.650 | 1.11 (0.9, 1.37) | 0.34 |
| **External beam radiotherapy** |  |  |  |  |
| No/Unknown | Reference |  | Reference |  |
| Yes | 1.03 (0.92-1.15) | 0.59 | 1.08 (0.94, 1.24) | 0.27 |

Supplementary Table 6. Univariable and Multivariable Competing Risk Regression Analysis of the Risk of Developing Any Hematologic Second Primary Malignancies (SPMs) in Cervical Cancer Patients

|  | **Univariable Competing Risk Regression** | | **Multivariable Competing Risk Regression** | |
| --- | --- | --- | --- | --- |
| **Characteristics** | **sHR (95% CI)** | ***P* value** | **sHR (95% CI)** | ***P* value** |
| **Age at CC diagnosis-y** | 1.02 (1.01-1.03) | < 0.001 | 1.03 (1.02, 1.04) | < 0.001 |
| **Years of CC diagnosis** | 0.99 (0.98-1.00) | 0.17 | 0.97 (0.94, 0.99) | 0.01 |
| **Race** |  |  |  |  |
| Hispanic | Reference |  | Reference |  |
| Non-Hispanic Black | 0.82 (0.44-1.5) | 0.51 | 0.79 (0.44, 1.45) | 0.45 |
| Non-Hispanic White | 1.17 (0.77-1.79) | 0.47 | 1.12 (0.73, 1.74) | 0.6 |
| Others | 0.69 (0.21-2.27) | 0.54 | 0.72 (0.22, 2.4) | 0.59 |
| **Marital status** |  |  |  |  |
| Married | Reference |  | Reference |  |
| Divorced | 1 (0.69-1.46) | 1.0 | 0.95 (0.65, 1.4) | 0.81 |
| Single | 0.64 (0.42-0.97) | 0.035 | 0.77 (0.5, 1.18) | 0.23 |
| Widowed | 0.88 (0.57-1.37) | 0.58 | 0.56 (0.34, 0.91) | 0.021 |
| Unknown | 1.1 (0.7-1.72) | 0.69 | 1.22 (0.76, 1.94) | 0.41 |
| **Residence** |  |  |  |  |
| Large metro | Reference |  | Reference |  |
| City | 0.7 (0.46-1.09) | 0.11 | 0.62 (0.38, 1) | 0.049 |
| Urban | 0.91 (0.45-1.82) | 0.78 | 0.62 (0.26, 1.49) | 0.28 |
| Rural | 1.46 (0.82-2.6) | 0.2 | 0.89 (0.38, 2.08) | 0.79 |
| Unknown | 1.04 (0.77-1.39) | 0.82 | 0.26 (0.04, 1.87) | 0.18 |
| **Income** |  |  |  |  |
| > $100000 | Reference |  | Reference |  |
| $85000-$100000 | 0.79 (0.47-1.33) | 0.38 | 0.88 (0.52, 1.49) | 0.62 |
| $70000-$8500 | 0.65 (0.37-1.14) | 0.13 | 0.73 (0.41, 1.3) | 0.29 |
| < $55000-$70000 | 1.09 (0.64-1.85) | 0.76 | 1.29 (0.62, 2.71) | 0.49 |
| Unknown | 1 (0.65-1.56) | 0.98 | 3.93 (0.51, 30.34) | 0.19 |
| **FIGO staging** |  |  |  |  |
| I | Reference |  | Reference |  |
| II | 1.35 (0.94-1.95) | 0.11 | 1.31 (0.85, 2) | 0.22 |
| III/ IV | 0.84 (0.44-1.59) | 0.58 | 0.77 (0.37, 1.59) | 0.48 |
| Unknown | 0.88 (0.65-1.2) | 0.41 | 0.99 (0.57, 1.74) | 0.98 |
| **Lymph node metastases** |  |  |  |  |
| No | Reference |  | Reference |  |
| Yes | 1.22 (0.77-1.95) | 0.4 | 1.26 (0.73, 2.17) | 0.41 |
| Unknown | 0.75 (0.57-0.99) | 0.43 | 0.58 (0.37, 0.93) | 0.024 |
| **Distant metastases** |  |  |  |  |
| M0 | Reference |  | Reference |  |
| Unknown | 0.99 (0.76, 1.27) | 0.91 | 0.73 (0.43, 1.25) | 0.25 |
| **Histological type** |  |  |  |  |
| Squamous cell carcinoma | Reference |  | Reference |  |
| Adenocarcinoma | 1.02 (0.7, 1.49) | 0.92 | 1.07 (0.72, 1.59) | 0.73 |
| Other/Unknown | 0.8 (0.54, 1.17) | 0.24 | 0.82 (0.56, 1.2) | 0.3 |
| **Grade** |  |  |  |  |
| I | Reference |  | Reference |  |
| II | 1.32 (0.77, 2.27) | 0.31 | 1.27 (0.72, 2.22) | 0.41 |
| III/IV | 1.47 (0.86, 2.52) | 0.16 | 1.38 (0.79, 2.43) | 0.26 |
| Other/Unknown | 1.16 (0.69, 1.93) | 0.58 | 1.19 (0.69, 2.04) | 0.53 |
| **Surgery** |  |  |  |  |
| No/ Unknown | Reference |  | Reference |  |
| Yes | 1.05 (0.79, 1.4) | 0.75 | 1.21 (0.83, 1.76) | 0.33 |
| **Chemotherapy** |  |  |  |  |
| No/Unknown | Reference |  | Reference |  |
| Yes | 1.14 (0.8, 1.63) | 0.46 | 1.45 (0.91, 2.3) | 0.12 |
| **External beam radiotherapy** |  |  |  |  |
| No/Unknown | Reference |  | Reference |  |
| Yes | 0.87 (0.67-1.13) | 0.29 | 0.81 (0.56, 1.16) | 0.25 |

Supplementary Table 7. Univariable and Multivariable Competing Risk Regression Analyses of the Risk of Developing Any Second Primary Malignancies (SPMs) Among Cervical Cancer Patients After Propensity Score Matching (PSM)^(a)^ Between Radiotherapy and Non-Radiotherapy Groups

|  | **Univariable Competing Risk Regression** | | **Multivariable Competing Risk Regression** | |
| --- | --- | --- | --- | --- |
| **Characteristics** | **sHR (95% CI)** | ***P* value** | **sHR (95% CI)** | ***P* value** |
| **Age at CC diagnosis-y** | 1.01 (1.00-1.01) | < 0.001 | 1.01 (1.01-1.01) | < 0.001 |
| **Years of CC diagnosis** | 0.99 (0.98-0.99) | < 0.001 | 0.98 (0.97-0.99) | < 0.001 |
| **Race** |  |  |  |  |
| Hispanic | Reference |  | Reference |  |
| Non-Hispanic Black | 1.18 (0.99-1.41) | 0.066 | 1.19 (0.99-1.42) | 0.061 |
| Non-Hispanic White | 1.19 (1.04-1.36) | 0.014 | 1.12 (0.97-1.28) | 0.11 |
| Others | 1.11 (0.94-1.32) | 0.23 | 1.09 (0.91-1.3) | 0.35 |
| **Marital status** |  |  |  |  |
| Married | Reference |  | Reference |  |
| Divorced | 0.97 (0.87-1.09) | 0.64 | 0.97 (0.87-1.09) | 0.6 |
| Single | 0.76 (0.67-0.86) | < 0.001 | 0.85 (0.75-0.97) | 0.016 |
| Widowed | 0.84 (0.74-0.96) | 0.011 | 0.67 (0.58-0.78) | < 0.001 |
| Unknown | 0.88 (0.75-1.04) | 0.13 | 0.86 (0.73-1.02) | 0.09 |
| **Residence** |  |  |  |  |
| Large metro | Reference |  | Reference |  |
| City | 1.02 (0.89-1.17) | 0.77 | 1.07 (0.92-1.26) | 0.37 |
| Urban | 1.13 (0.9-1.41) | 0.28 | 1.19 (0.91-1.55) | 0.21 |
| Rural | 1.26 (1.01-1.57) | 0.043 | 1.34 (1-1.81) | 0.054 |
| Unknown | 1.26 (1.14-1.39) | < 0.001 | 0.7 (0.48-1) | 0.052 |
| **Income** |  |  |  |  |
| > $100000 | Reference |  | Reference |  |
| $85000-$100000 | 1.27 (1.04-1.54) | 0.017 | 1.32 (1.08-1.61) | 0.006 |
| $70000-$8500 | 1.17 (0.95-1.43) | 0.13 | 1.14 (0.93-1.41) | 0.2 |
| < $55000-$70000 | 1.25 (1.01-1.54) | 0.041 | 1.08 (0.82-1.4) | 0.59 |
| Unknown | 1.48 (1.24-1.76) | < 0.001 | 1.75 (1.15-2.67) | 0.009 |
| **FIGO staging** |  |  |  |  |
| I | Reference |  | Reference |  |
| II | 1.05 (0.93-1.18) | 0.45 | 0.98 (0.86-1.13) | 0.81 |
| III/ IV | 0.78 (0.64-0.95) | 0.016 | 0.73 (0.59-0.91) | 0.005 |
| Unknown | 1.11 (1.02-1.21) | 0.017 | 0.89 (0.75-1.05) | 0.17 |
| **Lymph node metastases** |  |  |  |  |
| No | Reference |  | Reference |  |
| Yes | 0.86 (0.72-1.02) | 0.086 | 0.87 (0.73-1.05) | 0.16 |
| Unknown | 1.08 (1-1.17) | 0.057 | 0.95 (0.84-1.09) | 0.48 |
| **Distant metastases** |  |  |  |  |
| M0 | Reference |  | Reference |  |
| Unknown | 1.19 (1.1-1.28) | < 0.001 | 0.98 (0.83-1.16) | 0.84 |
| **Histological type** |  |  |  |  |
| Squamous cell carcinoma | Reference |  | Reference |  |
| Adenocarcinoma | 0.91 (0.81-1.03) | 0.14 | 0.94 (0.82-1.07) | 0.33 |
| Other/Unknown | 0.81 (0.72-0.91) | < 0.001 | 0.82 (0.72-0.92) | 0.001 |
| **Grade** |  |  |  |  |
| I | Reference |  | Reference |  |
| II | 0.96 (0.82-1.12) | 0.62 | 0.96 (0.82-1.13) | 0.61 |
| III/IV | 1.04 (0.89-1.21) | 0.64 | 1.02 (0.87-1.2) | 0.77 |
| Other/Unknown | 1.05 (0.9-1.21) | 0.55 | 1.02 (0.87-1.19) | 0.83 |
| **Surgery** |  |  |  |  |
| No/ Unknown | Reference |  | Reference |  |
| Yes | 0.96 (0.89-1.05) | 0.39 | 1.11 (1-1.24) | 0.057 |
| **Chemotherapy** |  |  |  |  |
| No/Unknown | Reference |  | Reference |  |
| Yes | 0.84 (0.75-0.95) | 0.005 | 1.06 (0.9-1.25) | 0.47 |
| **External beam radiotherapy** |  |  |  |  |
| No/Unknown | Reference |  | Reference |  |
| Yes | 1.16 (1.08-1.26) | < 0.001 | 1.2 (1.08-1.33) | 0.001 |

(a) In addition to minimize biases related to treatment assignment, patients in the No/Unknown external beam radiotherapy (EBRT) group were matched to their nearest neighbors in a 1:1 ratio without replacement based on propensity scores. This approach aimed to balance the baseline characteristics between the EBRT and No/Unknown EBRT groups.

Supplementary Table 8. Univariable and Multivariable Competing Risk Regression Analyses of the Risk of Developing Any Pelvic Second Primary Malignancies (SPMs) Among Cervical Cancer Patients After Propensity Score Matching (PSM)^(a)^ Between Radiotherapy and Non-Radiotherapy Groups

|  | **Univariable Competing Risk Regression** | | **Multivariable Competing Risk Regression** | |
| --- | --- | --- | --- | --- |
| **Characteristics** | **sHR (95% CI)** | ***P* value** | **sHR (95% CI)** | ***P* value** |
| **Age at CC diagnosis-y** | 1.01 (1.00-1.01) | < 0.001 | 1.01 (1-1.01) | 0.016 |
| **Years of CC diagnosis** | 0.98 (0.97-0.99) | < 0.001 | 0.97 (0.96-0.99) | 0.003 |
| **Race** |  |  |  |  |
| Hispanic | Reference |  | Reference |  |
| Non-Hispanic Black | 1.28 (0.94-1.74) | 0.11 | 1.33 (0.97-1.82) | 0.078 |
| Non-Hispanic White | 1.09 (0.86-1.39) | 0.47 | 1.07 (0.84-1.36) | 0.59 |
| Others | 1 (0.73-1.37) | 0.99 | 1 (0.73-1.38) | 1 |
| **Marital status** |  |  |  |  |
| Married | Reference |  | Reference |  |
| Divorced | 1.04 (0.85-1.28) | 0.69 | 1.03 (0.84-1.26) | 0.79 |
| Single | 0.75 (0.59-0.95) | 0.015 | 0.84 (0.66-1.07) | 0.15 |
| Widowed | 0.84 (0.66-1.06) | 0.15 | 0.62 (0.48-0.81) | < 0.001 |
| Unknown | 0.8 (0.58-1.1) | 0.16 | 0.73 (0.53-1) | 0.053 |
| **Residence** |  |  |  |  |
| Large metro | Reference |  | Reference |  |
| City | 1.14 (0.88-1.48) | 0.32 | 1.19 (0.88-1.6) | 0.26 |
| Urban | 1.4 (0.95-2.07) | 0.087 | 1.3 (0.78-2.16) | 0.31 |
| Rural | 1.02 (0.64-1.62) | 0.95 | 0.95 (0.52-1.74) | 0.87 |
| Unknown | 1.47 (1.22-1.77) | < 0.001 | 0.75 (0.39-1.44) | 0.39 |
| **Income** |  |  |  |  |
| > $100000 | Reference |  | Reference |  |
| $85000-$100000 | 1.54 (1.05-2.25) | 0.025 | 1.58 (1.08-2.32) | 0.019 |
| $70000-$8500 | 1.17 (0.78-1.76) | 0.45 | 1.09 (0.72-1.65) | 0.69 |
| < $55000-$70000 | 1.55 (1.03-2.31) | 0.034 | 1.37 (0.82-2.27) | 0.23 |
| Unknown | 1.88 (1.33-2.65) | < 0.001 | 1.62 (0.74-3.54) | 0.22 |
| **FIGO staging** |  |  |  |  |
| I | Reference |  | Reference |  |
| II | 1.43 (1.16-1.78) | 0.001 | 1 (0.78-1.28) | 0.99 |
| III/ IV | 0.98 (0.69-1.39) | 0.91 | 0.66 (0.44-0.97) | 0.035 |
| Unknown | 1.48 (1.26-1.73) | < 0.001 | 0.88 (0.65-1.19) | 0.4 |
| **Lymph node metastases** |  |  |  |  |
| No | Reference |  | Reference |  |
| Yes | 0.9 (0.65-1.24) | 0.51 | 0.77 (0.54-1.09) | 0.14 |
| Unknown | 1.39 (1.2-1.61) | < 0.001 | 1.08 (0.85-1.37) | 0.53 |
| **Distant metastases** |  |  |  |  |
| M0 | Reference |  | Reference |  |
| Unknown | 1.38 (1.2-1.59) | < 0.001 | 1.01 (0.73-1.39) | 0.96 |
| **Histological type** |  |  |  |  |
| Squamous cell carcinoma | Reference |  | Reference |  |
| Adenocarcinoma | 0.87 (0.69-1.1) | 0.24 | 0.99 (0.78-1.26) | 0.96 |
| Other/Unknown | 0.73 (0.58-0.91) | 0.006 | 0.78 (0.62-0.99) | 0.039 |
| **Grade** |  |  |  |  |
| I | Reference |  | Reference |  |
| II | 0.99 (0.74-1.32) | 0.95 | 0.94 (0.7-1.26) | 0.66 |
| III/IV | 1.03 (0.77-1.38) | 0.83 | 0.93 (0.69-1.25) | 0.64 |
| Other/Unknown | 1.06 (0.81-1.39) | 0.69 | 1.01 (0.76-1.33) | 0.97 |
| **Surgery** |  |  |  |  |
| No/ Unknown | Reference |  | Reference |  |
| Yes | 0.63 (0.55-0.73) | < 0.001 | 0.89 (0.74-1.08) | 0.25 |
| **Chemotherapy** |  |  |  |  |
| No/Unknown | Reference |  | Reference |  |
| Yes | 0.95 (0.77-1.17) | 0.64 | 1.12 (0.83-1.5) | 0.47 |
| **External beam radiotherapy** |  |  |  |  |
| No/Unknown | Reference |  | Reference |  |
| Yes | 1.9 (1.64-2.21) | < 0.001 | 1.85 (1.52-2.25) | < 0.001 |

(a) In addition to minimize biases related to treatment assignment, patients in the No/Unknown external beam radiotherapy (EBRT) group were matched to their nearest neighbors in a 1:1 ratio without replacement based on propensity scores. This approach aimed to balance the baseline characteristics between the EBRT and No/Unknown EBRT groups.

Supplementary Table 9. Univariable and Multivariable Competing Risk Regression Analyses of the Risk of Developing Any Extrapelvic Second Primary Malignancies (SPMs) Among Cervical Cancer Patients After Propensity Score Matching (PSM)^(a)^ Between Radiotherapy and Non-Radiotherapy Groups

|  | **Univariable Competing Risk Regression** | | **Multivariable Competing Risk Regression** | |
| --- | --- | --- | --- | --- |
| **Characteristics** | **sHR (95% CI)** | ***P* value** | **sHR (95% CI)** | ***P* value** |
| **Age at CC diagnosis-y** | 1.00 (1.00-1.01) | 0.002 | 1.01 (1.01-1.01) | < 0.001 |
| **Years of CC diagnosis** | 0.99 (0.98-0.99) | < 0.001 | 0.99 (0.97-1) | 0.012 |
| **Race** |  |  |  |  |
| Hispanic | Reference |  | Reference |  |
| Non-Hispanic Black | 1.26 (0.99-1.61) | 0.055 | 1.26 (0.99-1.61) | 0.058 |
| Non-Hispanic White | 1.3 (1.08-1.57) | 0.006 | 1.2 (0.99-1.45) | 0.061 |
| Others | 1.3 (1.04-1.64) | 0.023 | 1.26 (1-1.6) | 0.052 |
| **Marital status** |  |  |  |  |
| Married | Reference |  | Reference |  |
| Divorced | 0.93 (0.8-1.08) | 0.36 | 0.94 (0.81-1.09) | 0.42 |
| Single | 0.76 (0.64-0.9) | 0.001 | 0.87 (0.73-1.03) | 0.1 |
| Widowed | 0.79 (0.67-0.94) | 0.007 | 0.67 (0.55-0.81) | < 0.001 |
| Unknown | 0.85 (0.69-1.05) | 0.14 | 0.86 (0.69-1.07) | 0.18 |
| **Residence** |  |  |  |  |
| Large metro | Reference |  | Reference |  |
| City | 1.05 (0.87-1.26) | 0.61 | 1.14 (0.93-1.39) | 0.2 |
| Urban | 1.06 (0.78-1.43) | 0.73 | 1.24 (0.88-1.75) | 0.22 |
| Rural | 1.3 (0.97-1.73) | 0.075 | 1.6 (1.09-2.34) | 0.017 |
| Unknown | 1.3 (1.14-1.48) | < 0.001 | 0.78 (0.5-1.23) | 0.29 |
| **Income** |  |  |  |  |
| > $100000 | Reference |  | Reference |  |
| $85000-$100000 | 1.27 (0.98-1.64) | 0.067 | 1.31 (1.01-1.69) | 0.044 |
| $70000-$8500 | 1.27 (0.98-1.65) | 0.073 | 1.24 (0.95-1.63) | 0.12 |
| < $55000-$70000 | 1.16 (0.88-1.53) | 0.3 | 0.95 (0.67-1.35) | 0.78 |
| Unknown | 1.52 (1.21-1.91) | < 0.001 | 1.69 (0.99-2.87) | 0.054 |
| **FIGO staging** |  |  |  |  |
| I | Reference |  | Reference |  |
| II | 0.86 (0.72-1.02) | 0.076 | 0.93 (0.77-1.12) | 0.45 |
| III/ IV | 0.66 (0.5-0.86) | 0.002 | 0.73 (0.55-0.98) | 0.039 |
| Unknown | 1.05 (0.94-1.17) | 0.41 | 0.88 (0.71-1.1) | 0.26 |
| **Lymph node metastases** |  |  |  |  |
| No | Reference |  | Reference |  |
| Yes | 0.76 (0.6-0.96) | 0.023 | 0.84 (0.66-1.09) | 0.19 |
| Unknown | 1.05 (0.94-1.16) | 0.41 | 0.97 (0.81-1.15) | 0.69 |
| **Distant metastases** |  |  |  |  |
| M0 | Reference |  | Reference |  |
| Unknown | 1.2 (1.09-1.33) | < 0.001 | 1.02 (0.82-1.27) | 0.88 |
| **Histological type** |  |  |  |  |
| Squamous cell carcinoma | Reference |  | Reference |  |
| Adenocarcinoma | 0.93 (0.79-1.09) | 0.39 | 0.92 (0.77-1.09) | 0.34 |
| Other/Unknown | 0.84 (0.73-0.98) | 0.028 | 0.82 (0.7-0.96) | 0.011 |
| **Grade** |  |  |  |  |
| I | Reference |  | Reference |  |
| II | 0.89 (0.73-1.1) | 0.28 | 0.93 (0.75-1.15) | 0.48 |
| III/IV | 0.98 (0.8-1.21) | 0.87 | 1.03 (0.83-1.27) | 0.81 |
| Other/Unknown | 1.04 (0.86-1.26) | 0.68 | 1.01 (0.83-1.24) | 0.89 |
| **Surgery** |  |  |  |  |
| No/ Unknown | Reference |  | Reference |  |
| Yes | 1.19 (1.06-1.33) | 0.003 | 1.25 (1.08-1.45) | 0.003 |
| **Chemotherapy** |  |  |  |  |
| No/Unknown | Reference |  | Reference |  |
| Yes | 0.73 (0.62-0.87) | < 0.001 | 1.03 (0.83-1.27) | 0.8 |
| **External beam radiotherapy** |  |  |  |  |
| No/Unknown | Reference |  | Reference |  |
| Yes | 0.92 (0.83-1.02)  (a) In addition to minimize biases related to treatment assignment, patients in the No/Unknown external beam radiotherapy (EBRT) group were matched to their nearest neighbors in a 1:1 ratio without replacement based on propensity scores. This approach aimed to balance the baseline characteristics between the EBRT and No/Unknown EBRT groups. | 0.13 | 1.03 (0.9-1.18) | 0.69 |

**Supplementary Table 9. Univariable and Multivariable Competing Risk Regression Analyses of the Risk of Developing Any Extrapelvic Second Primary Malignancies (SPMs) Among Cervical Cancer Patients After Propensity Score Matching (PSM)^(a)^ Between Radiotherapy and Non-Radiotherapy Groups**

Supplementary Table 10. Univariable and Multivariable Competing Risk Regression Analyses of the Risk of Developing Any Hematologic Second Primary Malignancies (SPMs) Among Cervical Cancer Patients After Propensity Score Matching (PSM)^(a)^ Between Radiotherapy and Non-Radiotherapy Groups

|  | **Univariable Competing Risk Regression** | | **Multivariable Competing Risk Regression** | |
| --- | --- | --- | --- | --- |
| **Characteristics** | **sHR (95% CI)** | ***P* value** | **sHR (95% CI)** | ***P* value** |
| **Age at CC diagnosis-y** | 1.01 (1.01-1.02) | < 0.001 | 1.03 (1.02-1.03) | < 0.001 |
| **Years of CC diagnosis** | 0.99 (0.99-1.01) | 0.69 | 0.97 (0.95-1) | 0.055 |
| **Race** |  |  |  |  |
| Hispanic | Reference |  | Reference |  |
| Non-Hispanic Black | 0.68 (0.35-1.29) | 0.24 | 0.67 (0.36-1.27) | 0.22 |
| Non-Hispanic White | 1.16 (0.76-1.78) | 0.49 | 1.11 (0.72-1.73) | 0.63 |
| Others | 0.64 (0.34-1.18) | 0.15 | 0.63 (0.34-1.18) | 0.15 |
| **Marital status** |  |  |  |  |
| Married | Reference |  | Reference |  |
| Divorced | 1 (0.69-1.46) | 0.99 | 0.98 (0.67-1.43) | 0.9 |
| Single | 0.65 (0.42-1.02) | 0.063 | 0.75 (0.47-1.19) | 0.23 |
| Widowed | 0.81 (0.53-1.26) | 0.36 | 0.58 (0.35-0.95) | 0.031 |
| Unknown | 1.24 (0.78-1.99) | 0.37 | 1.35 (0.83-2.22) | 0.23 |
| **Residence** |  |  |  |  |
| Large metro | Reference |  | Reference |  |
| City | 0.69 (0.43-1.11) | 0.12 | 0.59 (0.35-0.97) | 0.04 |
| Urban | 0.98 (0.49-1.97) | 0.95 | 0.69 (0.28-1.7) | 0.42 |
| Rural | 1.69 (0.94-3.02) | 0.077 | 1.06 (0.44-2.55) | 0.9 |
| Unknown | 0.98 (0.73-1.32) | 0.9 | 0.28 (0.04-2.05) | 0.21 |
| **Income** |  |  |  |  |
| > $100000 | Reference |  | Reference |  |
| $85000-$100000 | 0.74 (0.43-1.28) | 0.28 | 0.82 (0.47-1.43) | 0.48 |
| $70000-$8500 | 0.65 (0.36-1.17) | 0.15 | 0.74 (0.41-1.34) | 0.32 |
| < $55000-$70000 | 1.07 (0.62-1.87) | 0.8 | 1.19 (0.54-2.62) | 0.67 |
| Unknown | 0.91 (0.57-1.43) | 0.67 | 3.82 (0.49-29.71) | 0.2 |
| **FIGO staging** |  |  |  |  |
| I | Reference |  | Reference |  |
| II | 1.14 (0.79-1.65) | 0.48 | 1.26 (0.83-1.92) | 0.28 |
| III/ IV | 0.7 (0.37-1.33) | 0.27 | 0.76 (0.37-1.55) | 0.44 |
| Unknown | 0.77 (0.56-1.05) | 0.096 | 1.02 (0.58-1.81) | 0.93 |
| **Lymph node metastases** |  |  |  |  |
| No | Reference |  | Reference |  |
| Yes | 1.04 (0.65-1.67) | 0.86 | 1.2 (0.7-2.06) | 0.51 |
| Unknown | 0.67 (0.5-0.89) | 0.005 | 0.55 (0.34-0.89) | 0.016 |
| **Distant metastases** |  |  |  |  |
| M0 | Reference |  | Reference |  |
| Unknown | 0.89 (0.69-1.16) | 0.4 | 0.69 (0.41-1.16) | 0.16 |
| **Histological type** |  |  |  |  |
| Squamous cell carcinoma | Reference |  | Reference |  |
| Adenocarcinoma | 0.96 (0.64-1.42) | 0.82 | 0.94 (0.62-1.43) | 0.78 |
| Other/Unknown | 0.8 (0.54-1.19) | 0.27 | 0.8 (0.53-1.19) | 0.27 |
| **Grade** |  |  |  |  |
| I | Reference |  | Reference |  |
| II | 1.13 (0.66-1.95) | 0.65 | 1.12 (0.64-1.96) | 0.69 |
| III/IV | 1.25 (0.73-2.13) | 0.42 | 1.23 (0.7-2.15) | 0.47 |
| Other/Unknown | 1.11 (0.66-1.87) | 0.68 | 1.11 (0.65-1.92) | 0.7 |
| **Surgery** |  |  |  |  |
| No/ Unknown | Reference |  | Reference |  |
| Yes | 1.2 (0.9-1.6) | 0.220 | 1.21 (0.83-1.76) | 0.32 |
| **Chemotherapy** |  |  |  |  |
| No/Unknown | Reference |  | Reference |  |
| Yes | 1 (0.7-1.43) | 0.98 | 1.34 (0.84-2.14) | 0.22 |
| **External beam radiotherapy** |  |  |  |  |
| No/Unknown | Reference |  | Reference |  |
| Yes | 0.93 (0.72-1.21) | 0.6 | 0.8 (0.56-1.15) | 0.23 |

**Supplementary Table 10. Univariable and Multivariable Competing Risk Regression Analyses of the Risk of Developing Any Hematologic Second Primary Malignancies (SPMs) Among Cervical Cancer Patients After Propensity Score Matching (PSM)^(a)^ Between Radiotherapy and Non-Radiotherapy Groups**

(a) In addition to minimize biases related to treatment assignment, patients in the No/Unknown external beam radiotherapy (EBRT) group were matched to their nearest neighbors in a 1:1 ratio without replacement based on propensity scores. This approach aimed to balance the baseline characteristics between the EBRT and No/Unknown EBRT groups.

| **Second Primary Malignancy**  **Sites** | **EBRT *vs.***  **No/unknown-EBRT** | **Multivariable Competing Risks Regression Model** | | **Multivariable Competing Risks Regression Model (After PSM) (b)** | |
| --- | --- | --- | --- | --- | --- |
|  | ***no. of patients with events*** | **sHR (95% CI)** | ***P-*value** | **sHR (95% CI)** | ***P*-value** |
| **All solid cancers**  **(Within pelvis)** | 462/313 | 2.13 (1.81-2.50) | < 0.001 | 1.85 (1.52-2.25) | < 0.001 |
| Urinary Bladder | 73/43 | 2.3 (1.32-4) | 0.003 | 2.04 (1.21-3.43) | 0.007 |
| Rectum | 61/32 | 3.09 (1.72-5.55) | < 0.001 | 2.94 (1.61-5.37) | < 0.001 |
| Colon, NOS | 125/107 | 1.65 (1.17-2.32) | 0.004 | 1.52 (1.09-2.13) | 0.015 |
| Small Intestine | 10/5 | 3.73 (1.04-13.41) | 0.044 | 3.08 (0.93-10.12) | 0.065 |
| Ovary | 45/24 | 2.03 (1.04-3.97) | 0.038 | 1.88 (0.98-3.61) | 0.057 |
| Corpus Uteri | 49/9 | 3.23 (1.15-9.09) | 0.027 | 2.83 (1.07-7.5) | 0.037 |
| Vagina | 23/28 | 1.38 (0.62-3.1) | 0.43 | 1.28 (0.6-2.74) | 0.52 |
| Vulva | 28/14 | 3.65 (1.68-7.93) | 0.001 | 3.62 (1.57-8.37) | 0.003 |
| Anal Canal | 6/26 | 0.51 (0.17-1.57) | 0.24 | 0.46 (0.15-1.43) | 0.18 |
| Total of others **(a)** | 42/25 | 3.06 (1.46-6.43) | 0.003 | 2.67 (1.34-5.31) | 0.005 |
| **All solid cancers**  **(Out of pelvis)** | 637/936 | 1.03 (0.92-1.15) | 0.59 | 1.03 (0.9-1.18) | 0.69 |
| Breast | 182/413 | 0.73 (0.58-0.94) | 0.013 | 0.71 (0.56-0.9) | 0.004 |
| Lung and Bronchus | 264/255 | 1.53 (1.21-1.93) | < 0.001 | 1.44 (1.15-1.81) | 0.002 |
| Kidney and Renal Pelvis | 19/40 | 0.58 (0.28-1.2) | 0.14 | 0.56 (0.27-1.14) | 0.11 |
| Pancreas | 25/29 | 1.01 (0.5-2.06) | 0.97 | 0.96 (0.49-1.89) | 0.91 |
| Thyroid | 15/35 | 1.42 (0.69-2.91) | 0.34 | 1.29 (0.63-2.62) | 0.48 |
| Stomach | 25/22 | 1.25 (0.53-2.95) | 0.62 | 1.15 (0.5-2.66) | 0.74 |
| Melanoma | 16/30 | 1.15 (0.51-2.56) | 0.74 | 1.11 (0.52-2.41) | 0.78 |
| Liver | 5/16 | 0.48 (0.14-1.62) | 0.24 | 0.47 (0.17-1.36) | 0.17 |
| Larynx | 5/12 | 0.53 (0.18-1.6) | 0.26 | 0.53 (0.2-1.45) | 0.22 |
| Brain | 6/10 | 1.77 (0.59-5.28) | 0.3 | 1.61 (0.6-4.33) | 0.35 |
| Esophagus | 8/8 | 1.46 (0.53-4.04) | 0.47 | 1.48 (0.58-3.77) | 0.42 |
| Total of others **(a)** | 67/66 | 1.61 (0.99-2.61) | 0.055 | 1.51 (0.94-2.42) | 0.088 |
| **All hematologic malignancies** | 101/137 | 0.81 (0.56, 1.16) | 0.25 | 0.8 (0.56-1.15) | 0.23 |
| Lymphoma | 57/67 | 0.92 (0.54-1.59) | 0.77 | 0.91 (0.53-1.54) | 0.72 |
| Lymphocytic leukemia | 7/18 | 0.59 (0.22-1.57) | 0.29 | 0.61 (0.24-1.57) | 0.3 |
| Nonlymphocytic leukemia | 20/15 | 1.35 (0.54-3.37) | 0.52 | 1.32 (0.55-3.15) | 0.53 |
| Myeloma | 10/15 | 0.82 (0.39-1.75) | 0.61 | 0.83 (0.39-1.76) | 0.62 |
| Total of others **(a)** | 7/22 | 0.33 (0.12-0.88) | 0.027 | 0.35 (0.13-0.91) | 0.032 |

Supplementary Table 11. Multivariable Competing Risk Analysis of the Cumulative Incidence of Second Primary Malignancies Among Cervical Cancer Patients According to Receipt of EBRT

(a) In addition to the cancers listed in this table, other cancer types also include pelvic tumors, extra pelvic tumors, and hematologic malignancies with fewer than 15 total cases (see Supplementary Table 1 for details).

(b) To minimize biases related to treatment assignment, patients in the No/Unknown EBRT group were matched to their nearest neighbors in a 1:1 ratio without replacement based on propensity scores. This approach aimed to balance the baseline characteristics between the EBRT and No/Unknown EBRT groups.

Supplementary Table 12. Comparison of 5 - to 15- Year Cumulative Incidence of Second Primary Malignancy (with Death as a Competing Event) in Cervical Cancer Patients after EBRT: A Comparison between Patients Diagnosed in 1975 -1994 and 1995 -2006

| **Second Primary Malignancy**  **Sites** | **Cumulative Incidence**  **(5-15 Years, 95% CI) (b)** | | **Fine & Gray Competing Risks**  **Regression Model (1995-2006 (a) vs. 1975-1994)** | |
| --- | --- | --- | --- | --- |
|  | **Diagnosed between**  **1975-1994** | **Diagnosed between**  **1995-2006 (a)** | **sHR (95% CI)** | ***P* value** |
| All solid cancers (within pelvis) | 3.92% (3.34%-4.59%) | 2.85% (2.19%-3.69%) | 0.72 (0.53-0.98) | 0.036 |
| All solid cancers (Out of pelvis) | 6.22% (5.50%-7.03%) | 5.46% (4.55%-6.55%) | 0.88 (0.70-1.10) | 0.259 |
| All hematologic malignancies | 0.84% (0.62%-1.14%) | 0.70% (0.44%-1.13%) | 0.91 (0.51-1.60) | 0.750 |
| Breast | 1.64% (1.27%-2.11%) | 2.03% (1.49%-2.77%) | 1.24 (0.83-1.87) | 0.288 |
| Lung and Bronchus | 3.37% (2.83%-4.01%)  **(a)** Based on the SEER database submitted in November 2023, patients diagnosed after 2006 with a minimum follow-up time of less than 15 years will no longer be included in the comparison of 5- to 15-year cumulative incidence rates among patients diagnosed in different eras.  **(b)** Due to the differences in follow-up duration among patients diagnosed in different eras, for those with a follow-up time exceeding 15 years, the outcome status is uniformly defined as 0 (indicating that no events occurred). | 2.23% (1.66%-3.01%) | 0.66 (0.47-0.94) | 0.020 |

#

| **Second Primary Malignancy**  **Sites** | **Cumulative Incidence**  **(5-15 Years, 95% CI) (b)** | | **Fine & Gray Competing Risks**  **Regression Model (2002-2006 (a) vs. 1975-2001)** | |
| --- | --- | --- | --- | --- |
|  | **Diagnosed between**  **1975-2001** | **Diagnosed between**  **2002-2006 (a)** | **sHR (95% CI)** | ***P* value** |
| All solid cancers (within pelvis) | 3.76% (3.27%-4.34%) | 2.27% (1.44%-3.58%) | 0.59 (0.36-0.95) | 0.030 |
| All solid cancers (Out of pelvis) | 6.13% (5.50%-6.83%) | 4.91% (3.64%-6.61%) | 0.80 (0.58-1.01) | 0.178 |
| All hematologic malignancies | 0.79% (0.60%-1.04%) | 0.80% (0.40%-1.60%) | 1.25 (0.59-2.64) | 0.979 |
| Breast | 1.72% (1.38%-2.13%) | 2.12% (1.33%-3.40%) | 1.23 (0.73-2.08) | 0.424 |
| Lung and Bronchus | 3.25% (2.78%-3.79%) | 1.62% (0.94%-2,77%) | 0.50 (0.29-0.89) | 0.016 |

Supplementary Table 13. Comparison of 5 - to 15- Year Cumulative Incidence of Second Primary Malignancy (with Death as a Competing Event) in Cervical Cancer Patients after EBRT: A Comparison between Patients Diagnosed in 1975 -2001 and 2002-2006

**(a)** Based on the SEER database submitted in November 2023, patients diagnosed after 2006 with a minimum follow-up time of less than 15 years will no longer be included in the comparison of 5- to 15-year cumulative incidence rates among patients diagnosed in different eras.

**(b)** Due to the differences in follow-up duration among patients diagnosed in different eras, for those with a follow-up time exceeding 15 years, the outcome status is uniformly defined as 0 (indicating that no events occurred).

Supplementary Table 14. The Influence of Brachytherapy on the Incidence of SPMs in Patients with Cervical Cancer

| **Second Primary Malignancy**  **Sites** | **Brachytherapy Only vs.**  **Non-Radiotherapy(a)** | **Fine & Gray Competing Risks**  **Regression Model** | | |
| --- | --- | --- | --- | --- |
|  | ***no. of patients with events*** |  | **sHR (95% CI)** | ***P* value** |
| **All solid cancers (within pelvis)** | 16/297 |  | 3.04 (1.83-5.04) | < 0.001 |
| Urinary Bladder | 5/38 |  | 8.05 (3.22-20.15) | < 0.001 |
| Rectum | 1/31 |  | 1.65 (0.23-11.9) | 0.620 |
| Colon, NOS | 2/105 |  | 1.12 (0.28-4.54) | 0.870 |
| Small Intestine | 0/5 |  | - | - |
| Ovary | 0/24 |  | - | - |
| Corpus Uteri | 3/6 |  | 28.44 (7.32-110.46) | < 0.001 |
| Vagina | 2/26 |  | 3.49 (0.83-14.66) | 0.088 |
| Vulva | 1/13 |  | 4.1 (0.56-29.9) | 0.160 |
| Anus, Anal Canal and  Anorectum | 0/26 |  | - | - |
| Total of others **(b)** | 2/23 |  | 5.2 (1.23-22) | 0.025 |
| **All hematologic malignancies** | 5/132 |  | 1.60 (0.66-3.91) | 0.300 |
| Lymphoma | 3/64 |  | 1.92(0.6-6.07) | 0.270 |
| Lymphocytic leukemia | 0/18 |  | - | - |
| Nonlymphocytic leukemia | 1/14 |  | 3.31(0.45-24.44) | 0.240 |
| Myeloma | 0/15 |  | - | - |
| Total of others **(b)** | 1/21 |  | 2.01(0.27-15.07) | 0.500 |
| **All solid cancers (Out of pelvis)** | 15/921 |  | 0.96 (0.58-1.61) | 0.890 |
| Breast | 7/336 |  | 1.00 (0.47-2.11) | 1.000 |
| Lung and Bronchus | 3/332 |  | 0.69 (0.22-2.16) | 0.530 |

**(a)** To exclude the impact of external beam radiation therapy (EBRT), patients in the brachytherapy group received brachytherapy only, without EBRT. Patients in the non-radiotherapy group received neither brachytherapy nor EBRT.

**(b)** In addition to the cancers listed in this table, other cancer types also include pelvic tumors, extra pelvic tumors, and hematologic malignancies with fewer than 15 total cases (see Supplementary Table 1 for details). Abbreviation: SPMs, second primary malignancies.

**(a)** To exclude the impact of external beam radiation therapy (EBRT), patients in the brachytherapy group received brachytherapy only, without EBRT. Patients in the non-radiotherapy group received neither brachytherapy nor EBRT.

**(b)** In addition to the cancers listed in this table, other cancer types also include pelvic tumors, extra pelvic tumors, and hematologic malignancies with fewer than 15 total cases (see Supplementary Table 1 for details). Abbreviation: SPMs, second primary malignancies.

Supplementary Table 15. Baseline Characteristics After Propensity Score Matching Between Chemotherapy and No/Unknown Chemotherapy Groups (t-test or χ² test)

**Supplementary Table 15.** **Baseline Characteristics After Propensity Score Matching Between Chemotherapy and No/Unknown Chemotherapy Groups (t-test or χ² test)**

| **Characteristics** | **Chemotherapy,**  **No. (%)** | **No or Unknown**  **Chemotherapy, No. (%)** | ***P*-value** | **SMD** |
| --- | --- | --- | --- | --- |
| Total | 5122 | 5122 | - | - |
| Age at CC diagnosis, mean (SD), y | 49.71 (13.11) | 48.28 (14.96) | <0.001 | 0.517 |
| Years of CC diagnosis, mean | 2007 (8.44) | 2003 (11.47) | <0.001 | 0.227 |
| Race |  |  | 0.123 | 0.048 |
| Hispanic | 793 (15.5) | 724 (14.1) |  |  |
| Non-Hispanic Black | 580 (11.3) | 546 (10.7) |  |  |
| Non-Hispanic White | 2996 (58.5) | 3095 (60.4) |  |  |
| Others | 753 (14.7) | 757 (14.8) |  |  |
| Marital status |  |  | <0.001 | 0.105 |
| Married | 2355 (46.0) | 2438 (47.6) |  |  |
| Divorced | 720 (14.1) | 679 (13.3) |  |  |
| Single | 1291 (25.2) | 1112 (21.7) |  |  |
| Widowed | 428 (8.4) | 522 (10.2) |  |  |
| Unknown | 328 (6.4) | 371 (7.2) |  |  |
| Residence |  |  | <0.001 | 0.361 |
| Large metro | 2571 (50.2) | 2285 (44.6) |  |  |
| City | 1641 (32.0) | 1453 (28.4) |  |  |
| Urban | 374 (7.3) | 346 (6.8) |  |  |
| Rural | 326 (6.4) | 302 (5.9) |  |  |
| Unknown | 210 (4.1) | 736 (14.4) |  |  |
| Income |  |  | <0.001 | 0.338 |
| > $100000 | 838 (16.4) | 816 (15.9) |  |  |
| $85000-$100000 | 1488 (29.1) | 1390 (27.1) |  |  |
| $70000-$8500 | 1503 (29.3) | 1277 (24.9) |  |  |
| < $55000-$70000 | 1102 (21.5) | 982 (19.2) |  |  |
| Unknown | 191 (3.7) | 657 (12.8) |  |  |
| FIGO staging (Version 2009) |  |  | <0.001 | 0.554 |
| I | 1892 (36.9) | 3068 (59.9) |  |  |
| II | 1977 (38.6) | 1196 (23.4) |  |  |
| III/ IV | 1015 (19.8) | 452 (8.8) |  |  |
| Unknown | 238 (4.6) | 406 (7.9) |  |  |
| Lymph node metastases |  |  | <0.001 | 0.538 |
| Yes | 1565 (30.6) | 528 (10.3) |  |  |
| No | 2970 (58.0) | 3560 (69.5) |  |  |
| Unknown | 587 (11.5) | 1034 (20.2) |  |  |
| Distant metastases |  |  | <0.001 | 0.282 |
| M0 | 4837 (94.4) | 4413 (86.2) |  |  |
| Unknown | 285 (5.6) | 709 (13.8) |  |  |
| Histological type |  |  | <0.001 | 0.112 |
| Squamous cell carcinoma | 3675 (71.7) | 3427 (66.9) |  |  |
| Adenocarcinoma | 648 (12.7) | 817 (16.0) |  |  |
| Other/Unknown | 799 (15.6) | 878 (17.1) |  |  |
| Grade |  | <0.001 | <0.001 | 0.106 |
| I | 250 (4.9) | 335 (6.5) |  |  |
| II | 1623 (31.7) | 1633 (31.9) |  |  |
| III/IV | 1680 (32.8) | 1478 (28.9) |  |  |
| Other/Unknown | 3807 (33.1) | 5091 (44.3) |  |  |
| Surgery |  |  | <0.001 | 0.402 |
| Yes | 2371 (46.3) | 3373 (65.9) |  |  |
| No/ Unknown | 2751 (53.7) | 1749 (34.1) |  |  |
| EBRT |  |  | <0.001 | 0.711 |
| Yes | 4582 (89.5) | 3096 (60.4) |  |  |
| No/Unknown | 540 (10.5) | 2026 (39.6) |  |  |

Abbreviations: EBRT, external beam radiotherapy; SMD, standardized mean difference.

Abbreviations: EBRT, external beam radiotherapy; SMD, standardized mean difference.

Supplementary Table 16. Univariable and Multivariable Competing Risk Regression Analyses of the Risk of Developing Any Second Primary Malignancies (SPMs) Among Cervical Cancer Patients After Propensity Score Matching (PSM)^(a)^ Between Chemotherapy and Non-Chemotherapy Groups

**Supplementary Table 16. Univariable and Multivariable Competing Risk Regression Analyses of the Risk of Developing Any Second Primary Malignancies (SPMs) Among Cervical Cancer Patients After Propensity Score Matching (PSM)^(a)^ Between Chemotherapy and Non-Chemotherapy Groups**

|  | **Univariable Competing Risk Regression** | | **Multivariable Competing Risk Regression** | |
| --- | --- | --- | --- | --- |
| **Characteristics** | **sHR (95% CI)** | ***P* value** | **sHR (95% CI)** | ***P* value** |
| **Age at CC diagnosis-y** | 1.01 (1.00-1.02) | < 0.001 | 1.02 (1.01-1.02) | < 0.001 |
| **Years of CC diagnosis** | 0.98 (0.97-0.98) | < 0.001 | 0.97 (0.96-0.98) | < 0.001 |
| **Race** |  |  |  |  |
| Hispanic | Reference |  | Reference |  |
| Non-Hispanic Black | 0.92 (0.68, 1.25) | 0.59 | 0.99 (0.72-1.36) | 0.94 |
| Non-Hispanic White | 1.04 (0.83, 1.3) | 0.76 | 0.98 (0.78-1.23) | 0.87 |
| Others | 0.84 (0.63, 1.13) | 0.25 | 0.83 (0.61-1.13) | 0.23 |
| **Marital status** |  |  |  |  |
| Married | Reference |  | Reference |  |
| Divorced | 0.98 (0.8, 1.21) | 0.88 | 0.94 (0.76-1.16) | 0.56 |
| Single | 0.72 (0.59, 0.89) | 0.003 | 0.83 (0.67-1.04) | 0.099 |
| Widowed | 0.84 (0.66, 1.09) | 0.19 | 0.64 (0.48-0.84) | 0.002 |
| Unknown | 0.74 (0.51, 1.05) | 0.093 | 0.76 (0.53-1.09) | 0.14 |
| **Residence** |  |  |  |  |
| Large metro | Reference |  | Reference |  |
| City | 1.22 (1.02, 1.46) | 0.032 | 1.29 (1.06-1.58) | 0.013 |
| Urban | 1.21 (0.9, 1.62) | 0.21 | 1.22 (0.85-1.74) | 0.28 |
| Rural | 1.33 (0.98, 1.81) | 0.064 | 1.38 (0.92-2.07) | 0.12 |
| Unknown | 1.32 (1.08, 1.63) | 0.008 | 0.46 (0.19-1.12) | 0.088 |
| **Income** |  |  |  |  |
| > $100000 | Reference |  | Reference |  |
| $85000-$100000 | 1.08 (0.83, 1.4) | 0.55 | 1.15 (0.88-1.49) | 0.31 |
| $70000-$8500 | 0.99 (0.75, 1.29) | 0.93 | 0.95 (0.72-1.25) | 0.7 |
| < $55000-$70000 | 1.16 (0.88, 1.52) | 0.29 | 0.95 (0.68-1.34) | 0.77 |
| Unknown | 1.36 (1.03, 1.8) | 0.029 | 2.27 (0.86-5.94) | 0.096 |
| **FIGO staging** |  |  |  |  |
| I | Reference |  | Reference |  |
| II | 1.17 (0.99, 1.38) | 0.067 | 1.02 (0.84-1.23) | 0.86 |
| III/ IV | 0.97 (0.78, 1.21) | 0.79 | 0.81 (0.62-1.04) | 0.1 |
| Unknown | 0.98 (0.72, 1.34) | 0.9 | 0.86 (0.53-1.4) | 0.55 |
| **Lymph node metastases** |  |  |  |  |
| No | Reference |  | Reference |  |
| Yes | 0.85 (0.7, 1.04) | 0.12 | 0.86 (0.7-1.07) | 0.17 |
| Unknown | 1.13 (0.94, 1.35) | 0.19 | 1.04 (0.84-1.29) | 0.73 |
| **Distant metastases** |  |  |  |  |
| M0 | Reference |  | Reference |  |
| Unknown | 1.11 (0.91, 1.36) | 0.31 | 0.96 (0.66-1.4) | 0.83 |
| **Histological type** |  |  |  |  |
| Squamous cell carcinoma | Reference |  | Reference |  |
| Adenocarcinoma | 0.77 (0.59, 1) | 0.053 | 0.78 (0.6-1.03) | 0.076 |
| Other/Unknown | 0.74 (0.6, 0.92) | 0.007 | 0.75 (0.59-0.94) | 0.012 |
| **Grade** |  |  |  |  |
| I | Reference |  | Reference |  |
| II | 0.8 (0.58, 1.1) | 0.17 | 0.76 (0.55-1.05) | 0.099 |
| III/IV | 0.89 (0.65, 1.22) | 0.47 | 0.83 (0.6-1.15) | 0.26 |
| Other/Unknown | 0.89 (0.64, 1.22) | 0.46 | 0.86 (0.61-1.2) | 0.36 |
| **Surgery** |  |  |  |  |
| No/ Unknown | Reference |  | Reference |  |
| Yes | 0.94 (0.81, 1.08) | 0.38 | 1.11 (0.93-1.33) | 0.25 |
| **Chemotherapy** |  |  |  |  |
| No/Unknown | Reference |  | Reference |  |
| Yes | 0.89 (0.77, 1.03) | 0.11 | 1.1 (0.93-1.3) | 0.25 |
| **External beam radiotherapy** |  |  |  |  |
| No/Unknown | Reference |  | Reference |  |
| Yes | 1.55 (1.2, 2) | 0.001 | 1.2 (0.9-1.59) | 0.21 |

(a) In addition to minimize biases related to treatment assignment, patients in the No/Unknown chemotherapy group were matched to their nearest neighbors in a 1:1 ratio without replacement based on propensity scores. This approach aimed to balance the baseline characteristics between the chemotherapy and No/Unknown chemotherapy groups.

(a) In addition to minimize biases related to treatment assignment, patients in the No/Unknown chemotherapy group were matched to their nearest neighbors in a 1:1 ratio without replacement based on propensity scores. This approach aimed to balance the baseline characteristics between the chemotherapy and No/Unknown chemotherapy groups.

Supplementary Table 17. Univariable and Multivariable Competing Risk Regression Analyses of the Risk of Developing Any Pelvic Second Primary Malignancies (SPMs) Among Cervical Cancer Patients After Propensity Score Matching (PSM)^(a)^ Between Chemotherapy and Non-Chemotherapy Groups

**Supplementary Table 17. Univariable and Multivariable Competing Risk Regression Analyses of the Risk of Developing Any Pelvic Second Primary Malignancies (SPMs) Among Cervical Cancer Patients After Propensity Score Matching (PSM)^(a)^ Between Chemotherapy and Non-Chemotherapy Groups**

|  | **Univariable Competing Risk Regression** | | **Multivariable Competing Risk Regression** | |
| --- | --- | --- | --- | --- |
| **Characteristics** | **sHR (95% CI)** | ***P* value** | **sHR (95% CI)** | ***P* value** |
| **Age at CC diagnosis-y** | 1.01 (1.00-1.02) | 0.073 | 1.01 (1, 1.02) | 0.11 |
| **Years of CC diagnosis** | 0.97 (0.95-0.98) | < 0.001 | 0.96 (0.94, 0.98) | < 0.001 |
| **Race** |  |  |  |  |
| Hispanic | Reference |  | Reference |  |
| Non-Hispanic Black | 0.92 (0.56, 1.5) | 0.73 | 1.04 (0.61, 1.76) | 0.89 |
| Non-Hispanic White | 0.85 (0.59, 1.23) | 0.38 | 0.84 (0.58, 1.22) | 0.37 |
| Others | 0.81 (0.5, 1.3) | 0.37 | 0.81 (0.49, 1.35) | 0.42 |
| **Marital status** |  |  |  |  |
| Married | Reference |  | Reference |  |
| Divorced | 1.1 (0.78, 1.56) | 0.59 | 1.05 (0.74, 1.5) | 0.78 |
| Single | 0.8 (0.56, 1.15) | 0.23 | 0.88 (0.61, 1.28) | 0.51 |
| Widowed | 1.06 (0.71, 1.58) | 0.79 | 0.87 (0.55, 1.36) | 0.55 |
| Unknown | 0.9 (0.51, 1.59) | 0.71 | 0.89 (0.49, 1.59) | 0.68 |
| **Residence** |  |  |  |  |
| Large metro | Reference |  | Reference |  |
| City | 1.4 (1.02, 1.92) | 0.39 | 1.55 (1.08, 2.22) | 0.017 |
| Urban | 1.45 (0.89, 2.36) | 0.13 | 1.44 (0.76, 2.73) | 0.26 |
| Rural | 1.03 (0.55, 1.93) | 0.92 | 1.02 (0.48, 2.16) | 0.97 |
| Unknown | 1.76 (1.26, 2.45) | 0.001 | 0.67 (0.21, 2.16) | 0.5 |
| **Income** |  |  |  |  |
| > $100000 | Reference |  | Reference |  |
| $85000-$100000 | 1.28 (0.81, 2.02) | 0.29 | 1.36 (0.86, 2.16) | 0.19 |
| $70000-$8500 | 0.86 (0.52, 1.42) | 0.56 | 0.82 (0.49, 1.36) | 0.44 |
| < $55000-$70000 | 1.32 (0.82, 2.14) | 0.26 | 1.1 (0.6, 2.01) | 0.76 |
| Unknown | 1.79 (1.12, 2.87) | 0.015 | 1.9 (0.51, 7.07) | 0.34 |
| **FIGO staging** |  |  |  |  |
| I | Reference |  | Reference |  |
| II | 1.23 (0.93, 1.64) | 0.15 | 0.92 (0.67, 1.27) | 0.62 |
| III/ IV | 0.92 (0.62, 1.36) | 0.66 | 0.64 (0.4, 1.01) | 0.057 |
| Unknown | 1.16 (0.7, 1.94) | 0.56 | 0.9 (0.41, 1.98) | 0.8 |
| **Lymph node metastases** |  |  |  |  |
| No | Reference |  | Reference |  |
| Yes | 0.67 (0.46, 0.97) | 0.032 | 0.73 (0.5, 1.07) | 0.11 |
| Unknown | 1.13 (0.83, 1.52) | 0.44 | 0.92 (0.64, 1.33) | 0.66 |
| **Distant metastases** |  |  |  |  |
| M0 | Reference |  | Reference |  |
| Unknown | 1.3 (0.94, 1.8) | 0.11 | 0.89 (0.49, 1.6) | 0.69 |
| **Histological type** |  |  |  |  |
| Squamous cell carcinoma | Reference |  | Reference |  |
| Adenocarcinoma | 0.85 (0.55, 1.31) | 0.47 | 0.92 (0.6, 1.41) | 0.71 |
| Other/Unknown | 0.66 (0.44, 0.97) | 0.34 | 0.7 (0.46, 1.05) | 0.084 |
| **Grade** |  |  |  |  |
| I | Reference |  | Reference |  |
| II | 0.71 (0.42, 1.2) | 0.2 | 0.67 (0.39, 1.14) | 0.14 |
| III/IV | 0.74 (0.44, 1.24) | 0.25 | 0.7 (0.41, 1.18) | 0.18 |
| Other/Unknown | 0.9 (0.53, 1.52) | 0.68 | 0.81 (0.47, 1.38) | 0.43 |
| **Surgery** |  |  |  |  |
| No/ Unknown | Reference |  | Reference |  |
| Yes | 0.67 (0.52, 0.87) | 0.002 | 0.75 (0.55, 1.03) | 0.072 |
| **Chemotherapy** |  |  |  |  |
| No/Unknown | Reference |  | Reference |  |
| Yes | 0.85 (0.66, 1.09) | 0.21 | 1.17 (0.87, 1.58) | 0.3 |
| **External beam radiotherapy** |  |  |  |  |
| No/Unknown | Reference |  | Reference |  |
| Yes | 1.46 (0.94, 2.27) | 0.089 | 1.09 (0.67, 1.75) | 0.74 |

(a) In addition to minimize biases related to treatment assignment, patients in the No/Unknown chemotherapy group were matched to their nearest neighbors in a 1:1 ratio without replacement based on propensity scores. This approach aimed to balance the baseline characteristics between the chemotherapy and No/Unknown chemotherapy groups.

(a) In addition to minimize biases related to treatment assignment, patients in the No/Unknown chemotherapy group were matched to their nearest neighbors in a 1:1 ratio without replacement based on propensity scores. This approach aimed to balance the baseline characteristics between the chemotherapy and No/Unknown chemotherapy groups.

Supplementary Table 18. Univariable and Multivariable Competing Risk Regression Analyses of the Risk of Developing Any Extrapelvic Second Primary Malignancies (SPMs) Among Cervical Cancer Patients After Propensity Score Matching (PSM)^(a)^ Between Chemotherapy and Non-Chemotherapy Groups

**Supplementary Table 18. Univariable and Multivariable Competing Risk Regression Analyses of the Risk of Developing Any Extrapelvic Second Primary Malignancies (SPMs) Among Cervical Cancer Patients After Propensity Score Matching (PSM)^(a)^ Between Chemotherapy and Non-Chemotherapy Groups**

|  | **Univariable Competing Risk Regression** | | **Multivariable Competing Risk Regression** | |
| --- | --- | --- | --- | --- |
| **Characteristics** | **sHR (95% CI)** | ***P* value** | **sHR (95% CI)** | ***P* value** |
| **Age at CC diagnosis-y** | 1.01 (1.01-1.02) | < 0.001 | 1.02 (1.01, 1.03) | < 0.001 |
| **Years of CC diagnosis** | 0.98 (0.97-0.99) | < 0.001 | 0.98 (0.96, 0.99) | 0.008 |
| **Race** |  |  |  |  |
| Hispanic | Reference |  | Reference |  |
| Non-Hispanic Black | 1.02 (0.67, 1.56) | 0.93 | 1.08 (0.7, 1.67) | 0.73 |
| Non-Hispanic White | 1.1 (0.8, 1.52) | 0.54 | 1.02 (0.73, 1.41) | 0.92 |
| Others | 0.91 (0.6, 1.38) | 0.66 | 0.88 (0.57, 1.36) | 0.58 |
| **Marital status** |  |  |  |  |
| Married | Reference |  | Reference |  |
| Divorced | 0.9 (0.68, 1.21) | 0.5 | 0.86 (0.64, 1.15) | 0.31 |
| Single | 0.67 (0.5, 0.9) | 0.008 | 0.77 (0.57, 1.04) | 0.083 |
| Widowed | 0.66 (0.45, 0.96) | 0.029 | 0.47 (0.31, 0.7) | < 0.001 |
| Unknown | 0.56 (0.33, 0.97) | 0.038 | 0.58 (0.33, 1.01) | 0.054 |
| **Residence** |  |  |  |  |
| Large metro | Reference |  | Reference |  |
| City | 1.18 (0.92, 1.51) | 0.2 | 1.25 (0.95, 1.64) | 0.11 |
| Urban | 1.01 (0.65, 1.55) | 0.98 | 1.07 (0.65, 1.77) | 0.78 |
| Rural | 1.51 (1.02, 2.23) | 0.042 | 1.7 (0.99, 2.92) | 0.057 |
| Unknown | 1.06 (0.78, 1.45) | 0.71 | 0.35 (0.08, 1.47) | 0.15 |
| **Income** |  |  |  |  |
| > $100000 | Reference |  | Reference |  |
| $85000-$100000 | 1.13 (0.79, 1.63) | 0.49 | 1.2 (0.84, 1.73) | 0.32 |
| $70000-$8500 | 1.18 (0.82, 1.7) | 0.38 | 1.13 (0.77, 1.64) | 0.54 |
| < $55000-$70000 | 1.17 (0.8, 1.72) | 0.42 | 0.93 (0.58, 1.5) | 0.78 |
| Unknown | 1.2 (0.8, 1.8) | 0.38 | 2.7 (0.59, 12.42) | 0.2 |
| **FIGO staging** |  |  |  |  |
| I | Reference |  | Reference |  |
| II | 1.11 (0.88, 1.4) | 0.38 | 1.05 (0.81, 1.36) | 0.71 |
| III/ IV | 0.95 (0.7, 1.29) | 0.73 | 0.88 (0.63, 1.25) | 0.49 |
| Unknown | 0.86 (0.55, 1.36) | 0.53 | 0.74 (0.36, 1.54) | 0.42 |
| **Lymph node metastases** |  |  | 1.24 (0.92, 1.67) | 0.15 |
| No | Reference |  | Reference |  |
| Yes | 0.88 (0.66, 1.16) | 0.36 | 0.86 (0.65, 1.15) | 0.32 |
| Unknown | 1.19 (0.93, 1.53) | 0.17 | 1.24 (0.92, 1.67) | 0.15 |
| **Distant metastases** |  |  |  |  |
| M0 | Reference |  | Reference |  |
| Unknown | 0.97 (0.72, 1.32) | 0.87 | 1.04 (0.56, 1.94) | 0.89 |
| **Histological type** |  |  |  |  |
| Squamous cell carcinoma | Reference |  | Reference |  |
| Adenocarcinoma | 0.79 (0.55, 1.14) | 0.20 | 0.76 (0.52, 1.1) | 0.15 |
| Other/Unknown | 0.83 (0.63, 1.11) | 0.22 | 0.8 (0.59, 1.08) | 0.15 |
| **Grade** |  |  |  |  |
| I | Reference |  | Reference |  |
| II | 0.73 (0.48, 1.13) | 0.16 | 0.72 (0.46, 1.13) | 0.15 |
| III/IV | 0.86 (0.56, 1.32) | 0.49 | 0.83 (0.53, 1.29) | 0.4 |
| Other/Unknown | 0.78 (0.5, 1.21) | 0.27 | 0.81 (0.51, 1.29) | 0.38 |
| **Surgery** |  |  |  |  |
| No/ Unknown | Reference |  | Reference |  |
| Yes | 1.14 (0.93, 1.4) | 0.21 | 1.4 (1.09, 1.8) | 0.009 |
| **Chemotherapy** |  |  |  |  |
| No/Unknown | Reference |  | Reference |  |
| Yes | 0.87 (0.71, 1.07) | 0.17 | 1.02 (0.81, 1.28) | 0.87 |
| **External beam radiotherapy** |  |  |  |  |
| No/Unknown | Reference |  | Reference |  |
| Yes | 1.57 (1.1, 2.24) | 0.013  (a) In addition to minimize biases related to treatment assignment, patients in the No/Unknown chemotherapy group were matched to their nearest neighbors in a 1:1 ratio without replacement based on propensity scores. This approach aimed to balance the baseline characteristics between the chemotherapy and No/Unknown chemotherapy groups.  (a) In addition to minimize biases related to treatment assignment, patients in the No/Unknown chemotherapy group were matched to their nearest neighbors in a 1:1 ratio without replacement based on propensity scores. This approach aimed to balance the baseline characteristics between the chemotherapy and No/Unknown chemotherapy groups. | 1.29 (0.87, 1.92) | 0.2 |

Supplementary Table 19. Univariable and Multivariable Competing Risk Regression Analyses of the Risk of Developing Any Hematologic Second Primary Malignancies (SPMs) Among Cervical Cancer Patients After Propensity Score Matching (PSM)^(a)^ Between Chemotherapy and Non-Chemotherapy Groups

**Supplementary Table 19. Univariable and Multivariable Competing Risk Regression Analyses of the Risk of Developing Any Hematologic Second Primary Malignancies (SPMs) Among Cervical Cancer Patients After Propensity Score Matching (PSM)^(a)^ Between Chemotherapy and Non-Chemotherapy Groups**

|  | **Univariable Competing Risk Regression** | | **Multivariable Competing Risk Regression** | |
| --- | --- | --- | --- | --- |
| **Characteristics** | **sHR (95% CI)** | ***P* value** | **sHR (95% CI)** | ***P* value** |
| **Age at CC diagnosis-y** | 1.02 (1.00-1.03) | 0.007 | 1.03 (1.01, 1.05) | 0.001 |
| **Years of CC diagnosis** | 0.96 (0.94-0.99) | 0.007 | 0.96 (0.92, 0.99) | 0.022 |
| **Race** |  |  |  |  |
| Hispanic | Reference |  | Reference |  |
| Non-Hispanic Black | 0.43 (0.12, 1.64) | 0.22 | 0.5 (0.13, 1.87) | 0.3 |
| Non-Hispanic White | 1.37 (0.65, 2.89) | 0.4 | 1.37 (0.66, 2.85) | 0.4 |
| Others | 0.23 (0.05, 1.07) | 0.06 | 0.25 (0.05, 1.27) | 0.094 |
| **Marital status** |  |  |  |  |
| Married | Reference |  | Reference |  |
| Divorced | 1.38 (0.74, 2.59) | 0.31 | 1.21 (0.62, 2.36) | 0.58 |
| Single | 0.57 (0.26, 1.22) | 0.15 | 0.72 (0.33, 1.59) | 0.42 |
| Widowed | 0.81 (0.33, 1.94) | 0.63 | 0.53 (0.19, 1.45) | 0.22 |
| Unknown | 0.96 (0.34, 2.71) | 0.93 | 1.12 (0.38, 3.27) | 0.84 |
| **Residence** |  |  |  |  |
| Large metro | Reference |  | Reference |  |
| City | 1.2 (0.64, 2.25) | 0.57 | 0.96 (0.46, 1.99) | 0.91 |
| Urban | 1.64 (0.67, 4.03) | 0.28 | 0.94 (0.28, 3.19) | 0.92 |
| Rural | 1.71 (0.65, 4.49) | 0.28 | 0.93 (0.25, 3.41) | 0.91 |
| Unknown | 1.87 (0.96, 3.66) | 0.066 | NA | NA |
| **Income** |  |  |  |  |
| > $100000 | Reference |  | Reference |  |
| $85000-$100000 | 0.42 (0.19, 0.96) | 0.039 | 0.46 (0.2, 1.05) | 0.066 |
| $70000-$8500 | 0.49 (0.21, 1.1) | 0.049 | 0.47 (0.2, 1.08) | 0.076 |
| < $55000-$70000 | 0.98 (0.47, 2.07) | 0.97 | 0.9 (0.31, 2.58) | 0.84 |
| Unknown | 1.21 (0.56, 2.59) | 0.63 | NA | NA |
| **FIGO staging** |  |  |  |  |
| I | Reference |  | Reference |  |
| II | 1.73 (1, 3.02) | 0.052 | 1.36 (0.74, 2.48) | 0.32 |
| III/ IV | 1.11 (0.51, 2.46) | 0.79 | 0.71 (0.28, 1.8) | 0.47 |
| Unknown | 0.92 (0.28, 3.06) | 0.9 | 0.59 (0.13, 2.75) | 0.5 |
| **Lymph node metastases** |  |  |  |  |
| No | Reference |  | Reference |  |
| Yes | 1.2 (0.67, 2.16) | 0.54 | 1.16 (0.61, 2.22) | 0.66 |
| Unknown | 0.82 (0.41, 1.63) | 0.56 | 0.65 (0.3, 1.44) | 0.29 |
| **Distant metastases** |  |  |  |  |
| M0 | Reference |  | Reference |  |
| Unknown | 1.62 (0.87, 3.02) | 0.13 | 1.84 (0.65, 5.21) | 0.25 |
| **Histological type** |  |  |  |  |
| Squamous cell carcinoma | Reference |  | Reference |  |
| Adenocarcinoma | 0.35 (0.11, 1.1) | 0.072 | 0.4 (0.12, 1.3) | 0.13 |
| Other/Unknown | 0.45 (0.19, 1.05) | 0.052 | 0.47 (0.19, 1.14) | 0.094 |
| **Grade** |  |  |  |  |
| I | Reference |  | Reference |  |
| II | 1.69 (0.4, 7.19) | 0.48 | 1.28 (0.28, 5.77) | 0.75 |
| III/IV | 1.98 (0.47, 8.34) | 0.35 | 1.44 (0.33, 6.39) | 0.63 |
| Other/Unknown | 1.92 (0.44, 8.3) | 0.38 | 1.56 (0.34, 7.11) | 0.57 |
| **Surgery** |  |  |  |  |
| No/ Unknown | Reference |  | Reference |  |
| Yes | 0.87 (0.53, 1.43) | 0.59 | 1.19 (0.65, 2.17) | 0.58 |
| **Chemotherapy** |  |  |  |  |
| No/Unknown | Reference |  | Reference |  |
| Yes | 1.3 (0.8, 2.11) | 0.3 | 1.81 (1.09, 3.02) | 0.022 |
| **External beam radiotherapy** |  |  |  |  |
| No/Unknown | Reference |  | Reference |  |
| Yes | 1.85 (0.8, 4.26) | 0.15 | 0.98 (0.39, 2.47) | 0.97 |

(a) In addition to minimize biases related to treatment assignment, patients in the No/Unknown chemotherapy group were matched to their nearest neighbors in a 1:1 ratio without replacement based on propensity scores. This approach aimed to balance the baseline characteristics between the chemotherapy and No/Unknown chemotherapy groups.

(a) In addition to minimize biases related to treatment assignment, patients in the No/Unknown chemotherapy group were matched to their nearest neighbors in a 1:1 ratio without replacement based on propensity scores. This approach aimed to balance the baseline characteristics between the chemotherapy and No/Unknown chemotherapy groups.

Supplementary Table 20. Univariable Competing Risk Analysis of the Cumulative Incidence of Second Primary Malignancies Among Cervical Cancer Patients According to Receipt of Chemotherapy

Supplementary Table 20. Univariable Competing Risk Analysis of the Cumulative Incidence of Second Primary Malignancies Among Cervical Cancer Patients According to Receipt of Chemotherapy

| **Second Primary Malignancy**  **Sites** | **Chemotherapy *vs.***  **No/unknown-** **Chemotherapy** | **Univariable Competing Risks Regression Model** | | **Univariable Competing Risks Regression Model (After PSM) (b)** | |
| --- | --- | --- | --- | --- | --- |
|  | ***no. of patients with events*** | **sHR (95% CI)** | ***P-*value** | **sHR (95% CI)** | ***P*-value** |
| **All solid cancers**  **(Within pelvis)** | 102/673 | 1.41 (1.14-1.74) | 0.001 | 0.85 (0.66, 1.09) | 0.21 |
| Urinary Bladder | 18/98 | 1.94 (1.16-3.23) | 0.011 | 1.88 (0.9-3.93) | 0.091 |
| Rectum | 12/81 | 1.33 (0.72-2.44) | 0.36 | 0.93 (0.38-2.26) | 0.87 |
| Colon, NOS | 19/213 | 0.85 (0.53-1.36) | 0.49 | 0.78 (0.43-1.41) | 0.4 |
| Small Intestine | 2/13 | 1.45 (0.31-6.71) | 0.64 | 0.51 (0.09-3.04) | 0.46 |
| Ovary | 7/62 | 1.1 (0.5-2.43) | 0.81 | 0.99 (0.3-3.28) | 0.98 |
| Corpus Uteri | 18/40 | 3.95 (2.27-6.88) | < 0.001 | 1.65 (0.73-3.69) | 0.23 |
| Vagina | 6/45 | 1.04 (0.44-2.47) | 0.93 | 2.01 (0.51-7.99) | 0.32 |
| Vulva | 6/36 | 1.28 (0.54-3.04) | 0.57 | 0.79 (0.2-3.03) | 0.73 |
| Anal Canal | 1/31 | 0.34 (0.05-2.48) | 0.34 | NA | NA |
| Total of others **(a)** | 13/54 | 2.08 (1.15-3.75) | 0.016 | 1.84 (0.68-4.97) | 0.23 |
| **All solid cancers**  **(Out of pelvis)** | 160/1413 | 1.04 (0.88, 1.22) | 0.65 | 0.87 (0.71, 1.07) | 0.17 |
| Breast | 54/541 | 0.91 (0.69-1.2) | 0.5 | 1.05 (0.7-1.57) | 0.82 |
| Lung and Bronchus | 50/469 | 0.92 (0.69-1.24) | 0.59 | 0.85 (0.59-1.24) | 0.4 |
| Kidney and Renal Pelvis | 7/52 | 1.38 (0.63-3.04) | 0.42 | 4.79 (1.09-20.98) | 0.038 |
| Pancreas | 10/44 | 2.21 (1.11-4.41) | 0.024 | 2.66 (0.87-8.11) | 0.086 |
| Thyroid | 6/44 | 1.12 (0.47-2.7) | 0.79 | 0.63 (0.12-3.18) | 0.57 |
| Stomach | 5/42 | 1.08 (0.43-2.73) | 0.87 | 0.89 (0.16-4.97) | 0.89 |
| Melanoma | 6/40 | 1.33 (0.56-3.14) | 0.51 | 0.66 (0.19-2.29) | 0.51 |
| Liver | 2/19 | 1.19 (0.3-4.7) | 0.81 | NA | NA |
| Larynx | 0/17 | NA | NA | NA | NA |
| Brain | 2/14 | 1.38 (0.32-5.99) | 0.67 | 1.08 (0.1-11.2) | 0.95 |
| Esophagus | 2/14 | 1.47 (0.36-6.07) | 0.59 | NA | NA |
| Total of others **(a)** | 16/117 | 1.29 (0.77-2.15) | 0.33 | 1.06 (0.52-2.16) | 0.88 |
| **All hematologic malignancies** | 35/203 | 1.14 (0.8, 1.63) | 0.46 | 1.3 (0.8, 2.11) | 0.3 |
| Lymphoma | 20/104 | 1.24 (0.77-1.98) | 0.37 | 1.81 (1-3.27) | 0.051 |
| Lymphocytic leukemia | 2/23 | 0.63 (0.14-2.75) | 0.54 | NA | NA |
| Nonlymphocytic leukemia | 10/25 | 2.57 (1.27-5.2) | 0.009 | 1.79 (0.43-7.4) | 0.42 |
| Myeloma | 1/24 | 0.28 (0.04-2.12) | 0.22 | 0.31 (0.03-3.32) | 0.34 |
| Total of others **(a)** | 2/27 | 0.52 (0.13-2.2) | 0.38 | 1.3 (0.28-6.07) | 0.74 |

**(a)** In addition to the cancers listed in this table, other cancer types also include pelvic tumors, extra pelvic tumors, and hematologic malignancies with fewer than 15 total cases (see Supplementary Table 1 for details).

**(b)** To minimize biases related to treatment assignment, patients in the No/Unknown chemotherapy group were matched to their nearest neighbors in a 1:1 ratio without replacement based on propensity scores. This approach aimed to balance the baseline characteristics between the chemotherapy and No/Unknown chemotherapy groups.

**(a)** In addition to the cancers listed in this table, other cancer types also include pelvic tumors, extra pelvic tumors, and hematologic malignancies with fewer than 15 total cases (see Supplementary Table 1 for details).

**(b)** To minimize biases related to treatment assignment, patients in the No/Unknown chemotherapy group were matched to their nearest neighbors in a 1:1 ratio without replacement based on propensity scores. This approach aimed to balance the baseline characteristics between the chemotherapy and No/Unknown chemotherapy groups.

Supplementary Table 21. Multivariable Competing Risk Analysis of the Cumulative Incidence of Second Primary Malignancies Among Cervical Cancer Patients According to Receipt of Chemotherapy

Supplementary Table 21. Multivariable Competing Risk Analysis of the Cumulative Incidence of Second Primary Malignancies Among Cervical Cancer Patients According to Receipt of Chemotherapy

| **Second Primary Malignancy**  **Sites** | **Chemotherapy *vs.***  **No/unknown-** **Chemotherapy** | **Multivariable Competing Risks Regression Model** | | **Multivariable Competing Risks Regression Model (After PSM) (b)** | |
| --- | --- | --- | --- | --- | --- |
|  | ***no. of patients with events*** | **sHR (95% CI)** | ***P-*value** | **sHR (95% CI)** | ***P*-value** |
| **All solid cancers**  **(Within pelvis)** | 102/673 | 1.13 (0.83-1.52) | 0.44 | 0.85 (0.66-1.09) | 0.21 |
| Urinary Bladder | 18/98 | 1.81 (0.87-3.75) | 0.11 | 1.88 (0.9-3.93) | 0.091 |
| Rectum | 12/81 | 0.69 (0.29-1.61) | 0.39 | 0.93 (0.38-2.26) | 0.87 |
| Colon, NOS | 19/213 | 0.88 (0.47-1.66) | 0.69 | 0.78 (0.43-1.41) | 0.4 |
| Small Intestine | 2/13 | 0.51 (0.06-4.4) | 0.54 | 0.51 (0.09-3.04) | 0.46 |
| Ovary | 7/62 | 0.77 (0.28-2.11) | 0.61 | 0.99 (0.3-3.28) | 0.98 |
| Corpus Uteri | 18/40 | 2.15 (0.91-5.12) | 0.082 | 1.65 (0.73-3.69) | 0.23 |
| Vagina | 6/45 | 1.22 (0.33-4.52) | 0.76 | 2.01 (0.51-7.99) | 0.32 |
| Vulva | 6/36 | 0.67 (0.18-2.48) | 0.55 | 0.79 (0.2-3.03) | 0.73 |
| Anal Canal | 1/31 | 1.35 (0.15-11.94) | 0.79 | NA | NA |
| Total of others **(a)** | 13/54 | 1.57 (0.64-3.86) | 0.32 | 1.84 (0.68-4.97) | 0.23 |
| **All solid cancers**  **(Out of pelvis)** | 160/1413 | 1.11 (0.9, 1.37) | 0.34 | 1.07 (0.71-1.07) | 0.87 |
| Breast | 54/541 | 1.23 (0.86-1.77) | 0.26 | 1.05 (0.7-1.57) | 0.82 |
| Lung and Bronchus | 50/469 | 0.88 (0.61-1.28) | 0.51 | 0.85 (0.59-1.24) | 0.4 |
| Kidney and Renal Pelvis | 7/52 | 2.33 (0.82-6.6) | 0.11 | 4.79 (1.09-20.98) | 0.038 |
| Pancreas | 10/44 | 3 (1.04-8.63) | 0.042 | 2.66 (0.87-8.11) | 0.086 |
| Thyroid | 6/44 | 0.76 (0.26-2.25) | 0.62 | 0.63 (0.12-3.18) | 0.57 |
| Stomach | 5/42 | 1.1 (0.32-3.72) | 0.88 | 0.89 (0.16-4.97) | 0.89 |
| Melanoma | 6/40 | 1.06 (0.36-3.13) | 0.91 | 0.66 (0.19-2.29) | 0.51 |
| Liver | 2/19 | 3.73 (0.99-14.01) | 0.051 | NA | NA |
| Larynx | 0/17 | NA | NA | NA | NA |
| Brain | 2/14 | 4.52 (0.45-45.46) | 0.2 | 1.08 (0.1-11.2) | 0.95 |
| Esophagus | 2/14 | 1.01 (0.26-3.89) | 0.98 | NA | NA |
| Total of others **(a)** | 16/117 | 1.19 (0.66-2.14) | 0.57 | 1.06 (0.52-2.16) | 0.88 |
| **All hematologic malignancies** | 35/203 | 0.81 (0.56, 1.16) | 0.25 | 1.3 (0.8-2.11) | 0.3 |
| Lymphoma | 20/104 | 1.7 (0.93-3.1) | 0.086 | 1.81 (1-3.27) | 0.051 |
| Lymphocytic leukemia | 2/23 | 2.62 (0.47-14.71) | 0.27 | NA | NA |
| Nonlymphocytic leukemia | 10/25 | 1.71 (0.55-5.28) | 0.35 | 1.79 (0.43-7.4) | 0.42 |
| Myeloma | 1/24 | 0.33 (0.03-3.28) | 0.34 | 0.31 (0.03-3.32) | 0.34 |
| Total of others **(a)** | 2/27 | 0.71 (0.15-3.26) | 0.66 | 1.3 (0.28-6.07) | 0.74 |

**(a)** In addition to the cancers listed in this table, other cancer types also include pelvic tumors, extra pelvic tumors, and hematologic malignancies with fewer than 15 total cases (see Supplementary Table 1 for details).

**(b)** To minimize biases related to treatment assignment, patients in the No/Unknown chemotherapy group were matched to their nearest neighbors in a 1:1 ratio without replacement based on propensity scores. This approach aimed to balance the baseline characteristics between the chemotherapy and No/Unknown chemotherapy groups.

**(a)** In addition to the cancers listed in this table, other cancer types also include pelvic tumors, extra pelvic tumors, and hematologic malignancies with fewer than 15 total cases (see Supplementary Table 1 for details).

**(b)** To minimize biases related to treatment assignment, patients in the No/Unknown chemotherapy group were matched to their nearest neighbors in a 1:1 ratio without replacement based on propensity scores. This approach aimed to balance the baseline characteristics between the chemotherapy and No/Unknown chemotherapy groups.

Supplementary Table 22. Comparison of 5- to 15-Year Cumulative Incidence of Second Primary Malignancies (Accounting for Death as a Competing Risk) Among Cervical Cancer Patients Not Treated with Chemotherapy: A Comparison between Patients Diagnosed in 1975 -1999 and 2000 -2006

Supplementary Table 22. Comparison of 5- to 15-Year Cumulative Incidence of Second Primary Malignancies (Accounting for Death as a Competing Risk) Among Cervical Cancer Patients Not Treated with Chemotherapy: A Comparison between Patients Diagnosed in 1975 -1999 and 2000 -2006

**(a)** Based on the SEER database submitted in November 2023, patients diagnosed after 2006 with a minimum follow-up time of less than 15 years will no longer be included in the comparison of 5- to 15-year cumulative incidence rates among patients diagnosed in different eras.

**(b)** Due to the differences in follow-up duration among patients diagnosed in different eras, for those with a follow-up time exceeding 15 years, the outcome status is uniformly defined as 0 (indicating that no events occurred).

**(a)** Based on the SEER database submitted in November 2023, patients diagnosed after 2006 with a minimum follow-up time of less than 15 years will no longer be included in the comparison of 5- to 15-year cumulative incidence rates among patients diagnosed in different eras.

**(b)** Due to the differences in follow-up duration among patients diagnosed in different eras, for those with a follow-up time exceeding 15 years, the outcome status is uniformly defined as 0 (indicating that no events occurred).

| **Second Primary Malignancy**  **Sites** | **Cumulative Incidence**  **(5-15 Years, 95% CI) (b)** | | **Fine & Gray Competing Risks**  **Regression Model (2000-2006 (a) vs. 1975-1999)** | |
| --- | --- | --- | --- | --- |
|  | **Diagnosed between**  **1975-1999** | **Diagnosed between**  **2000-2006 (a)** | **sHR (95% CI)** | ***P* value** |
| All solid cancers (within pelvis) | 2.09% (1.85%-2.36%) | 1.09% (0.74%-1.61%) | 0.52 (0.35-0.79) | 0.002 |
| All solid cancers (Out of pelvis) | 4.30% (3.97%-4.67%) | 3.70% (3.01%-4.54%) | 0.86 (0.69-1.08) | 0.210 |
| All hematologic malignancies | 0.63% (0.51%-0.78%) | 0.52% (0.30%-0.90%) | 0.84 (0.47-1.51) | 0.570 |
| Breast | 1.65% (1.43%-1.89%) | 1.64% (1.20%-2.25%) | 1.01 (0.71-1.42) | 0.970 |
| Lung and Bronchus | 1.76% (1.54%-2.01%) | 1.00% (0.67%-1.51%) | 0.57 (0.37-0.88) | 0.011 |
