# Supplementary material for: The risk of radiation-associated second cancer in patients with cervical cancer following radiotherapy from 1975 to 2019
Source: Oncologist. 2025 Oct 10;30(11):oyaf334. doi: 10.1093/oncolo/oyaf334 (PMC12611298; doi:10.1093/oncolo/oyaf334)
Supplement: oyaf334_Supplementary_Data [file oyaf334_supplementary_data.zip › Supplementary Figure 1.docx]

**Supplementary Figure 1**


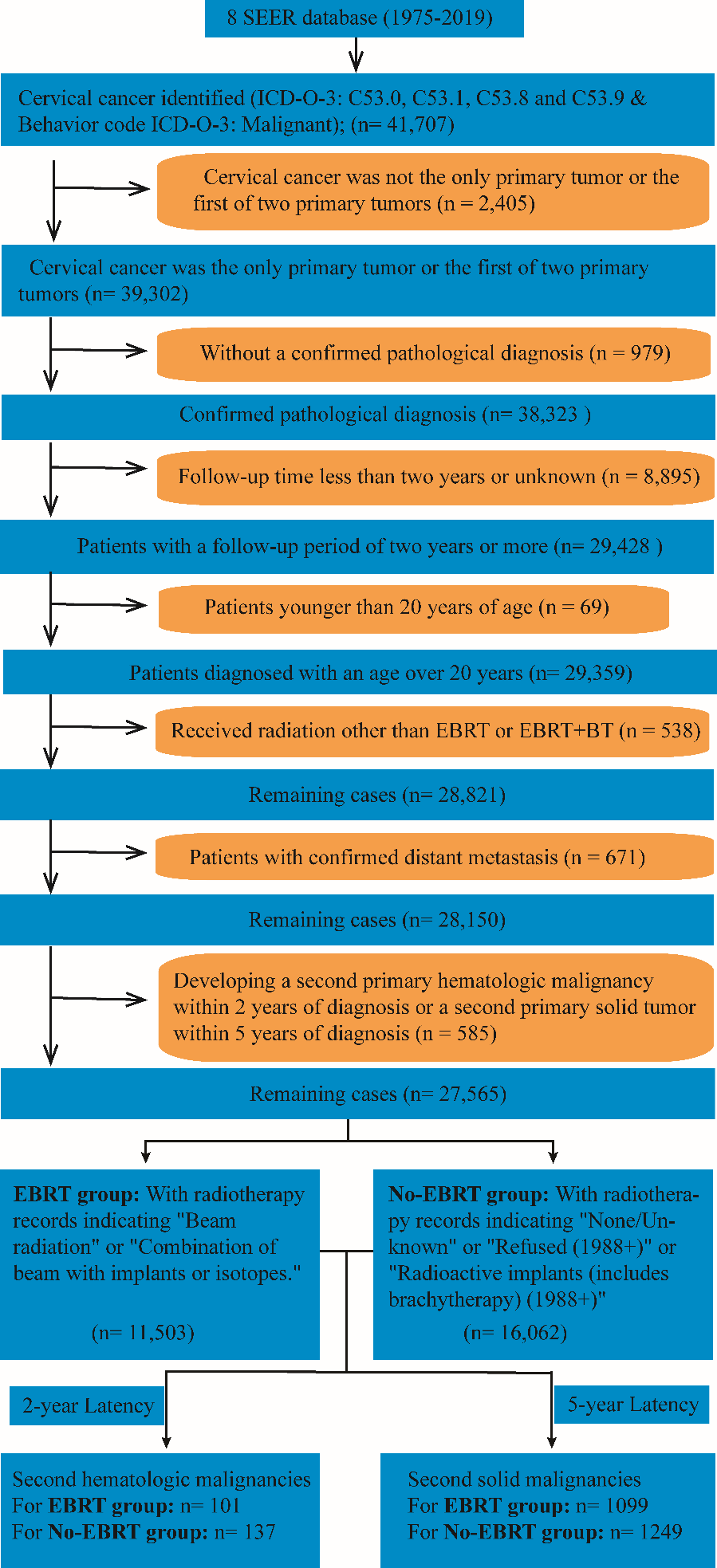


**Supplementary Figure 1.** Flow Diagram of the Study Population Selection Process. This diagram outlines the inclusion and exclusion criteria applied to identify the final cohort for analysis.
